# Mechanisms of Chemotherapy-Induced Neurotoxicity

**DOI:** 10.3389/fphar.2022.750507

**Published:** 2022-03-28

**Authors:** Halina Was, Agata Borkowska, Ana Bagues, Longlong Tu, Julia Y. H. Liu, Zengbing Lu, John A. Rudd, Kulmira Nurgali, Raquel Abalo

**Affiliations:** ^1^ Laboratory of Molecular Oncology and Innovative Therapies, Military Institute of Medicine, Warsaw, Poland; ^2^ Postgraduate School of Molecular Medicine, Medical University of Warsaw, Warsaw, Poland; ^3^ Área de Farmacología y Nutrición, Departamento de Ciencias Básicas de la Salud, Universidad Rey Juan Carlos (URJC), Alcorcón, Spain; ^4^ High Performance Research Group in Experimental Pharmacology (PHARMAKOM-URJC), URJC, Alcorcón, Spain; ^5^ Unidad Asociada I+D+i del Instituto de Química Médica (IQM), Consejo Superior de Investigaciones Científicas (CSIC), Madrid, Spain; ^6^ School of Biomedical Sciences, The Chinese University of Hong Kong, Shatin, Hong Kong SAR, China; ^7^ The Laboratory Animal Services Centre, The Chinese University of Hong Kong, Shatin, Hong Kong SAR, China; ^8^ Institute for Health and Sport, Victoria University, Melbourne, VIC, Australia; ^9^ Department of Medicine Western Health, University of Melbourne, Melbourne, VIC, Australia; ^10^ Regenerative Medicine and Stem Cells Program, Australian Institute for Musculoskeletal Science (AIMSS), Melbourne, VIC, Australia; ^11^ High Performance Research Group in Physiopathology and Pharmacology of the Digestive System (NeuGut-URJC), URJC, Alcorcón, Spain; ^12^ Grupo de Trabajo de Ciencias Básicas en Dolor y Analgesia de la Sociedad Española del Dolor, Madrid, Spain

**Keywords:** chemotherapy, neurotoxicity, chemotherapy-induced peripheral neuropathy (CIPN), chemobrain, enteric neuropathy, chemotherapy-induced nausea and vomiting (CINV)

## Abstract

Since the first clinical trials conducted after World War II, chemotherapeutic drugs have been extensively used in the clinic as the main cancer treatment either alone or as an adjuvant therapy before and after surgery. Although the use of chemotherapeutic drugs improved the survival of cancer patients, these drugs are notorious for causing many severe side effects that significantly reduce the efficacy of anti-cancer treatment and patients’ quality of life. Many widely used chemotherapy drugs including platinum-based agents, taxanes, vinca alkaloids, proteasome inhibitors, and thalidomide analogs may cause direct and indirect neurotoxicity. In this review we discuss the main effects of chemotherapy on the peripheral and central nervous systems, including neuropathic pain, chemobrain, enteric neuropathy, as well as nausea and emesis. Understanding mechanisms involved in chemotherapy-induced neurotoxicity is crucial for the development of drugs that can protect the nervous system, reduce symptoms experienced by millions of patients, and improve the outcome of the treatment and patients’ quality of life.

## Introduction

Whilst the use of certain drugs, such as pain killers, go back to ancient history, the development of anti-cancer drugs is quite recent, and they have led to a real revolution in cancer treatment. The first antitumor agent is related to World War II when the John Harvey ship carrying nitrogen mustard bombs was attacked and the toxic gas was released into the atmosphere. Those men and women who had been exposed to the gas died because of bone marrow aplasia. After the war, the mechanisms by which dichloroethyl sulfide acted were studied, thus finding that rabbits injected with high doses showed a pronounced drop in leucocyte number. After World War II, the first clinical chemotherapy trials were conducted using the analogs of mustard gases for the treatment of lymphoma (Falzone et al., 2018).

Classical antitumoral drugs are cytotoxic and act on different phases of the cell cycle, therefore, inhibiting cancer cell division (Table 1). However, they have prominent side effects, especially in fast-dividing cells such as those of the bone marrow or gastrointestinal mucosa. Although neurons are not fast-dividing cells, anti-cancer drugs may act upon them, causing neurotoxicity. Chemotherapy-induced neurotoxicity may affect any neuron in the body, both directly (direct interaction with the cell body and neurites) or indirectly (due to glial damage, inflammation and other mechanisms) and may thus cause many different symptoms affecting the quality of life of patients undergoing anti-cancer treatment.Whereas neurotoxic effects of chemotherapy on the somatic peripheral nervous system, particularly neuropathic pain, are well recognized and have been extensively studied, other entities, such as the neurotoxicity affecting the enteric nervous system (enteric neuropathy) or the brain (chemobrain), have received comparatively less attention and only recently some of the underlying mechanisms are being elucidated. Furthermore, the mechanisms involved in nausea and vomiting (which are well-known and feared adverse effects of many antineoplastic drugs) are far from clear and new exciting strategies are currently being developed to get a deeper insight into them, particularly those involving the vagus nerve.

**TABLE 1:** Main classes of cytotoxic antitumoral drugs according to their main mechanism of antiproliferative action.

Class	Mechanism of action	Cancer type	Drugs
Alkylating agents	DNA damage by producing inter and intrastrand crosslinks	Leukemia, lymphoma, Hodgkin’s disease, multiple myeloma, sarcoma, breast, lung, ovarian cancers	Cyclophosphamide
			Melphalan
			Temozolomide
			Platinum drugs
Mitotic inhibitors	Alter mitosis due to alterations in the mitotic spindle formation or function	Breast, ovarian, lung cancers, myeloma, leukemia, lymphoma	Microtubule stabilizers: taxanes
			Microtubule destabilizers: vinca alkaloids
Antimetabolites	Interfere with the synthesis of DNA or its components	Leukemia, breast, ovarian, intestinal cancers	5-fluorouracil
			6-mercaptopurine
			Cytarabine
			Gemcitabine
			Methotrexate
Anti-tumor antibiotics	Inhibit enzymes that allow DNA to be replicated	Many types of cancer	Actinomycin-D
			Bleomycine
			Anthracyclines (daunorubicin, doxorubicin)
Topoisomerase inhibitors	Inhibit topoisomerase I or II	Leukemia, lung, ovarian, intestinal cancer	Topoisomerase I: irinotecan, topotecan
			Topoisomerase II: etoposide, teniposide

Herein, we review the main effects that chemotherapy causes on the peripheral and central nervous systems, including neuropathic pain, chemobrain, enteric neuropathy, as well as nausea and emesis. The different mechanisms involved are described.

## Peripheral Neuropathy: Neuropathic Pain

The effects of the chemotherapeutic agents on the nervous system vary across the different classes of drugs, depending on their physical, chemical properties and also on their dosage (Banach et al., 2017). Importantly, compared to the central nervous system (CNS), the peripheral nervous system (PNS) is not protected by a structure similar to the blood-brain barrier (BBB), and therefore, direct toxic effects of antineoplastic drugs upon peripheral neurons are considerable, but also indirect effects contribute, mainly due to inflammatory reactions, leading to the development of chemotherapy-induced peripheral neuropathy (CIPN).

CIPN is a highly prevalent, severe, and dose-limiting toxicity, resulting in dose interruptions, subtherapeutic dosing, or discontinued therapy (Staff et al., 2017). Acute symptoms of CIPN appear in the hours and days after drug infusion, whereas persistent symptoms occur in approximately 68% of patients 1 month following completion of chemotherapy and in 30% of patients 5 months later (Omran et al., 2021). Neuropathic pain is characterized by the presence of allodynia and hyperalgesia due to the decrease of the sensitivity threshold for non-noxious and noxious stimuli, respectively. Remarkably, also spontaneous pain can be present in some patients. Likewise, peripheral motor nerves may also be affected by some drugs, leading to motor impairments (Zajączkowska et al., 2019). A summary of the clinical symptoms induced by the main antitumoral drug classes underlying CIPN is shown in Table 2.

**TABLE 2:** Chemotherapy induced neuropathy and clinical symptoms induced by representative antitumoral drugs.

Drug	Type of neuropathy	Clinical symptoms
Cisplatin/oxaliplatin	Pure sensory	Paresthesia
		Dysesthesia
		Neuropathic pain in a stocking-and-glove distribution
Acute oxaliplatin	
		Paresthesia
		Muscle tightness
		Cramps
Paclitaxel	Mixed sensory—motor	Paresthesia
		Hypoesthesia
		Neuropathic pain in a stocking-and-glove distribution
		Myalgia, myopathy
Vincristine	Mixed sensory-motor and autonomic	Paresthesia
		Hypoesthesia
		Neuropathic pain in a stocking-and-glove distribution
		Muscle cramps
		Mild distal weakness
		Enteric neuropathy
		Autonomic dysfunctions
Bortezomib and thalidomide	Sensory-motor	Neuropathic pain
		Hypoesthesia
		Paresthesia in distal extremities of limbs
		Muscle cramps
Bortezomib	Sensory-motor (rare) and autonomic	Paresthesia
		Painful sensory neuropathy in distal extremities of limbs

Many widely used chemotherapy drugs, including platinum-based agents (oxaliplatin, cisplatin, carboplatin), taxanes (paclitaxel, docetaxel), vinca alkaloids (vincristine, vinblastine), proteasome inhibitors, and thalidomide analogues may cause direct neurotoxicity and CIPN (Zajączkowska et al., 2019). Knowing how these agents interact with the nervous system is crucial for the development of drugs that can protect against peripheral neuropathy.

**FIGURE 1:**
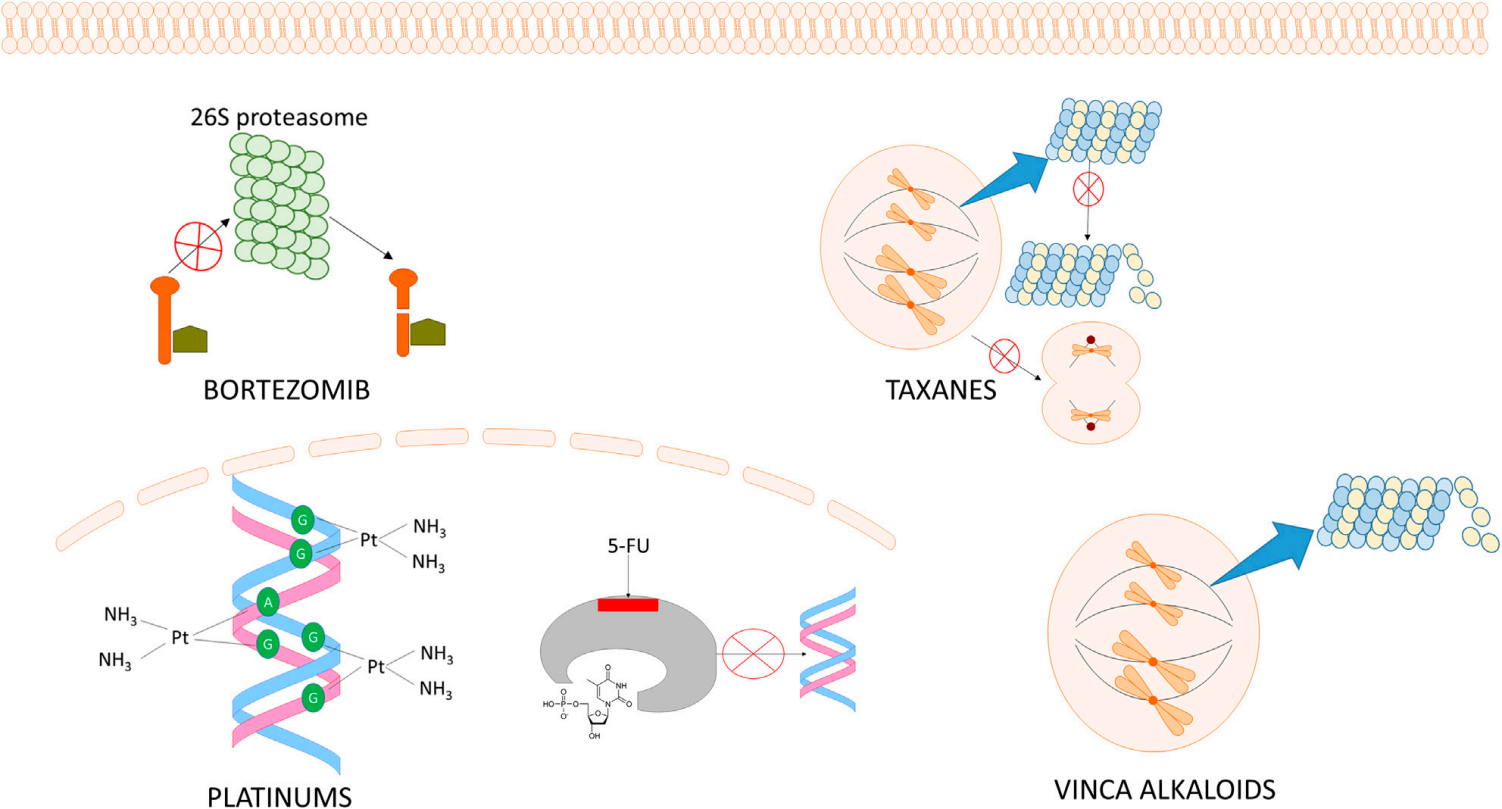
Schematic representation of the mechanisms of action of the main antitumoral drugs that cause direct neurotoxicity and peripheral neuropathy. 1) Bortezomib inhibits the 26S proteasome. 2) Taxanes stabilize the tubulin proteins, therefore anaphase cannot be achieved. 3) Vinca alkaloids de-stabilize the microtubules, thus the mitotic spindle cannot be formed. 4) Platinum-based compounds form intra-strand and cross-strand links. 5) 5- Fluorouracil (5-FU) binds to thymidylate synthase (TS). Both platinum-based compounds and 5-FU inhibit DNA synthesis.

**FIGURE 2:**
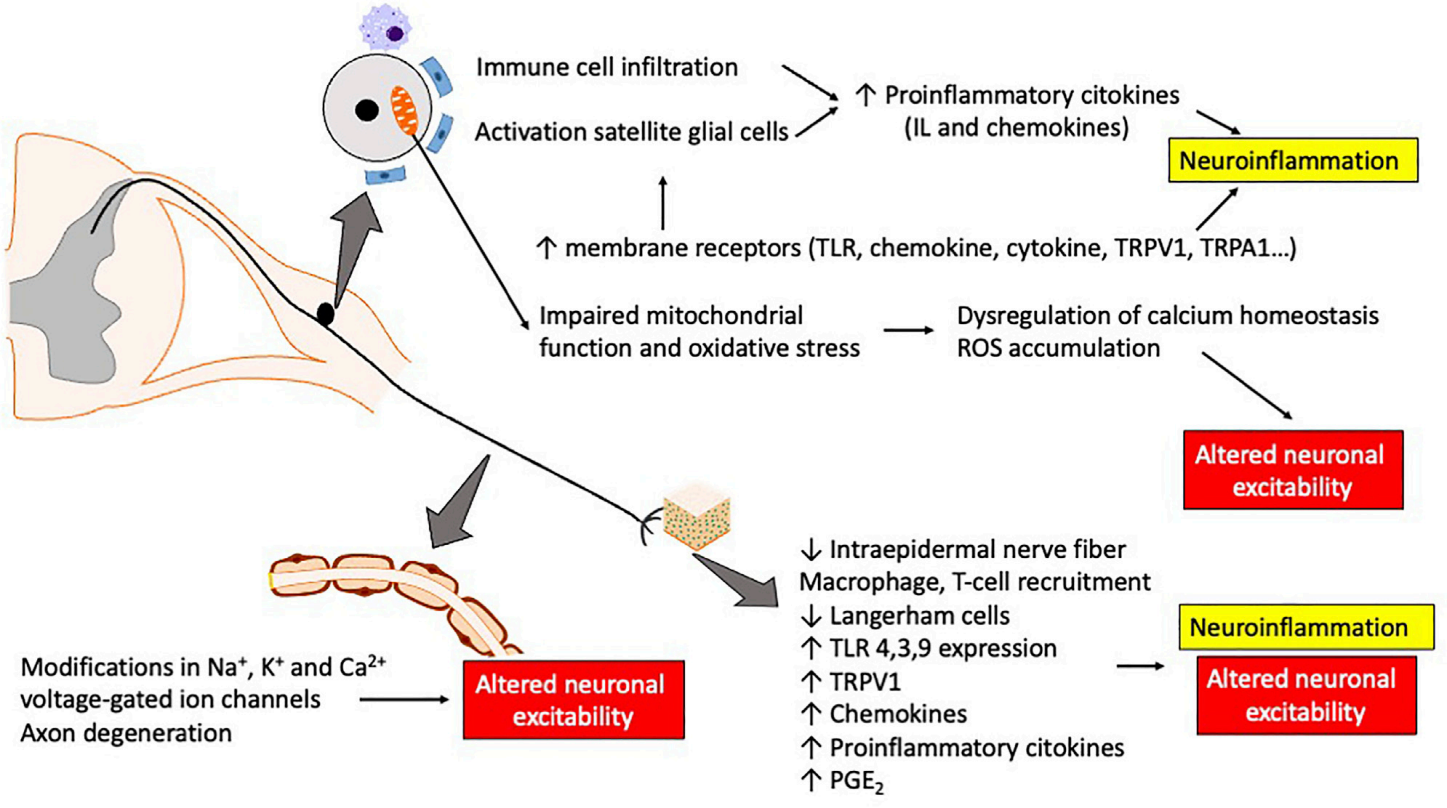
Schematic representation of the main structural alterations, cytokine release and modifications in ion channels and receptors induced by antitumoral drugs which cause neuroinflammation and altered neuronal excitability. Abbreviations: IL, interleukins; PGE_2_, prostaglandin E_2_; ROS, reactive oxygen species; TLR, toll-like receptors; TRP, transient receptor potential.

Although the neuropathic symptoms induced by the different chemotherapeutic agents have been classically related with their mechanism of action underlying their antiproliferative effect (Figure 1), new evidence seems to demonstrate the contribution of numerous off-target players which may also contribute to structural damage, mitochondria dysfunction and the release of different pro-inflammatory cytokines amongst others (Figure 2) (Montague and Malcangio, 2017; Zajączkowska et al., 2019; Pellacani and Eleftheriou, 2020; Fumagalli et al., 2021; Vermeer et al., 2021; Yamamoto and Egashira, 2021). Thus, the direct mechanism of action of the different antitumor agents and how they differ regarding the alterations they induce in the peripheral nervous system (PNS) causing peripheral neuropathy (or not, in some cases) will be reviewed below, with special emphasis on the activation of the innate immune system and the release of chemokines, as recently proposed as an initiating factor for CIPN.

### Platinum-Based Compounds

The first antitumoral agent of this group was cisplatin, patented in 1978 for the treatment of different types of solid tumors. However, due to its side effects and the appearance of cell resistance, carboplatin (a second-generation platinum agent) and oxaliplatin (a third-generation platinum agent) are nowadays the most frequently used in the clinic (Fischer and Robin Ganellin, 2006).

Platinum agents are intravenously administered and enter the cell through passive diffusion, but also through active transport (Johnstone et al., 2016). Once entered, all platinating agents become aquated, and, although they interact with ribonucleic acid (RNA) and proteins, the main target is deoxynucleic acid (DNA). All platinum agents bind preferentially to guanosine and adenosine, to form intrastrand and interstrand crosslinks. This results in restriction of DNA replication, transcription, cell cycle arrest, and programmed cell death (Rabik and Dolan, 2007). Several studies have demonstrated that although the mechanisms by which cisplatin and carboplatin act are similar, differences are observed with oxaliplatin, the latter showing fewer platination lesions of DNA, with the same cytotoxic effect as cisplatin at equimolar concentrations. Additionally, oxaliplatin adducts are bulkier and more hydrophobic than those formed from cisplatin or carboplatin. The fact that oxaliplatin is more efficient than cisplatin could be explained by the fact that DNA-oxaliplatin adducts are more effective in inhibiting DNA synthesis and less susceptible to the repair mechanisms of the cell (Dilruba and Kalayda, 2016; Schoch et al., 2020). Mechanisms activating the immune system have been proposed to act additively to the inhibition of DNA replication, although these remain to be elucidated (De Biasi et al., 2014).

Because platinum drugs do not cross the BBB, the dorsal root ganglia (DRG), involved in somatic and visceral sensitivity, as well as the area postrema (AP), involved in nausea and emesis (see *Peripheral Mechanisms in Nausea and Emesis: Unresolved Issues for Exploration*), which are vascularized by fenestrated capillaries, are the main targets of platinum compounds in the nervous system. The main mechanism by which platinum compounds induce neuropathy is mainly due to the DNA adducts they form in the nucleus of the neurons. Some of these adducts are less efficiently repaired by the nucleotide excision repair pathway (NER), thus the DNA-platinum adducts that are not removed by NER do not allow correct transcription of the ribosomal RNA. DRG neurons are cells with high metabolic activity and so the lack of physiological dense ribosomal RNA synthesis may be lethal for this cell type (Dzagnidze et al., 2007). Oxaliplatin induces less sensory neuropathy than cisplatin (André et al., 1999; de Gramont et al., 2000), this has been proven to be related to a reduced number of oxaliplatin adducts in the DRG when compared to cisplatin at equimolar concentrations (Ta et al., 2006). Additionally, carboplatin is less neurotoxic at conventional doses than cisplatin, which could also be due to lower intracellular platinum accumulation and platination (Schoch et al., 2020).

Platinum compounds have also the ability to bind to mitochondrial DNA (mtDNA), which cannot be repaired because there are no DNA repair systems in mitochondria, thus increasing the amount of reactive oxygen species (ROS) and oxidative stress (Podratz et al., 2011). Studies performed *in vivo* and *in vitro* showed functional and morphological damage after platinum exposure; mitochondria appeared swollen and vacuolated with impaired mtDNA replication and ATP production (Canta et al., 2015). Additionally, mitochondria are localized in the axons of the neurons of the PNS, thus causing alterations in the axonal transport; this phenomenon is thought to be involved in the onset of neuropathic pain (Chiorazzi et al., 2015). Mitochondria and endoplasmic reticulum are internal storages for Ca^2+^; when damaged, intracellular levels of Ca^2+^ are increased, with the consequent alteration in neuronal excitability and activation of calpain causing axonal degeneration (Wang et al., 2012).

Besides the chronic neuropathy, oxaliplatin presents a unique characteristic: its administration associates with an acute and transient neuropathy, which consists of muscle tightness, cramps, paresthesias, and dysesthesias in the limbs and perioral region that are worsened by cold (Lehky et al., 2004; Wolf et al., 2008; Lucchetta et al., 2012). The mechanisms of this phenomenon seem to be due to acidification of intracellular pH of DRG neurons, which does not occur in cells treated with cisplatin at equimolar concentrations. The authors hypothesize that the acidification hypersensitizes the transient receptor potential (TRP)A1 channel but could also be explained by the participation of TRPM8 and TRPV1 channels, which have been implicated in this side effect (Riva et al., 2018).

Growing evidence suggests an important neuro-inflammatory contribution in the development of peripheral neuropathy and structural damage caused by the direct mechanism of antitumoral agents. Although the participation of the immune system has been more profoundly studied in paclitaxel-induced neuropathy (see below), a previous study demonstrated that a single administration of oxaliplatin increased the levels of the chemokine, CCL2, and its receptor, CCR2, at the same time as mechanical hypersensitivity developed; pretreatment with the CCL2 antibody predictably prevented the development of allodynia (Illias et al., 2018). The chemokine, CX3CL1, has also been shown to be upregulated in DRG neurons and contributes to thermal hyperalgesia and an increase in the number of action potentials in small diameter neurons of the DRG in a model of oxaliplatin-induced chronic neuropathy. The increase in CX3CL1 was mediated through nuclear factor κB (NF-κB) pathways (Wang et al., 2017). Additionally, the chemokine, CXCL12, has been shown to be upregulated following chronic oxaliplatin administration and contributes to mechanical and thermal hypersensitivity via the TNF-α/IL-1β–dependent STAT3 pathway.

High mobility group box-1 (HMGB1) is a DNA binding nuclear protein that activates toll-like receptor (TLR) 4 signalling and induces inflammatory processes. Curiously, an anti-HMGB1 antibody reversed mechanical but not cold hypersensitivity induced by oxaliplatin treatment. Antagonism of HMGB1 receptors, TLR4, receptor for advanced glycation end products (RAGE) and CXCR4, prevented the appearance of mechanical allodynia, whilst only RAGE was overexpressed in the DRG; no modifications of TLR4, CXCR4 and RAGE were observed in the sciatic nerve (Tsubota et al., 2019). Additionally, and in contrast to paclitaxel treatment, oxaliplatin did not increase macrophage infiltration or accumulation in the sciatic nerve, and treatment with a macrophage depletor did not prevent or reverse allodynia in mice, but pretreatment with thrombomodulin (an endothelial transmembrane protein that sequestrates and degrades HMGB1) did prevent oxaliplatin-induced sensory neuropathy (Tsubota et al., 2019). Thus, the authors proposed that HMGB1 derived from non-macrophage cells mediates oxaliplatin-induced neuropathy (Tsubota et al., 2019). On the contrary, an increase in macrophage infiltration into DRGs did occur after repeated oxaliplatin administration, where both neurons and macrophages secreted matrix metallopeptidase (MMP) 9-2 (proteolytic enzymes involved in neuroinflammation and chronic pain), by activating TLR4 through HMGB1 protein; this mechanism contributes to the onset of mechanical hypersensitivity through the HMGB1-TLR4-PI3K/Akt-MMP9 axis (Gu et al., 2020). These data demonstrate that although the symptoms that these drugs induce are similar, the initiating factors are different, thus future drugs with different and more selective action mechanisms can be developed.

Additionally, an acute administration of oxaliplatin upregulates the phosphoinositide 3-kinases - mammalian target of rapamycin receptor (PI3K-mTOR) pathways and the resultant expression of cytokines, interleukin (IL)-6, IL-1β and tumor necrosis factor (TNF)-α, whilst inhibiting PI3K signalling reduces mechanical and cold hypersensitivity and the levels of proinflammatory cytokines (Duan et al., 2018).

Sex dimorphism has been shown to modify the response to platinum-based chemotherapeutics. The neuroendocrine stress axis plays an important role in the development of neuropathy, although with different implications. Whilst adrenalectomy and corticoid receptor downregulation reduced and prevented oxaliplatin-induced hyperalgesia in both sexes, the downregulation of β_2_ adrenergic receptor prevented and reversed oxaliplatin-induced hyperalgesia only in males. Moreover, the type of induced stress also resulted in sex-dependent differences in oxaliplatin-induced peripheral sensory neuropathy (Staurengo-Ferrari et al., 2021).

Recently, the role of gut microbiota has been implicated in the development of oxaliplatin-induced peripheral sensory neuropathy. Chronic administration of oxaliplatin did not induce mechanical hypersensitivity in mice nor spontaneous pain behaviour in rats pretreated with antibiotics, nor in germ-free mice, whilst neuropathy did appear in control animals (Shen et al., 2017). The germ-free animals presented lower levels of IL-6 and TNF-α than the corresponding controls, and lower levels of ROS and less macrophage infiltration in the DRG, whilst these differences were not observed in the spinal cord and the major immune cells in peripheral blood were unaffected. The authors found that microbiota influenced the development of mechanical oxaliplatin-induced hypersensitivity through a lipopolysaccharide (LPS)-TLR4 pathway and the expression of TLR4 on hematopoietic cells seems to be responsible, at least partially, for this effect (Shen et al., 2017).

### Taxanes

The name taxanes comes from the Latin term of yew *Taxus sp*. as they are found in these plants. Paclitaxel (PCTX) was the first drug of this group isolated from the stem bark of the Pacific yew tree in 1960, although it was not until 1994 that it was approved for its use in ovarian cancer treatment (Menzin et al., 1994). Docetaxel (DCTX) is a semisynthetic paclitaxel derivative. Both drugs are very insoluble; they require different solvents, which frequently induce allergic reactions. Thus, research into a new generation of taxanes aims to obtain molecules with better therapeutic and toxicity profiles and higher solubility (Velasco and Bruna, 2015).

Taxanes are chemically very similar and therefore have a similar mechanism of action, which is dose-dependent. At high concentrations, PCTX binds to the β-tubulin subunit, which promotes the assembly of tubulin-enhancing microtubule polymerization. The formation of altered mitotic spindles prevents normal mitosis and cells undergo apoptosis. On the other hand, small concentrations of PCTX do not increase microtubule polymerization, but act as microtubule-stabilizing agents, blocking depolymerization, thus the anaphase cannot be achieved and apoptosis mechanisms are activated (Tamburin et al., 2019).

DCTX has shown to be a more potent drug and more active in preclinical studies; it also has a higher affinity for the binding site in the β-tubulin subunit, although this has not been shown to translate into increased survival rates in clinical studies where PCTX and DCTX are administered with other antitumoral drugs (Melchior et al., 2018). Although chemically quite similar, DCTX and PCTX show certain differences in the mechanism of action. The tubulin polymers generated by DCTX are structured differently than those of PCTX, and DCTX does not change the number of the protofilaments in the microtubules (Verweij et al., 1994).

Most previous studies have demonstrated that the incidence of PCTX-induced neuropathy is higher than that of DCTX (Stubblefield et al., 2009; Song et al., 2017), although certain controversy exists as this is not always observed (Velasco and Bruna, 2015). Neuropathy caused by taxanes mostly affects small-diameter sensory fibers, inducing altered proprioception and paresthesias, dysesthesias, and numbness in a stocking-glove distribution. Accordingly, PCTX treatment has been associated with a reduction in the sensory nerve action potential and a reduction of nerve conduction velocity (Boehmerle et al., 2014). Motor and autonomic dysfunction are less likely to occur (Pascual et al., 2005; De Iuliis et al., 2015).

Axons of sensory neurons have a high activity in retrograde and anterograde transport of different molecules, which is necessary for their survival and is microtubule-dependent (Nirschl et al., 2017). Several studies have pointed out that alterations in the microtubule structure induced by taxanes could impair axonal transport and, therefore, cause the degeneration of distal nerve segments (Hagiwara and Sunada, 2004; Gornstein and Schwarz, 2014). However, this theory is in dispute because through *in vitro* techniques using microfluidic chambers, Gornstein and Schwarz have recently found that the distal part of the axon is selectively sensitive to PCTX (Gornstein and Schwarz, 2017). This finding is in agreement with that of Silva et al. (2006) who found that low doses of vincristine (a vinca alkaloid, see below) were toxic at the distal part of the axon but not the mid-region (Silva et al., 2006). Thus, a selective vulnerability of the distal axon could be a commonality for microtubule-targeting agents, leading to axonal demyelination and the loss of intraepidermal nerve fibres (IENF) in the hindpaw skin of rodents (Siau et al., 2006; Wozniak et al., 2018; Vermeer et al., 2021). This finding is also supported by the fact that mitochondrial damage is more intense in the distal axons when compared to more proximal ones (Bobylev et al., 2015; Vermeer et al., 2021). Additionally, studies have observed PCTX-induced modifications of acetylated tubulin, but tubulin expression returns to control levels rapidly after cessation of drug treatment, whilst morphological effects persist long after the treatment (Cook et al., 2018).

Interestingly, although PCTX does not cause direct mitochondrial DNA damage, mitochondria have been suggested as potential mediators for PCTX toxicity, as swollen and vacuolated mitochondria have been observed in myelinated and unmyelinated sensory nerves, thus the authors conclude that alterations in mitochondria contribute to PCTX-induced pain instead of dysfunctions of axonal microtubules (Flatters and Bennett 2006; Staff et al., 2017). Furthermore, mitochondria pathology has been related to taxane administration, possibly causing oxidative stress and the production of ROS leading to the activation of apoptotic processes, disruption of cell structure, and demyelination. As with platinum, taxane-induced mitochondria pathology also causes an efflux of calcium from the mitochondria and endoplasmic reticulum, inducing axon degeneration and DRG cell apoptosis (Flatters and Bennett, 2006; Xiao et al., 2012).

In the clinical setting, the coadministration of taxanes and platinum is frequent. It has the advantage of lowering the appearance of resistance and the side effects. Curiously, although PCTX administration after completion of the carboplatin regimen seems to induce more severe neuropathy in mice (Toma et al., 2017), the combined regimen of PCTX and oxaliplatin does not worsen the neuropathy induced by each antitumor drug separately in the rat (Paniagua et al., 2021). Further research should clarify whether these differences are due to drug regimen, differences in species, or possibly different mechanisms of neuropathy induction.

Previous studies have demonstrated that macrophage infiltration in the DRG coincides with the development of cold and mechanical hypersensitivity after PCTX treatment. Additionally, cotreatment of PCTX with a macrophage toxin prevents the appearance of this neuropathy (Peters et al., 2007; Zhang et al., 2016). Remarkably, the infiltration of macrophages in the DRG occurred at the same time as mechanical hypersensitivity developed. Depletion of the macrophages partially reversed this mechanical hypersensitivity and reduced the expression of the proinflammatory cytokine TNF-α, as did the injection of the MCP-1 (methyl-accepting chemotaxis proteins -1, whose receptor is CCR2) neutralizing antibody. Importantly, a TLR4 antagonist attenuated the mechanical hypersensitivity, reduced MCP-1 expression, and blocked the infiltration of macrophages, and both the TLR4 antagonist and the macrophage toxin prevented the alterations of IENF endings. In line with this finding, PCTX upregulated CXCL1 in a time-dependent manner in myelinated A and C fibers but not in satellite glial cells, and the intrathecal injection of neutralizing antibodies against CX3CL1 or CX3CR1 and the macrophage toxin significantly reduced macrophage infiltration and delayed hypersensitivity induced by PCTX (Huang et al., 2014). In addition, the inhibitor of NF-kβ significantly reduced the upregulation of CX3CL1 proteins and the mechanical allodynia induced by PCTX (Li et al., 2015).

During the last years other chemokines which bind to the CXCR2 receptor have been studied, such as CXCL1, although curiously the systemic administration of the CXCL1 antibody was much less effective in reducing the PCTX-induced peripheral neuropathy than intrathecal administration, possibly indicating different chemokine involvement in the PNS and central nervous system (CNS) (Manjavachi et al., 2019).

As CIPN induced by platinum-based chemotherapeutics, CIPN induced by PCTX seems to be influenced by factors such as sex and microbiome. Thus, recent studies have shown that TLR9 mutation attenuated the development of neuropathic pain caused by PCTX, and inhibited CXCL1 and TNF-α upregulation in cultures of DRG-derived macrophages obtained from male mice (Luo et al., 2019). Also, IL-23 promotes the release of IL-17A from macrophages, which in turn activates nociceptors through the TRPV1 receptor, and this effect was prevented by ovariectomy and induced by the administration of estrogen. When collecting serum and the DRG from PCTX-treated mice, the authors found an increase in IL-23 only in female mice. The early and late phases of mechanical allodynia were reduced in IL-23 receptor knockout mice. Furthermore, the ablation of macrophages prevented the mechanical hypersensitivity mediated through IL-23 induced by PCTX.

With regards to the microbiome, *in vitro* studies have demonstrated that the combined treatment of PCTX and a probiotic normalizes the expression of TRPV4 and acetylated α-tubulin which are overexpressed in PCTX-treated hybridoma F11 cells (an *in vitro* model of sensitive DRG neurons). The mechanism by which this occurs is by restoring the control conditions of different pathways leading to peripheral sensory neuropathy (Castelli et al., 2018). The finding that mice treated previously and during PCTX administration with a probiotic formulation prevented the mechanical hypersensitivity development further validated this study. *Ex vivo* analyses showed that probiotics reduced colon tissue damage, upregulated the levels of cannabinoid and opioid expression in the spinal cord, reduced the expression of inducible nitric oxide synthase (iNOS) and cyclooxygenase (COX)-2 and peroxisome proliferator-activated receptor γ (PPAR γ) and reduced serum levels of TNF-α, IL-1 and IL-6 when compared to PCTX treatment (Cuozzo et al., 2021).

### Vinca Alkaloids

Vinca alkaloids are also naturally derived molecules, which are found in the Madagascar periwinkle *Catharanthus roseus* leaves. Vincristine was discovered in an attempt to find hypoglycemic drugs for the treatment of diabetes. However, studies in mice demonstrated that instead of lowering glycemia, it decreased the number of leukocytes, thus opening a new possibility for the treatment of leukemia. Like taxanes, vinca alkaloids are microtubule–targeting agents, although instead of stabilizing the structure they bind to the β-tubulin subunit, preventing the straightening of the structure of the molecule, thus interfering with the polymerization with microtubular α-tubulin (Ravelli et al., 2004; Van Vuuren et al., 2015).

Vinca alkaloids include vincristine, vinblastine, and vinorelbine. All of them induce sensory-motor neuropathy, although vincristine induces the most severe neurotoxicity, possibly due to the differences in the potency and affinities to the binding site. The differential binding to individual tubulin isotypes may explain the differences in the toxicities (Lobert et al., 1996). Thus, vincristine doses are limited by the drug-induced neurotoxicity, whereas vinblastine and vinorelbine doses are limited by bone marrow toxicity (Lobert et al., 1996).

The mechanisms by which vincristine induces alterations in the neuron structure are similar to those of PCTX, including disruption of microtubules, leading to altered axonal transport and interference with normal mitochondrial function, which in turn causes altered calcium homeostasis and increased release of ROS, both causing axon degeneration and abnormal myelination (for review see Triarico et al., 2021). As in the case of taxanes, there seems to be contradictory results whether peripheral neuropathy can be due to axonal transport disruption or higher sensitivity of the distal axon to especially vincristine, as stated above (Silva et al., 2006).

Additionally, activation of the immune system has also been observed. Thus, Old et al. (2014), found that the administration of 2 consecutive cycles of vincristine (5 days of consecutive treatment in each cycle) induced mechanical hypersensitivity, but did not induce DRG, axon damage nor alterations in the microglia of the peripheral nerves, although by the end of the treatment there was an increase in the number of monocytes expressing the CX3CR1 receptors in the sciatic nerve. When vincristine was administered in CX3CR1 knockout mice, mechanical hypersensitivity was delayed and the activation of CX3CR1 monocytes by fractalkine induced ROS production which in turn activated the TRPA1 channel in sensory neurons (Old et al., 2014). Curiously, CCR2 knockout mice developed similar initial neuropathy to that in control littermates, whilst during the second cycle CCR2-deficient mice had less mechanical neuropathy than controls, and also presented less expression of the macrophage marker than the controls. Additionally, in CX3CR1 deficient mice allodynia was only developed during the second week and was reversed by treatment with the CCR2 antagonist, which also reduced the macrophage infiltration in the sciatic nerve. The downregulation of CX3CR1 resulted in an increase in the release of the proinflammatory cytokines, TNF-α and IL1-β, which could in turn mediate noxious signaling following TNF-α receptor activation in sensory neurons (Montague et al., 2018).

### Bortezomib

Bortezomib is an inhibitor of the proteasome, which was discovered relatively recently compared to the above mentioned chemotherapeutic agents (Adams, 2002). Afterwards, it was approved for the treatment of multiple myeloma in 2003; and over the years, it has been used for the treatment of different hematologic cancers.

26S proteasome is the major protease in eukaryotic cells, it is a large multiprotein complex, which degrades damaged or obsolete proteins, with a 19S regulatory subunit and a 20S catalytic domain. When proteins need to be degraded, they are marked with ubiquitin, which binds to the receptor on the 19S complex and are introduced into the catalytic chamber in the center of the 20S subunit, consequently, these proteins are broken into small polypeptides. Bortezomib inhibits the 20S core proteasome, resulting in cancer cell death via multiple mechanisms, including suppression of the unfolded protein response, accumulation of ubiquitinated proteins, stabilization of tumor suppressor proteins, such as p21, p27, Bax and p54, and induction of ROS (Geisler, 2021).

One of the most severe side effects of bortezomib is peripheral neuropathy, which is very painful (Saifee et al., 2010). The exact mechanism by which bortezomib induces peripheral neuropathy has not been elucidated. In the peripheral nerves, bortezomib has been shown to reduce nerve conduction velocities and alter nerve action potentials in animals and humans (Cavaletti et al., 2007; Carozzi et al., 2010; Alé et al., 2014; Bechakra et al., 2018), although differences in animal and human results exist in whether it induces IENF alterations (Bechakra et al., 2018; Meregalli et al., 2018). Additionally, chronic administration of bortezomib in animals induces degeneration of myelinated and unmyelinated axons of the caudal and sciatic nerves, vacuolization and detachment of satellite glial cells and an upregulation of substance P (SP) and calcitonin gene-related peptide (CGRP) Il-6, TNF-α, transforming growth factor (TGF)-β1 and IL-1β in the DRG (Carozzi et al., 2010, 2013; Ale et al., 2014; Quartu et al., 2014; Meregalli et al., 2018; Liu et al., 2019). Some studies have found that it can cause alterations in DRG satellite cells leading to an immune-mediated, demyelinating neuropathy (Pitarokoili et al., 2017; Xu et al., 2019). Additionally, this drug induces axonal membrane depolarization prior to the onset of neuropathy, which seems to be due to the impairment of the Na^+^/K^+^ ATPase pump, caused by mitochondrial dysfunction (Nasu et al., 2014). Alterations in calcium homeostasis, autoimmune inflammatory processes, and blockade of nerve growth factor (NGF) mediated neuronal survival have also been implicated in peripheral neuropathy induced by bortezomib (Landowski et al., 2005; Yin et al., 2018).

Vincristine, PCTX, and bortezomib cause axonal degeneration although through a different mechanism than those triggered by microtubule-stabilizing agents. *In vitro* experiments have demonstrated that bortezomib does not induce microtubule bundles in contrast to PCTX-treated ones, suggesting microtubule stabilization occurs via an indirect-induced neuropathy mechanism and not directly through drug binding (Poruchynsky et al., 2008). In addition, in *in vitro* studies vincristine induces a degeneration of axon when directly in contact with it, but not when in contact only with the cell body, whilst the opposite occurs with bortezomib. Axon degeneration induced by vincristine is dependent on mitogen-activated protein kinase (MAPK) pathways, whilst in the case of bortezomib it is dependent on the activation of axonal caspases. Both mechanisms finally converge in a common pathway, similar to Wallerian degeneration in the case of vincristine and apoptosis after bortezomib administration (Geisler et al., 2019). These results are in accordance with those of Landowski et al., who found that dysregulation of mitochondrial calcium after bortezomib administration triggered caspase 12 activity (Landowski et al., 2005). Furthermore, bortezomib-induced peripheral neuropathy has been associated with impairment of the mitochondrial function, with a deficiency in ATP production and increased ROS production and an increase of vacuolated and swollen mitochondria in A and C fibers (Zheng et al., 2012; Duggett and Flatters, 2017), although whether vacuolated mitochondria appear in the DRG remains controversial (Cavaletti et al., 2007; Meregalli et al., 2010).

With regards to the innate immune response and chemokine release, very little has been published. CCL1 and CCL2 have been shown to be upregulated in the DRG. Whereas CCL1 increased rapidly after initiating bortezomib treatment, CCL2 increased transitorily and in a similar pattern to the development of allodynia. CCL2 was overexpressed in neuronal cell bodies but not satellite glial cells, with infiltration of macrophages in the DRG. The authors demonstrated that CCL2 was upregulated through the recruitment of the transcription factor, c-Jun, to the ccl2 gene promoter, and this was facilitated by activating transcription factor 3 (ATF3) (Liu et al., 2016). In line with the previous studies, bortezomib caused mechanical allodynia in mice which was prevented by the anti-HMGB1 neutralizing antibody. As previously mentioned, HMGB1 can bind to different receptors: RAGE, CXCR4 and TLR4. The receptor antagonist for RAGE and CXCR4 completely abolished the bortezomib-induced allodynia, but only the RAGE antagonist transiently reversed the allodynia once it had established; a TLR4 antagonist had no effect. Minocycline and ethyl pyruvate (which inhibits HMGB1 release from macrophages) and macrophage depletion with liposomal clodronate prevented the appearance of neuropathy, but only minocycline abolished the established neuropathy. Curiously, the caspase 8 inhibitor significantly reduced bortezomib-induced macrophage release of HMGB1, preventing neuropathy. Thus, in contrast to data from the PCTX model, HMGB1 release from macrophages was not mediated through NF-κB and p38MAPK but instead through a caspase-dependent mechanism (Tsubota et al., 2019).

### Thalidomide

Thalidomide is a synthetic glutamic derivative, it was approved for pregnancy-associated morning sickness in 1957, although a few years later it was withdrawn because of teratogenicity. In the late 1990s, it was reintroduced for the treatment of certain types of cancer due to its antiangiogenic and pro-apoptotic effects (Fernyhough et al., 2005). The anti-cancer mechanisms of thalidomide are not well known, although they include the blocking of the production of TNF-α, and the blocking of the activation of NF-κB, accelerating cell death.

The incidence of thalidomide-induced neuropathy is relatively high, occurring in 25–75% of patients. Generally, it is a classical sensory neuropathy, although at high doses it can also induce motor impairment. Autonomic neuropathy also occurs frequently affecting 80–90% of patients (Briani et al., 2004; Glasmacher et al., 2006). The mechanisms by which thalidomide induces the antitumor effect have been postulated to cause this neuropathy: the antiangiogenic mechanism would cause secondary hypoxia and ischemia of nerve fibers, whilst inhibition of TNF-α and the blocking of the activation of NF-κB would accelerate neuronal cell death by dysregulating the neurotrophins and their receptors (Keifer et al., 2001). These mechanisms need further research as thalidomide has been demonstrated to reduce hyperalgesia in other models of neuropathic pain in a TNF-α dependent mechanism (Nascimento et al., 2015; Zajączkowska et al., 2019).

### 5-Fluorouracil

5-fluorouracil (5-FU) is one of the oldest chemotherapy agents, synthesized by Heidelberg et al. in 1957. It is a molecule that is produced by substituting a hydrogen atom of uracil for a fluorine atom. It acts as an antimetabolite inhibiting the thymidylate synthase (TS), which is a key enzyme in the synthesis of 2′-deoxythymidine-5′-monophosphate, required for DNA synthesis, therefore DNA synthesis and repair is ultimately inhibited. 5-FU is also capable of binding to DNA, thus altering the sequence of nucleotides. Finally, at higher doses, dysfunction of RNA can be caused, thus interfering with the synthesis of cellular proteins and leading to apoptosis (Tanaka et al., 2005).

5-FU poorly permeates biological membranes. Thus, a high dose needs to be administered to reach effective intracellular concentrations. It has a narrow therapeutic index with a high incidence of side effects (Ewert de Oliveira et al., 2021). Some of the most common are mucositis, diarrhea, or myelosuppression. Although generally unrecognized, due to the fact that other antitumoral drugs with which 5-FU is often administered (i.e., oxaliplatin, for colorectal cancer treatment, McQuade et al., 2016a) exert clearer neurotoxicity, 5-FU may induce CIPN, as the signs of hyperalgesia and allodynia have been detected in rats treated with a single dose of the compound (even at low doses causing mucositis but not frank diarrhea; unpublished observations). Importantly, unlike the above-mentioned antitumoral agents, 5-FU does cross the BBB (Fournier et al., 2003), causing CNS neurotoxicity as described below.

### Methotrexate

Methotrexate (MTX) is an anti-metabolite of folic acid. It blocks the enzyme dihydrofolate reductase, which catalyzes the dihydrofolate to tetrahydrofolate. Because tetrahydrofolate acts as a coenzyme for several pathways in purine and pyrimidine nucleotide synthesis, DNA repair and replication are disrupted (Koźmiński et al., 2020). MTX does not cross the BBB at low doses, although at high doses it has modest permeability (Myers et al., 2008). The mechanisms by which MTX is neurotoxic remains unclear. Abnormal folate metabolism has been implicated in neuronal alterations in the CNS (Meethal et al., 2013), although an effect on other cells of the CNS such as astrocytes, seems possible (Shao et al., 2019). Not much is known about methotrexate’s effects on the PNS.

#### Cyclophosphamide

Cyclophosphamide (CP) belongs to the group of alkylating drugs. It is administered as a prodrug, which is metabolized in the liver to produce the active drug. Thus, the hepatic cytochrome P-450 enzymes convert CP into hydroxy-cyclophosphamide, which is then metabolized to aldophosphamide. Aldophosphamide is cleaved to phosphoramide mustard and acrolein, and it is phosphoramide which induces the antitumoral effect. Similar to platinum compounds, the mechanism of action by which CP induces antiproliferative effect is by creating crosslinks between the two strands of DNA at the guanine N-7 position, therefore preventing cell replication and repair which eventually leads to cell death (Singh et al., 2018; Ogino and Tadi, 2021). Although it may cause CNS neurotoxicity (see below), to our knowledge, its potential peripheral neurotoxic effects have not been described.

## Central Neurotoxicity: Chemobrain

Chemotherapy might lead to the development of severe CNS side effects including seizures, strokes, syndrome of inappropriate antidiuretic hormone (SIADH) secretion, visual, hearing, and taste loss as well as memory deficiencies. Taken together, these changes can result in dementia, problems with consciousness, and cognitive disorders known as “chemobrain” (Newton, 2012; Magge and DeAngelis, 2015; Hoeffner, 2016; Taillibert et al., 2016). The term “chemobrain” or “chemofog” was coined for chemotherapy-induced cognitive impairment in the 2000s (Wefel et al., 2004). Cognitive complaints are common among cancer patients during and after chemotherapeutic treatment. The growing body of evidence shows that chemotherapy is associated with short- and long-term mood alterations and cognitive deficits, characterized by disruptions in learning, short-term and working memory, impaired attention and concentration, information processing speed, and executive functions (e.g., multi-tasking, decision-making, language) (Dietrich et al., 2015; Bompaire et al., 2017). Chemofog is also related to anxiety, depression, fatigue and insomnia, and overall health-related decline. In turn, these changes may significantly affect activities of daily living, including autonomy, return to work, social relationships, and self-confidence (Minton and Stone, 2012; Pullens et al., 2013; Dhillon et al., 2018).

Cognitive changes are rather subtle, subjective and difficult to measure, therefore they are often undetected or underestimated by patients and clinicians (Horowitz et al., 2018). Thus, it is difficult to estimate the percentage of cancer survivors with chemobrain and a wide range between 17 and 75% has been estimated (Hermelink et al., 2010; Wefel and Schagen, 2012). Therefore, the International Cancer and Cognition Task Force made recommendations to unify studies on cognitive functions in patients with cancer, based on neuropsychological tests and clinical data. Recommended tests measure learning and memory, processing speed, and executive function based on findings of the cognitive effects of chemotherapy on the frontal cortex (Wefel et al., 2011). Chemobrain is most noticeable in the population of patients with breast cancer, with a frequency reaching 80% (Oberste et al., 2018), but also in lung cancer (Simo et al., 2018), CNS malignancies (Fliessbach et al., 2005; Schagen et al., 2014), testicular cancer (Schagen et al., 2008; Wefel et al., 2014) and hematologic malignancies (Scherwath et al., 2013).

Although it cannot be discarded that other treatments received by the cancer patients (opiates, neuroleptics) and other confounders, like inadequate nutritional status, may contribute to the problem, preclinical *in vitro* and *in vivo* studies do support that chemotherapy causes brain neurotoxicity (Rzeski et al., 2004; Matsos and Johnston, 2019; Boullon et al., 2021; Bagues et al., 2022). Indeed, currently, chemofog is hypothesized to be the result of neuronal injury with abnormal brain remodeling. It might be related to neuroinflammation, neuroendocrine changes, alterations in the BBB that allow increased access of cytotoxic agents and pro-inflammatory cytokines to neurons and supportive glial (astrocytes and microglia) cells as well as secondary activation of glial cells and myelin-producing (oligodendrocyte lineage) cell defects. Of importance, these cognitive deficits appear following treatment with various chemotherapeutic agents, independently of their mode of actions (Rzeski et al., 2004; Dietrich et al., 2006; Monje and Dietrich, 2012; Wefel and Schagen, 2012; Wigmore, 2013; Shi et al., 2019b; Gutmann, 2019; Lange et al., 2019; Walczak and Janowski, 2019; Nguyen and Ehrlich, 2020). Below, we describe the involvement of the different chemotherapeutic drugs and various biological mechanisms in the development of chemofog in detail. Table 3 summarizes these findings.

**TABLE 3:** Involvement of chemotherapeutic agents on chemobrain development basing on their molecular activity.

Chemotherapeutic agent	Mechanism	Type of the study	Reference
Alkylating agents			
Cisplatin	Impairment in neurogenesis	*In vitro, in vivo*	Dietrich et al. (2006), Lomeli et al. (2017)
	Impairment in neural network dynamics	*In vivo*	Andres et al. (2014), Ma et al. (2018)
	Impairment of LTP	*In vivo*	Mu et al. (2015)
	Stimulation of neuroinflammation	Clinical studies	Brandolini et al. (2019)
	Abnormal exocytic neurotransmitters secretion	*In vitro*	Mohammadi et al. (2018)
Carmustine	Toxicity for NPCs	*In vitro, in vivo*	Dietrich et al. (2006)
	Limited self-renewal of OPCs	*In vitro, in silico*	Hyrien et al. (2010)
	Stimulation of neuroinflammation		Gaman et al. (2016)
	Induction of oxidative stress		
Cyclophosphamide	Inhibition of new cell production in the hippocampus	*In vivo*	Christie et al. (2012)
	Reduction of spinal and dendritic complexity	*In vivo*	Acharya et al. (2015), Kang et al. (2018)
	Stimulation of neuroinflammation	*In vivo*	Shi et al. (2019b), Gaman et al. (2016)
	Disruption of microglia function	*In vivo*	Shi et al. (2019a)
thioTEPA	Decreased number of NPCs, immature and mature neurons		Nguyen and Ehrlich, (2020)
Oxaliplatin	BBB breakdown	*In vivo*	Branca et al. (2018)
	Stimulation of neuroinflammation	*In vivo*	Brandolini et al. (2019)
Carboplatin	Impairment in neurotransmitter release	*In vivo*	Kaplan et al. (2016), Field et al. (2018)
Microtubule destabilizing drugs			
Vincristine, Vinblastine	Impairment in neuronal polarization		Sordillo and Sordillo, (2020)
Vincristine	Stimulation of neuroinflammation	Clinical studies	Brandolini et al. (2019)
Microtubule stabilizing drugs			
Docetaxel	Decreased number of NPCs, immature and mature neurons		Nguyen and Ehrlich, (2020)
	Impairment in neuronal stabilization		Mihlon et al. (2010), Sordillo and Sordillo (2020)
	Stimulation of neuroinflammation	*In vivo*	Groves et al. (2017), Shi et al. (2019b)
	Disruption of microglia, astrocytes function	*In vivo*	Shi et al. (2019a), Fardell et al. (2014)
Paclitaxel	Stimulation of neuroinflammation	*In vivo*	Brandolini et al. (2019)
	Neuronal damage	*In vivo*	Wardill et al. (2016b)
Antimetabolites			
5-Fluororuracil	Decreased number of NPCs, immature and mature neurons, inhibition of cell production in the hippocampus	*In vivo*	Nguyen and Ehrlich (2020), Han et al. (2008), Monje and Dietrich (2012)
	Impairment in neural network dynamics	*In vivo*	Groves et al. (2017)
	Decreased myelination	*In vivo*	Argyriou et al. (2011), Han et al. (2008)
	Decreased BDNF production	*In vivo*	Mustafa et al. (2008)
	Stimulation of neuroinflammation	*In vivo*	Groves et al. (2017), Shi et al. (2019b)
	Apoptosis and neuronal damage	*In vivo*	Wardill et al. (2016b)
	Reduction in dopamine secretion	*In vivo*	Jarmolowicz et al. (2019)
Methotrexate	Decreased number of NPCs, immature and mature neurons	*In vivo*	Nguyen and Ehrlich, (2020)
	Dysregulation of microglia, astrocytes, and oligodendrocytes	*In vivo*	Seigers et al. (2010), Geraghty et al. (2019), Gibson et al. (2019)
	Stimulation of neuroinflammation	Clinical studies	Brandolini et al. (2019), Yang et al. (2012), Gaman et al. (2016)
		*In vivo*	
	Polymorphism of ADORA2A	Clinical studies	Tsujimoto et al. (2016)
Antibiotics			
Doxorubicin	Decreased number of NPCs, immature and mature neurons		Nguyen and Ehrlich, (2020)
	Inhibition of cell production in the hippocampus		Monje and Dietrich, (2012)
	Reduction of spinal and dendritic complexity	*In vivo*	Kang et al. (2018)
	Impairment of LTP	*In vivo*	Alhowail et al. (2019), Shaker et al. (2021)
	Decreased BDNF production	*In vivo*	Park et al. (2018)
	Stimulation of neuroinflammation	*In vivo*	Shi et al. (2019b)
	Disruption of microglia function	*In vivo*	Shi et al. (2019a)
	Impaired neurotransmitter release	*In vivo*	Thomas et al. (2017), El-Agamy et al. (2018), Keeney et al. (2018)
	Induction of oxidative stress in the brain	*In vitro*	Gaman et al. (2016), Alhowail et al. (2019), Shaker et al. (2021), Keeney et al. (2018), El-Agamy et al. (2018), Joshi et al. (2007)

ADORA2A, Adenosine A2A receptor; BBB, Blood-Brain Barrier; BDNF, Brain-derived neurotrophic factor; LTP, Long-term potentiation; NPCs, Neural precursor cells; OPCs, Oligodendrocyte precursor cells.

### Impairment in Neurogenesis and Neural Network Dynamics

Reduced neurogenesis is the most commonly studied mechanism for chemobrain. Adult neurogenesis occurs primarily in niche regions: the subgranular zone (SGZ) of the dentate gyrus of the hippocampus, the subventricular zone (SVZ) lining the lateral ventricles (Ming and Song, 2011), and the striatum (Ernst et al., 2014). In the SGZ, neural precursor cells (NPCs) undergo self-renewal or give rise to immature cells that can differentiate into neurons and glial cells (Choi and Goldstein, 2018). Chemotherapeutic drugs are designed to selectively target rapidly proliferating cells, but in most neurons this process is stopped. Neurogenic regions of the brain, such as the hippocampus, are the most vulnerable for anti-mitotic therapies (Wigmore, 2013). Chemotherapeutic agents can lead to extensive DNA damage in post-mitotic neurons, inducing their death (Hoeijmakers, 2009; Maynard et al., 2015). Lower doses of chemotherapy, similar to the levels of chemotherapeutic drugs found in the brain after systemic administration, may cause cellular senescence. Senescent cells are non-proliferating but remain metabolically active. They exhibit so called senescence-associated secretory phenotype (SASP), which consists of growth factors, inflammatory and proangiogenic agents, chemokines, metalloproteinases, and many others, that may affect their environment. Of note, some recent studies suggest that senescent cells can re-enter the cell cycle and carry abnormal divisions (Sundaram et al., 2004; Campisi and d'Adda di Fagagna, 2007; Sliwinska et al., 2009; Gewirtz, 2014; Erenpreisa et al., 2015; Niu et al., 2017; Was et al., 2017; Mirzayans et al., 2018; Was et al., 2018; Sikora et al., 2021). The development of neuronal senescence can be also linked to oxidative damage, as it was observed in rodents treated with DCTX (Moruno-Manchon et al., 2016). In addition, the post-mitotic brain shows many alterations of DNA repair mechanisms, increasing the chance of apoptosis or senescence induction (Maynard et al., 2015). Therefore, the accumulation of DNA damage caused by chemotherapy can accelerate neuronal dysfunction and death. Intraperitoneal (IP) or intravenous (IV) injections of various drugs, ranging from MTX, 5-FU, CP, doxorubicin (DOX), DCTX, PCTX, cisplatin to thioTEPA were shown to increase the impairment of memory and decreased numbers of NPCs, immature or mature neurons (Nguyen and Ehrlich, 2020). In accordance, several studies found a reduction in the number of NPCs in the brains of cancer survivors (Seigers et al., 2008; El Beltagy et al., 2010; Briones and Woods, 2011; Nokia et al., 2012). Carmustine, cisplatin, and cytosine arabinoside (cytarabine) were reported to be more toxic for the progenitor cells of the CNS than they are for multiple cancer cell lines. In preclinical studies, administration of these chemotherapeutic agents was associated with increased cell death and reduced cell proliferation in the SVZ, in the dentate gyrus of the hippocampus and the *corpus callosum* of the CNS (Dietrich et al., 2006). In turn, 5-FU was demonstrated to be toxic for CNS progenitor cells *in vitro* and *in vivo*. Systemic treatment with 5-FU was shown to cause CNS impairment with severe damage to white-matter tracts of treated animals (Han et al., 2008). CP, DOX, and 5-FU prevented the production of new cells in the hippocampus, which is crucial for brain plasticity and neural repair. Therefore, suppression of neurogenesis was directly related to chronic neurotoxic effects, including hippocampus-dependent cognitive functions (Christie et al., 2012; Monje and Dietrich, 2012; Lomeli et al., 2017).

Most neurons are highly polarized cells with complex morphology (Barnes and Polleux, 2009). The microtubule network is critical for regulating their polarization, migration, cell proliferation, and cargo transport (Dubey et al., 2015). Spines and dendrites regulate the synaptic plasticity essential for learning, memory, and executive functions (Forrest et al., 2018) and they remain dynamic even in mature, non-dividing neurons (Forrest et al., 2018). Several studies have shown a reduction in dendritic and spinal complexity in rodents following the administration of chemotherapeutic agents such as cisplatin (Andres et al., 2014), 5-FU (Groves et al., 2017), DOX, and CP (Acharya et al., 2015; Kang et al., 2018). High cisplatin levels led to the rapid loss of synapses and dendritic disintegration, and neuronal, but not NPCs apoptosis. In accordance, *in vivo* administration of cisplatin reduced dendritic branches and decreased spine density in CA1 and CA3 hippocampal neurons (Andres et al., 2014). Interestingly, short-term daily treatment with the histone deacetylase 6 (HDAC6) inhibitor was demonstrated to reverse behavioral, structural, and functional deficits induced by cisplatin in a mouse model (Ma et al., 2018). Drugs that target the microtubule network directly through hyperstabilizing (PCTX, DCTX, and ixabepilone) or destabilizing (vincristine and vinblastine) it, may lead to chemobrain as well (Mihlon et al., 2010). Abnormalities in microtubules were reported to cause cognition and memory problems, and damage to microtubules correlated with numerous neurological diseases (Sordillo and Sordillo, 2020). Accordingly, PCTX, the microtubule-stabilizing agent, was demonstrated to cause impaired memory acquisition in rodent models. Chemotherapeutics-treated animals behaved poorly in the Morris water maze (MWM) test comparing to counterparts, and microtubule dynamics in their brain extracts were significantly reduced (Atarod et al., 2015; You et al., 2018).

Chemotherapeutics do not only act by the induction of neurodegeneration, but they can also play a crucial role in disturbing neuronal signaling and altering long-term potentiation (LTP). LTP plays an essential role in synaptic mediation of learning acquisition and memory consolidation, whose disturbances are characteristic of chemobrain. Those changes are most likely related to the ability of chemotherapeutics to interact off-target with: N-methyl-D-aspartate receptor (NMDA-R), α-amino-3-hydroxy-5-methyl-4-isoxazole propionic acid receptor (AMPA-R), protein kinase A (PKA), extracellular signal-regulated kinase (ERK2), and Ca^2+^/calmodulin-dependent protein kinase II (CaMKII), which are major regulators of LTP. The effect on LTP was pointed as possible for several drugs, including dactinomycin and irinotecan in *in silico* simulation (Fahim et al., 2019). However, impairment of LTP was also observed in rodent models due to cisplatin and DOX treatment (Mu et al., 2015; Alhowail et al., 2019). Cisplatin reduced spontaneous exploratory activity, recent and remote memory retrieval, induced decision-making, which correlated with impairment in LTP-like synaptic plasticity (Mu et al., 2015). In turn, DOX led to reduced LTP in the hippocampal region as well as a decrease in postsynaptic potentials in the rat brains (Alhowail et al., 2019). Synaptic plasticity and normal cognitive function strongly depend also on Sirtuin I (SIRT1), which is a histone III deacetylase. In mice, SIRT1 is localized specifically in neurons, but not in glial cells. The lack of this protein resulted in defective neuron plasticity and disturbances in LTP-related processes, which led to impairment of cognition (Michan et al., 2010). It was observed that expression of SIRT1 decreased upon treatment with DOX (Shaker et al., 2021).

Altogether, different chemotherapeutic drugs may cause chemobrain by inducing neurons’ senescence or death, alter their polarization and ability to form functional networks.

### Downregulated Secretion of Neurotransmitters

Most neurological agents act through modulating neurotransmitters. Variants of catechol-O-methyltransferase (COMT) were correlated with differential risks of developing chemobrain in cancer survivors. COMT catalyzes the O-methylation of the catecholamines: dopamine (DA), epinephrine, and norepinephrine (NE) (Sheldrick et al., 2008). *In vitro* experiments on PC12 cells, which are a type of catecholaminergic cells that synthesize, store and release NE and DA, revealed that cisplatin may affect cell membrane lipids, particularly phosphatidylcholine and cholesterol, leading to the abnormal exocytotic release of neurotransmitters (Mohammadi et al., 2018). In turn, carboplatin affected dopamine and serotonin (5-HT) release and uptake in rat brains, which was related to cognitive and mood impairments (Kaplan et al., 2016). Carboplatin was demonstrated to impair dopamine release and uptake in the zebrafish model of chemobrain-related studies (Field et al., 2018). Similarly, a reduction in DA release in the striatum following injections of 5-FU was shown (Jarmolowicz et al., 2019). Treatment with DOX impaired glutamate neurotransmission in the mouse frontal cortex and hippocampus. Especially, glutamate clearance was reduced by about 50% in the frontal cortex and dentate gyrus (Thomas et al., 2017). It might be related to the reduced glutamate transporter expression (Thomas et al., 2017) and impaired exocytosis (Kaplan et al., 2016). Additionally, elevated acetylcholine esterase activity was reported in the hippocampus of rats treated with DOX (El-Agamy et al., 2018). In accordance, a reduction in choline content, the precursor for acetylcholine, was also demonstrated after DOX treatment (Keeney et al., 2018), suggesting that inhibited cholinergic activity may be a risk factor in chemobrain.

Altogether, various chemotherapeutic drugs may cause chemobrain by reducing neurotransmitters’ production and disturbing effective communication between neurons.

### Reduced Gliogenesis and Hyperactivation of Microglia and Astrocytes

Non-neuronal cells, including astrocytes, oligodendrocytes, and microglia, play important roles in maintaining the health and normal functions of neurons (Jessen, 2004; Walczak and Janowski, 2019). Reduced gliogenesis in the SVZ and SGZ can impair memory encoding and consolidation (Fields et al., 2014). Proper axonal myelination is crucial for fast conduction speed and enhanced cognitive processing (Bendlin et al., 2010; Lu et al., 2011). Generation of new oligodendrocytes is also required for complex motor learning (McKenzie et al., 2014) and spatial memory consolidation (Steadman et al., 2020). Glial cells, as fast-dividing cells, are very vulnerable to chemotherapeutic agents. Carmustine, cisplatin, and cytosine arabinoside (cytarabine), when injected systemically in mice, were demonstrated to inhibit proliferation and induced cell death of progenitor cells and oligodendrocytes throughout the CNS (Dietrich et al., 2006). Several other studies demonstrated that PDGFRa+/Olig2+ oligodendrocyte precursor cells (OPCs) and non-dividing mature oligodendrocytes are especially sensitive to chemotherapy as compared to neurons and astrocytes (Dietrich et al., 2006; Han et al., 2008; Hyrien et al., 2010; Gutmann, 2019). Persistent depletion of OPCs in the subcortical white matter tracts was shown after MTX treatment both in humans and mice (Gibson et al., 2019). Moreover, chemotherapeutics also altered OPCs differentiation and consequently proper myelination (Hyrien et al., 2010; Gibson et al., 2019) and were associated with numerous behavioral deficits, including decreased forepaw swing speed, increased anxiety (attention system dysfunction), and persistent cognitive defects in mouse models (Gibson et al., 2019). Mathematical and experimental analysis showed that transient exposures to carmustine lengthened the cell cycle of OPCs and accelerated their time of differentiation. In accordance, their ability to self-renew was significantly limited (Hyrien et al., 2010). Of importance, imaging studies on human cancer survivors reveal a reduction in several white matter tracts, suggesting reduced myelination (Deprez et al., 2011; Deprez et al., 2012; Chen et al., 2020). One of the drugs, which can cross BBB and act directly on cells of CNS, causing decreased myelination, is 5-FU (Argyriou et al., 2011). Systemic administration of 5-FU led to acute CNS injury and damage of myelinated tracts of the CNS that was correlated with changes in transcriptional regulation of oligodendrocytes and an increased myelin pathology (Han et al., 2008). Furthermore, damage to neurons or glial cells can activate microglia and astrocytes, leading to increased production of inflammatory cytokines and the development of chronic neuroinflammation. Astrocyte activation was demonstrated after DCTX injection (Fardell et al., 2012), and microglial activation was shown after CP treatment (Christie et al., 2012). Additionally, hyperactivation of microglia may contribute to long-term cognitive deficits as shown for MTX (Seigers et al., 2010). Astrocytes and oligodendrocytes can be also dysregulated following MTX treatment (Geraghty et al., 2019; Gibson et al., 2019). MTX treatment was reported to cause persistent activation of microglia and subsequent astrocyte activation, further leading to exhaustion of OPCs, reduced myelination, and decreased cortical brain-derived neurotrophic factor (BDNF) levels. In turn, pharmacological depletion of microglia normalized oligodendroglial lineage dynamics, myelin microstructure, and cognitive and behavioral deficits after MTX chemotherapy (Gibson et al., 2019). Brain blood vessels-derived vascular endothelial growth factor (VEGF) and BDNF were demonstrated to play a crucial role in the survival and differentiation of NPCs (Schanzer et al., 2004; Goldman and Chen, 2011).

Altogether, different chemotherapeutics may lead to chemofog by reducing gliogenesis and causing activation of microglia and astrocytes, resulting in neuroinflammation.

### Neuro-Inflammation and Breakdown of Blood-Brain Barrier

Chronic neuroinflammation might be responsible for maintaining long-term cognitive dysfunctions (Glass et al., 2010; Michaud et al., 2013). As mentioned above (*Peripheral Neuropathy: Neuropathic Pain*), most chemotherapeutic agents administered systemically do not cross the intact BBB. Cytokines in the brain are mainly derived from microglia, astrocytes, oligodendrocytes, and neurons, rather than peripheral sources (Kronfol and Remick, 2000). Activated microglia and astrocytes can produce cytokines, e.g., IL-1β and TNF-α directly in CNS. Peripherally released cytokines can also access the brain and compromise the protective BBB, thereby enabling the entrance of more cytokines and chemotherapeutic drugs (Ren et al., 2017) and increased peripheral cytokines are observed in cancer survivors (Wang et al., 2015). Pro-inflammatory cytokines may affect tight junctions and thus the integrity of the BBB. This is important, when considering BBB breakdown in cancer patients, as there is a number of sources of pro-inflammatory cytokines, derived from the tumor and the effects of chemotherapy on other organs (Wardill et al., 2016b). Cytokines enter the brain by receptor mediated-endocytosis or passive diffusion through the leaky regions of BBB, resulting in glial activation and local inflammatory reaction in the brain (Banks and Erickson, 2010; Wardill et al., 2016b). In a rat brain endothelial cell line (RBE4) oxaliplatin induced the disassembly of the tight junctions and brain dysfunction (Branca et al., 2018). 5-FU and a combination of DCTX, DOX, and CP led to cytokine dysregulation and disruptions in neuroplasticity, which correlated with behavioral and imaging alterations in a mouse model. In turn, these changes were associated with serum and brain levels of pro-inflammatory cytokines, IL-6 and TNF-α. On the other hand, levels of anti-inflammatory cytokines IL-4 and IL-10 in serum and brain were reduced in most animals (Groves et al., 2017; Shi et al., 2019b). Elevated IL-6 and TNF-α that were detected in chemotherapy-treated breast cancer survivors correlated with persistent hippocampal structural changes and reduced verbal memory performance (Collado-Hidalgo et al., 2006; Bower et al., 2007). BBB permeability, astrocytic activation, and peripheral production of IL-1β, IL-6 and TNF-α were demonstrated to increase also after treatment with irinotecan (Wardill et al., 2016a).

It is suspected that overproduction of pro-inflammatory proteins which can cross BBB, such as TNF-α, can stimulate microglial cells to further produce inflammatory cytokines, which promote brain damage (Tangpong et al., 2007; Tacar et al., 2013). It was observed that upon treatment with a combination of DCTX, DOX and CP, microglia cells drastically changed in morphology. They became round and ameboid-like in shape with large perinuclear cytoplasm including the dense and heterochromatic nucleus (Shi et al., 2019a). It was shown that prolonged activation of microglia can act as a mechanism leading to neurodegenerative disorders and can inhibit neurogenesis due to the impairment in neuronal stem cells (NSCs) located in the hippocampus (Dheen et al., 2007; Smith et al., 2012).

At least partly due to BBB breakdown, some chemotherapeutic agents, such as cisplatin, carmustine, PCTX, and 5-FU, were also detected in the brains of rodents or primates after IV injection, leading to apoptosis and neuronal damage associated with neurological dysfunction (Wardill et al., 2016b). DCTX led to cognitive impairment of mice in post-treatment behavioral tests, and this was correlated with substantial amounts of DCTX in the brain and astrocyte activation. DCTX treatment did not significantly elevate the rate of apoptosis within the CNS, but increased autophagy was detected (Fardell et al., 2014). The neurotoxic effect of DCTX in neurons might be modulated by impairment of autophagy and damage of lysosomes, which was demonstrated in both *in vitro* and *in vivo* studies (Moruno-Manchon et al., 2016). The anti-autophagic capacity of DCTX was marked by the downregulation of PTEN-induced kinase 1 (PINK1) and Parkin expression (Wahdan et al., 2020). MTX-treated breast tumor-bearing mice exhibited significant depressive-like behaviors and cognitive impairment. At the same time, these mice showed reduced numbers of NPCs in the hippocampal dentate gyrus and augmented pro-inflammatory enzymes, including iNOS and COX-2 (Yang et al., 2012). Patients undergoing chemotherapy with taxanes or anthracycline-containing regimens for breast, ovarian cancer, and Hodgkin’s disease had statistically significant increases in interferon (IFN)-α, IL-1β, IL-6, IL-8, IL-10, and monocyte chemoattractant protein 1 (MCP-1) (Wang et al., 2015). Cytokine change may induce alterations in neurotransmitter systems and neuronal integrity by inducing excitotoxic glutamate receptor-mediated damage, influencing monoaminergic systems [serotonin (5-HT), DA, and NE], γ-aminobutyric acid (GABA), acetylcholine, neuropeptides, and BDNF, which are directly associated with cognitive function and neurodegenerative processes (Wang et al., 2015).

BBB breakdown might be also a result of cerebral blood pressure autoregulation failure (Stone and DeAngelis, 2016). One of the theories explaining the development of this dysfunction claims that failure of blood pressure autoregulation is induced by endothelial dysfunction. It may result from circulating toxins, including chemotherapeutics (Fischer and Schmutzhard, 2017): vincristine, gemcitabine, cytarabine, and cyclosporine (Peddi et al., 2014; Hoeffner, 2016; Stone and DeAngelis, 2016).

Altogether, different chemotherapeutic agents may cause chemobrain by disturbing the BBB and inducing inflammation in both the CNS and the periphery.

### Overproduction of Reactive Oxygen Species

Chemobrain seems to be accompanied by ROS and reactive nitrogen species (RNS) production, that cumulatively increase oxidative stress (Chen et al., 2007). The post-mitotic brain consumes ∼20% of glucose-derived energy, leading to high production of ROS (Patel, 2016). Additionally, the activated glial cells were demonstrated to upregulate nitric oxide synthase (NOS) and RNS production (Tangpong et al., 2006). Finally, a majority of cancer survivors are older, which may additionally increase the level of ROS and senescence phenotype (Miller et al., 2019). ROS are known as DNA-damaging and senescence-inducing agents. The overproduction of ROS and RNS have been reported as the most frequent cause of DNA damage in neuronal cells (Abner and McKinnon, 2004) and a direct cause of a cognitive decline in cancer patients (Nadin et al., 2006).

It was observed that oxidative stress in the brain can be induced by DOX, carmustine, MTX, and CP (Gaman et al., 2016). DOX is known to generate intracellular ROS. In *in vitro* studies, it was shown that DOX can significantly induce mitochondrial stress in hippocampus-derived cells (Alhowail et al., 2019). Even though DOX does not cross the BBB, it can modulate ROS production indirectly in the CNS. It was responsible for the oxidization of plasma apolipoprotein A1, that in turn promoted macrophage TNF-α production, which significantly induced oxidative stress in the brain (Aluise et al., 2011). TNF-α overproduction correlated with increased levels of glutathione-S-transferase as well as glutathione peroxidase and reductase (Joshi et al., 2010). It also decreased mitochondrial respiration as a result of increased p53 and apoptosis-associated proteins (Tangpong et al., 2006). Similar to DOX, treatment with carmustine, MTX and CP also generated increased levels of TNF-α in plasma (Gaman et al., 2016).

Oxidative stress in the hippocampus induced by DOX, followed by neurodegeneration, correlated with decreased expression of SIRT1 protein. SIRT1 plays a crucial role in the regulation of ROS production and controls the expression of proteins responsible for oxidative stress protection (Olmos et al., 2013; Ren Z. et al., 2019). Berberine (BER), the natural alkaloid, decreased cognitive impairment and protected against DOX toxicity in a rodent model. The mechanism of BER was most likely based on restoration of the metabolic balance controlled by SIRT1, as its expression increased in response to BER treatment (Shaker et al., 2021). Moreover, DOX administration was shown to cause a significant decrease in choline-containing compounds and activities of the phospholipase enzymes in the hippocampus in mice. DOX administration was shown to correlate with elevated apoptosis of hippocampal neurons, diminished antioxidant glutathione (GSH) levels, reduced activity of catalase, and elevated level of the lipid peroxidation products in a rat model (El-Agamy et al., 2018). In turn, γ-glutamyl cysteine ethyl ester (GCEE), a precursor of the antioxidant GSH, restored GSH level and GSH transferase activity and reduced the levels of oxidative stress markers of protein oxidation and lipid peroxidation in DOX-treated mice brains (Joshi et al., 2007).

Finally, ROS can compromise the BBB by triggering several pathways (Pun et al., 2009). Of importance, mice co-treated with the natural antioxidant, resveratrol demonstrated a reversion of harmful effects of chemotherapy-induced cytokine dysregulation and disruptions in neuroplasticity (Shi et al., 2018).

Altogether, different chemotherapeutic agents may activate chemofog by inducing oxidative stress, which accelerates neuronal degeneration, inflammatory cytokine overproduction and a leak of the BBB.

### Genetic Alterations

Risk factors responsible for the development of cognitive impairments are still not clear. However, there is some evidence indicating that there are some genetic alterations that might increase the possibility of chemobrain occurrence. Some studies indicate that the rate of MTX-mediated toxicity might be regulated by adenosine, whose release is promoted by this drug (Cronstein et al., 1993). Adenosine regulates CNS by interactions with the adenosine A2A receptor (ADORA2A). Polymorphisms in the gene encoding this protein were associated with an increased risk of encephalopathy and the development of cognitive disorders after this type of treatment (Tsujimoto et al., 2016). It is suggested that patients with higher levels of DNA impairments have a much higher risk of developing cognition-related problems. In accordance, genetic polymorphisms in DNA repair genes, including 8-Oxoguanine glycosylase (OGG1)*,* APEX1, XRCC1*,* ERCC1*,* XPC*,* XPD*,* XPF*,* BRCA2*,* XRCC3, and MDR1 (multi-drug resistance -1) are considered important factors to influence the degree of chemotherapy-associated CNS damage, especially those associated with oxidative stress (Goode et al., 2002; Kerb, 2006; Ahles and Saykin, 2007; Ren X. et al., 2019). Moreover, disturbances in the expression of MDR1 and closely associated multidrug resistance-associated protein-1 (MRP1) can promote BBB damage, which directly influences the level of chemotherapeutics in the brain. The presence of those polymorphisms allows toxic drugs to cross BBB and therefore increase their deleterious activity on neuronal cells (Ren X. et al., 2019).

Chemofog risk increases when polymorphisms occur in the genes associated with neuron growth and their functions. For example, it was observed that BDNF Val66Met mutation increased the incidence of chemobrain. BDNF genetic alterations were demonstrated to play a role in neuroinflammation-linked cognitive depression in breast cancer survivors (Dooley et al., 2016; Ng et al., 2017) and their poorer performance on verbal fluency and multitasking test (Ng et al., 2016). In turn, polymorphism of COMT, the main modulator of neurotransmitter (e.g., DA, epinephrine, and NE) degradation, was shown to affect their bioavailability (Miskowiak et al., 2017). It has been correlated with cognitive impairment (e.g., decreased performance in attention, motor speed, and verbal fluency) in lymphocytic leukemia (Cole et al., 2015) and adult breast cancer survivors (Small et al., 2011).

Apolipoprotein E (APOE) is a glycoprotein responsible for lipid transport in the nervous system. It also plays a crucial role in the maintenance of BBB, as well as in processes of oxidative stress, inflammation, and neuronal health. APOE can produce three alleles, but only the presence of *APOE4* is considered as one of the possible risk factors for chemobrain development (Fernandez et al., 2020). Cognitive impairment might occur regardless of used therapy, as it was observed in the cohort study on lymphoma and breast cancer survivors. It was demonstrated that patients bearing the *APOE4* allele showed lower performance in visual memory and spatial ability (Ahles et al., 2003). The correlation of *APOE4* and cognitive impairment was further confirmed in other studies conducted on breast cancer survivors in different age groups (Ahles et al., 2014; Mandelblatt et al., 2018). *In vivo* studies revealed that the presence of *APOE4* in mice resulted in a slight decrease in the volume of the frontal cortex and grey matter. Observations made on the role of *APOE4* in chemobrain development in cancer survivors were confirmed by controlled studies in rodents (Speidell et al., 2019; Demby et al., 2020). The presence of *APOE4* correlated with breakage of BBB due to impairment of tight-junctions (Nishitsuji et al., 2011) as well as activation of pro-inflammatory cytokine secretion by pluripotent stem cell-derived microglia *in vitro* (Lin et al., 2018).

Altogether, different genetic alterations may increase the chance of chemobrain by affecting neuronal morphology and functions.

## Enteric Neurotoxicity

Diarrhea, constipation, oral mucositis, nausea, and vomiting are common gastrointestinal side effects of chemotherapeutic medications experienced by 80–90% of patients (Keefe et al., 2014; McQuade et al., 2014; Numico et al., 2015; McQuade et al., 2016a; Sommariva et al., 2016; Escalante et al., 2017; Dahlgren et al., 2021). As a result of these side effects, patients commonly develop malnutrition and dehydration. Early death rates of up to 5% associated with chemotherapy are primarily due to gastrointestinal toxicity (Rothenberg et al., 2001). The gastrointestinal side effects often limit the dose of chemotherapy reducing the efficacy of anti-cancer treatment, leading to poor survival, and having a severe impact on a patient’s quality of life (Arbuckle et al., 2000; Sharma et al., 2005; Mitchell, 2006; McQuade et al., 2017a; Sougiannis et al., 2021). Chronic post-treatment diarrhea can persist for over 10 years after the end of treatment in 13–50% of long-term cancer survivors (Denlinger and Barsevick, 2009; Buccafusca et al., 2019). Most drugs used clinically to alleviate gastrointestinal side effects of chemotherapy have adverse effects themselves and often have limited efficacy (McQuade et al., 2016a; Koth and Kolesar, 2017).

The traditional view is that gastrointestinal side effects of anti-cancer drugs are due to mucosal damage (Keefe et al., 2004; Van Sebille et al., 2015; Dahlgren et al., 2021). However, while mucosal damage is undoubtedly significant for the acute symptoms associated with chemotherapy (Pulito et al., 2020; Sougiannis et al., 2021), the persistence of gastrointestinal symptoms long after treatment is completed suggests that there is long-term damage to the gastrointestinal innervation (Thorpe et al., 2013; Escalante et al., 2017; McQuade et al., 2020).

The enteric nervous system (ENS) embedded into the wall of the gastrointestinal tract controls its functions; most of the enteric neurons locate within the enteric ganglia organized into two major plexi, myenteric plexus that controls intestinal motility and submucosal plexus that controls intestinal secretion and vasodilation (Bornstein et al., 2012; Furness, 2012; Bon-Frauches and Boesmans, 2020; Fleming et al., 2020; Brehmer, 2021). Despite mounting evidence for chemotherapy-induced enteric neuropathy, research in this area is scarce. As described below, recent studies demonstrate that enteric neuronal degeneration and dysfunction are emerging key players in gastrointestinal disorders induced by chemotherapy. Several chemotherapeutic agents currently used in the clinic for anti-cancer treatment have a profound impact on the ENS, and mechanisms underlying enteric neuropathy caused by different classes of chemotherapeutic drugs vary to a significant extent.

### Mechanisms Involved in Chemotherapy-Induced Enteric Neuropathy

Damage and death of enteric neurons, severe axonal damage, changes in glial cells, neuropeptides, and in neuromuscular transmission, as well as altered molecular expression, persisted long after the cessation of chemotherapy when mucosal damage has subsided in animals treated with cisplatin (Vera et al., 2011; Pini et al., 2016; Uranga et al., 2017; Nardini et al., 2020), oxaliplatin (Wafai et al., 2013; McQuade et al., 2016c; Robinson et al., 2016; Stojanovska et al., 2018b), 5-FU (McQuade et al., 2016b), irinotecan (McQuade et al., 2017b; Thorpe et al., 2020), vincristine (Hobson et al., 1974; López-Gómez et al., 2018; Gao et al., 2021), and adriamycin/doxorubicin (Cheng et al., 1997, 1999). Studies in fresh colon specimens resected from chemotherapy-treated non-obstructive carcinoma patients revealed morphological changes and hyperexcitability of myenteric neurons, and increased numbers of neurons with nuclear translocation of Hu, an mRNA binding protein present in all enteric neurons (Stojanovska et al., 2015; Carbone et al., 2016). Enteric neuropathy associates with long-term gastrointestinal dysfunctions, including changes in gastrointestinal transit, oesophageal, gastric and colonic dysmotility, and clinical symptoms: chronic constipation, chronic diarrhea, lack of weight gain, and pica (equivalent to vomiting in humans) (Cheng et al., 1997, 1999; Vera et al., 2006, 2007; Yamamoto et al., 2007; Cabezos et al., 2008; Cabezos et al., 2010; McQuade et al., 2016b; McQuade et al., 2016c; Pini et al., 2016; Spencer, 2016; Abalo et al., 2017; McQuade et al., 2017b; Vera et al., 2017; López-Gómez et al., 2018; Belsky et al., 2020; Gao et al., 2021).

Mechanisms underlying chemotherapy-induced enteric neuropathy involve oxidative stress, inflammation, and direct toxicity leading to mitochondrial and nuclear damage (Escalante et al., 2017; McQuade et al., 2020). Oxidative stress resulting from an imbalance between ROS/RNS production and the antioxidant defense system is an important factor contributing to gastrointestinal dysfunctions (Kashyap and Farrugia, 2011; Aviello and Knaus, 2017). Increased nitrosylation of protein tyrosines by RNS (e.g., reactive peroxynitrates) has been observed in colonic enteric neurons of FOLFOX (a combination of 5-FU, leukovorin, and oxaliplatin) and 5-FU-treated patients and oxaliplatin-treated mice (Carbone et al., 2016; McQuade et al., 2016c). Augmented soma size of neuronal nitric oxide synthase (nNOS)-immunoreactive myenteric neurons has been found in the colons from FOLFOX and 5-FU-treated patients (Carbone et al., 2016). Higher proportions of nNOS-immunoreactive myenteric and submucosal neurons have been observed in the gastric fundus and colon from cisplatin-treated rats and mice, vincristine-treated rats, as well as oxaliplatin-treated mice (Vera et al., 2011; Wafai et al., 2013; McQuade et al., 2016c; Pini et al., 2016; López-Gómez et al., 2018). Increased expression of iNOS and nNOS, excessive production of superoxide, mitochondrial membrane depolarization, the release of cytochrome *c,* and activation of caspase 3 leading to apoptosis of myenteric and submucosal neurons are prominent after oxaliplatin treatment (McQuade et al., 2016c). These findings provide evidence that oxidative stress is a key player in chemotherapy-induced enteric neuropathy. Co-treatment with neuroprotective agents with strong antioxidant properties, resveratrol, and BGP-15, alleviates enteric neuropathy and gastrointestinal dysfunction induced by oxaliplatin (Donald et al., 2017; McQuade et al., 2018). However, the use of these compounds is limited due to the low bioavailability and activation of multiple pathways, which might produce unwanted results. Therefore, further studies to determine more specific molecular targets for enteric neuroprotection are needed.

Indirect toxicity resulting from chemotherapy-induced inflammation also leads to damage and death of enteric neurons. Chemotherapeutic agents modulate local and systemic immune activity, causing damage to the epithelial barrier, leading to the infiltration of enterotoxins from the lumen into the lamina propria, inducing the recruitment of leukocytes and stimulation of the production and release of soluble mediators such as cytokines and chemokines. Released mediators and neurotoxins induce damage to the nerve fibers projecting to the mucosa and invade enteric ganglia inducing changes in neuroimmune interactions, ENS structure and functions, leading to neuronal damage and death. Mucosal injury, activation of pro-inflammatory immune cells, increased cytokine release leading to ENS remodeling have been observed in animal models after treatments with cisplatin (Nardini et al., 2020), 5-FU (McQuade et al., 2016b; Abalo et al., 2017), irinotecan (McQuade et al., 2017b; Thorpe et al., 2020), and vincristine (López-Gómez et al., 2018; Gao et al., 2021). Chemotherapy induces changes in TLRs and gut microbiota (Stojanovska et al., 2018a). TLRs trigger activation of transcription factors, such as NF-κB and other intracellular mediators (e.g., HMGB1) causing excessive production of pro-inflammatory cytokines leading to neuronal toxicity underlying intestinal dysfunction (Stojanovska et al., 2015). Recent studies demonstrate that glucagon-like peptide-2 exerting trophic and repair functions in the gut can prevent cisplatin-induced mucosal injury, enteric neuropathy and gliopathy and induce remodeling of the ENS (Nardini et al., 2020). Thus, novel strategies targeting enteric neuropathy might be effective in preventing gastrointestinal dysfunction associated with chemotherapy.

Direct damage to the enteric neurons might be caused by chemotherapeutic agents such as platinum-based chemotherapeutics, which might cause DNA damage. Long-term retention of platinum in the tissues and plasma has been observed in patients treated with platinum-based chemotherapeutics 8–75 months after the end of the treatment (Brouwers et al., 2008). In the DRG, treatment with cisplatin and oxaliplatin caused the binding of platinum to neuronal DNA, and the formation of platinum-DNA adducts resulting in neuronal apoptosis (Ta et al., 2006). It is unknown whether neurons are more susceptible to platinum-based chemotherapeutics or their drug clearance capacity is insufficient. This suggests that platinum-based agents and possibly other chemotherapeutics might cause direct enteric neurotoxicity due to their accumulation in the ENS (Stojanovska et al., 2017), which needs to be investigated. Thus, several mechanisms, including oxidative stress, inflammation, and direct toxicity might contribute to chemotherapy-induced ENS damage.

Mechanisms involved in chemotherapy-induced damage to the enteric glial cells have not been elucidated. Moreover, it should be noted that most animal studies use only a single chemotherapeutic treatment and are performed in naïve animals, whereas, in most cases, clinical treatment of cancer patients involves combinations of chemotherapeutic drugs targeting various pathways in order to achieve enhanced anti-cancer efficacy of the treatment. These are all areas warranting further investigation.

## Peripheral Mechanisms in Nausea and Emesis: Unresolved Issues for Exploration

The treatment of cancer may involve the use of chemotherapeutic agents that have a variety of side effects. As shown in Table 4, anti-cancer agents have been classified according to their potential to induce nausea and emesis and are ranked into high-risk anthracyclines and cyclophosphamide (AC), high-risk non-AC, moderate-, low-, and minimal-risk groups (Roila et al., 2016; Hesketh et al., 2020). Most preclinical anti-emetic drug discovery research has focused on mechanisms involving cisplatin, with a view that it is the most emetogenic of the agents used, and if an agent reduces emesis to this anti-cancer drug, it should be also successful to reduce nausea and emesis resulting from the lower-risk treatments (Andrews and Rudd, 2004).

**TABLE 4:** Classification of chemotherapeutic agents according to their emetogenicity.

Risk level	Chemotherapeutic agent	
High (>90%)	Cytotoxic agents	AC combination
		Non-AC agents: carmustine, **cisplatin**, CP (≥1,500 mg/m^2^), dacarbazine, mechlorethamine, streptozotocin
Moderate (30–90%)	Cytotoxic agents	Arsenic trioxide, azacytidine, bendamustine, busulfan, carboplatin, clofarabine, CP (<1,500 mg/m^2^), cytarabine (>1,000 mg/m^2^), daunorubicin, daunorubicin + cytarabine liposome, doxorubicin, epirubicin, idarubicin, ifosfamide, irinotecan, irinotecan liposomal injection, oxaliplatin, romidepsin, temozolomide (*), thioTEPA (#), trabectedin
	Non-cytotoxic agents	Alemtuzumab, fam-trastuzumab deruxtecan-nxki
Low (10–30%)	Cytotoxic agents	5-FU, belinostat, cabazitaxel, cytarabine (up to 1,000 mg/m^2^), decitabine, DCTX, eribulin, etoposide, gemcitabine, ixabepilone, MTX, mitomycin, mitoxantrone, nab-PCTX, nelarabine, PCTX, pegylated liposomal doxorubicin, pemetrexed, topotecan, vinflunine
	Non-cytotoxic agents	Aflibercept, axicabtagene ciloleucel, blinatumomab, bortezomib, brentuximab, carfilzomib, copanlisib, catumaxumab, cetuximab, elotuzumab, enfortumab vedotin-ejfv, gemtuzumab ozogamicin, inotuzumab ozogamicin, moxetumomab pasudotox, necitumumab, panitumumab, tagraxofusp-rzs, tisagenlecleucel, temsirolimus, trastuzumab-emtansine
Minimal (<10%)	Cytotoxic agents	Bleomycin, 2-chlorodeoxyadenosine, cladribine, fludarabine, pixantrone, pralatrexate, vinblastine, vincristine, vinorelbine
	Non-cytotoxic agents	Atezolizumab, avelumab, bevacizumab, cemiplimab, daratumumab, durvalumab, emapalumab, ipilimumab, nivolumab, obinutuzumab, ofatumumab, pembrolizumab, polatuzumab vedotin, ramuciumab, rituximab, trastuzumab

Both cytotoxic and non-cytotoxic (antibodies, protein kinase inhibitors, etc) are classified according to their emetogenic risk after intravenous administration to adults (exceptions: * indicates oral administration; # indicates pediatric patients). Cisplatin is highlighted in bold, since it is the emetogenic drug of reference used in the development of new antiemetics. Adapted from Hesketh et al., 2020. Abbreviations: 5-FU, 5-fluorouracil; AC, anthracycline +cyclophosphamide; CP, cyclophosphamide; DCTX, docetaxel; MTX, methotrexate; PCTX, paclitaxel.

In humans, cisplatin can cause emesis lasting several days via pharmacologically distinct mechanisms. There is an “acute” phase comprising emetic episodes occurring during the first 24 h, which is highly susceptible to 5-HT_3_ receptor antagonists, and a subsequent delayed phase lasting 2–5 days, which is thought to also involve SP and inflammatory mediators. Guidelines recommend a combination of 5-HT_3_ and tachykinin NK_1_ receptor antagonists, plus dexamethasone for acute nausea and emesis to high-risk non-AC and high-risk AC based chemotherapy; delayed emesis has several approaches with the base of anti-emetics being an NK_1_ receptor antagonist and dexamethasone (Roila et al., 2016; Hesketh et al., 2020). Recent advances have been to include the use of the atypical neuroleptic agent, olanzapine, in situations where nausea and emesis remain problematic. Whilst olanzapine does improve the control of nausea and emesis, it also increases the risks of several unwanted side effects, including sedation (Sutherland et al., 2018). Olanzapine has complex pharmacology and probably involves a combined action at DA, histamine, muscarinic and 5-HT_2_ receptors located in the brain (Rasmussen et al., 2005).

Unfortunately, increased attempts to understand mechanisms involved in nausea have resulted in less attention on mechanisms perturbed in the gastrointestinal tract. However, if all afferent signals derived from the gastrointestinal tract are reduced, then there would be less activation of central mechanisms, and this may represent a more targeted approach to the problems still faced by millions of cancer patients.

The major forces that are required for the vomiting are coordinated mainly through inspiratory and expiratory neurons causing precisely timed contraction of the diaphragm and abdominal and intercostal muscles (Brizzee, 1990). There may also be local changes in gastrointestinal motility patterns involving the enteric nervous system and local mediators (Lang, 1990). The firing of inspiratory and expiratory neurons occurs in the reticular formation of the brainstem as part of the “vomiting center” (Borison and Wang, 1953). The pattern generator for emesis is probably the Botzinger complex which is essentially the pacemaker of respiration; it can also be influenced by afferent input coming from the periphery or other brain areas. Through nerve and brain tissue lesioning experiments, the AP and vagi became the most heavily studied afferent pathways, although input from other “afferents” (e.g., inner ear, vestibular system, hypothalamus, forebrain, etc.) are also well-known (Brizzee, 1990).

The AP (or chemoreceptor trigger zone) is a circumventricular organ (it serves as a link between the brain parenchyma and the cerebrospinal fluid, CSF) that is located on the floor of the fourth ventricle within the dorsal surface of the medulla oblongata. This organ is covered by a thin ependymal cover and is penetrated by fenestrated capillaries (a proper BBB is missing here). Thus, its receptors detect emetic agents in both the blood and the CSF and relay the information to the vomiting center to induce emesis (MacDougall and Sharma, 2021).

The major peripheral nerve at the center of hypotheses to explain chemotherapy-induced emesis is the vagus, the *X*th cranial nerve; splanchnic nerves play a supportive role (Andrews and Rudd, 2004). The vagus also has a vital homeostatic role in gastrointestinal chemosensation following food ingestion (Bai et al., 2019), and, besides the gastrointestinal tract, it also serves other major organ systems including the lungs, heart, and liver (Paintal, 1973). It is therefore involved in a plethora of physiological functions, with cell bodies in the nodose ganglion connecting tissues with the brainstem via termination sites in the nucleus tractus solitarius (NTS), which is below the AP on the floor of the fourth ventricle (Browning, 2015). The vagus is mainly afferent, but efferent neurons originate in the dorsal motor nucleus of the vagus nerve and nucleus ambiguus, which are in close vicinity to the NTS. Neuronal activity in the NTS can therefore be invoked by afferent discharge from multiple organs and afferents coming from the thorax would traverse to the NTS via the cervical vagus along with afferent fibers traveling from the gastrointestinal tract (Babic and Browning, 2014). Electrically stimulating the vagus at the abdominal level can cause retching and vomiting (Makale and King, 1992), but stimulating the cervical vagi has the opposite effect and can prevent the emesis to several challenges (Zabara, 1992). Electrical pacing of the vagus at the cervical level also has the potential to treat epilepsy, depression, headaches, and heart disease, for example (Breit et al., 2018). However, it is also known that stimulation of cardiac vagal afferent fibres can evoke nausea and vomiting (Abrahamsson and Thoren, 1973).

The ability of an animal to vomit has been envisaged to be an evolutionary step for survival against the accidental absorption of potentially dangerous microorganisms, toxins, life-threatening substances, or irritants (Hawthorn et al., 1988). Investigating the role of the vagus nerve and other afferents in mechanisms of emesis control has relied on specialized laboratory animals capable of vomiting, including the cat, dog, monkey, ferret, and shrew (Horn et al., 2013). Abdominal vagotomies are usually carried out with and without a splanchnic nerve section. The impact of these lesions on the latency to either retch and/or vomit, and the number of retches and vomits, has been investigated against a variety of stimuli, usually following 1–2 weeks recovery from surgery. The findings are that lesioning the vagi and/or splanchnic nerves can reduce emesis induced by oral administration of bacterial toxins, gastric irritants, certain drug challenges (receptor-specific, and route of administration-specific) including the early emesis induced by chemotherapeutic agents and radiation (Andrews et al., 1990). Conversely, motion-induced emesis and treatments inducing emesis via the AP are essentially unaffected (Borison and Wang, 1953; Percie du Sert et al., 2010).

Many of the experiments where vagotomy had been employed were against relatively “high doses” of chemotherapeutic drugs in experiments lasting only a few hours. The “high-dose” models have been particularly important in the history of the discovery of the mechanism of action of the 5-HT_3_ receptor antagonists and the concept that 5-HT drives the emetic response after being released from enterochromaffin cells in the gastrointestinal tract (Johnston et al., 2014). It is hypothesized that chemotherapeutic agents may cause free radical damage to cause the 5-HT release from enterochromaffin cells which then activates 5-HT_3_ receptors located on vagal afferents leading to depolarization (Andrews and Rudd, 2004). Evidence for the release of 5-HT by cytotoxic drugs comes from studies on isolated enterochromaffin cells *in vitro* (Schworer et al., 1991; Minami et al., 1997), and also from measurements of ileal dialysates in dogs (Fukui et al., 1993). Increases in the urinary levels of the 5-HT metabolite, 5-HIAA, correlate with periods of nausea and emesis in humans. However, it is also possible that cholecystokinin, glucagon-like peptide-1, and peptide YY, which are released from enteroendocrine cells and are associated with nausea and/or emesis, may also have a local effect on vagal afferents and/or the AP (Andrews and Sanger, 2014).

After a “high” cisplatin dose, released inflammatory mediators (e.g., prostaglandins, bradykinin) may sensitize afferents so that stimulation thresholds are reduced (Andrews and Rudd, 2004). Using a “lower” dose of cisplatin has enabled the development of a model of acute and delayed emesis in ferrets which is pharmacologically similar to man and where emesis can be studied over a 3-day period (Rudd et al., 1994). In this model, bilateral abdominal vagotomy, alone or in combination with greater splanchnic nerve section (7 days prior to challenge), had no effect on the emesis occurring on days 2–3 following cisplatin treatment (Percie du Sert et al., 2009b). Indeed, longer recording times in shrews and pigeons have similarly shown that the protective effect of vagotomy only lasts for a few hours (Tanihata et al., 2000; Sam et al., 2003). Yet in the ferret model, AP ablation protected against delayed emesis and the associated decreases in food and water consumption (Percie du Sert et al., 2009b). If we take these experiments at face value, then we may assume the vagus is important to early emetic mechanisms. However, this assumption must be made cautiously.

As mentioned previously, there is generally a period of recovery between the lesion and the challenge and this delay may have introduced caveats since there is plasticity in the emetic reflex following nerve lesion, whereby the sensitivity of the animal is changed (Andrews et al., 1992). Abdominal vagotomy severs the connection between the gastrointestinal tract and the brain and there would be a loss of mechanisms whereby the peripheral end of the vagus could be activated, either directly or indirectly. There would be time to up- or down-regulation systems to compensate in the brain and gastrointestinal tract. Indeed, there may be local inflammatory changes in the gastrointestinal tract resulting from the degeneration of the severed and perishing peripheral vagal trunk and its nerve endings as they are no longer connected to their ganglia in the neck. Inflammatory mediators and/or other substances released from the gastrointestinal tract could still reach the AP via the circulation, perhaps in higher concentrations (Andrews et al., 1992). To circumvent this problem, investigators surgically prepared ferrets whereby they could cut the vagus at any time during an ongoing emetic response (Andrews et al., 1992). The approach interrupted radiation-induced emesis (Andrews et al., 1992). Unfortunately, no studies have applied this technique at different time points of the acute, nor during the delayed phase of the response to chemotherapy. Therefore, we still await further experimentation in order to be confident of the contribution of the vagus to mechanisms of acute and delayed emesis. If the vagus is indeed found to be important in delayed emesis, it would change our understanding of mechanisms and potentially switch anti-emetic drug discovery efforts away from brainstem mechanisms to the periphery.

The vagus comprises A and B-fibres for visceral and motor input, but it is the C- fibers that carry afferent visceral input that have attracted the most attention (Browning, 2015; Wang et al., 2020); these fibers can be chemically ablated with capsaicin administered neonatally and this has been shown to prevent emesis in adult shrews to resiniferatoxin, a TRPV1 channel opener (Andrews et al., 2000). This has been attempted in a small study in ferrets which suggested a reduction in radiation-induced emesis (Bhandari and Andrews, 1992). It would be of great interest to use neonatal capsaicin to ablate TRPV1 C- fibers to see if chemotherapy-induced emesis could be reduced but the gap between the capsaicin administration and the test would be longer than vagotomy, which would also, therefore, permit plasticity. Resiniferatoxin, an ultra-potent TRPV1 opener administered acutely in ferrets reduced acute and delayed chemotherapy-induced emesis (Yamakuni et al., 2002), whereas the non-pungent TRPV1 opener, olvanil, was ineffective (Chu et al., 2010). Unfortunately, the side effect profiles of TRPV1 ligands have halted further exploration in man (Rudd et al., 2015).

Altered patterns of motility occur as a consequence of several diseases and following drug treatments including chemotherapeutic drugs. Gastric dysrhythmia, which is normally assessed in man by electrogastrography, is commonly associated with nausea and vomiting (Stern et al., 2011). Specifically, gastrointestinal smooth muscle pacing is controlled by rhythmic electrical activity involving calcium currents, or slow waves, generated by interstitial cells of Cajal (ICCs) located within the gut wall (Klein et al., 2013). In humans, the slow wave frequency of the stomach is three counts per minute and the power of the signal can increase as a consequence of stretching the tissue following ingestion of food (Stern et al., 2011). ICCs have an extensive distribution in both circular and longitudinal muscle layers, and myenteric and submucosal plexuses. ICCs express many receptors and ion channels involved in emesis control including those also commonly expressed in enteric neurons (Lee et al., 2017).

In ferrets, high-dose cisplatin decreased the % power of normogastria and increased the % power of bradygastria, without modifying the dominant frequency of the slow waves; these changes were most apparent during the first 2–3 h of 4 h total recording time and were associated with periods of emesis (Percie du Sert et al., 2009a). A study in conscious dogs showed that cisplatin interrupted gastric and intestinal inter-digestive motility with changes in myoelectric activity occurring changes shortly before the onset of emesis (Chey et al., 1988); the changes in myoelectric activity lasted for at least 24 h. Another study in dogs found that cisplatin decreased the time that gastric myoelectric activity (GMA) was in the normal range leading the investigators to conclude that dysrhythmia had occurred during their 6 h recording session (Yu et al., 2009) but the conclusion was based on a simplistic method of analysis, without assessing the power of the slow waves, or if the slow waves were in fact “disordered”. A similar study in dogs showed an increase in gastric dysrhythmia for a period of 6 h following cisplatin (Chen et al., 2011).

Only one study has investigated the effect of chemotherapy on slow waves over a 3-day period in detail using the ferret low-dose cisplatin model. Gastric dysrhythmia was observed during the first 24 h period; there was bradygastria and tachygastria (Lu et al., 2017). During the delayed phase, there was a reduction of the power of the slow waves, and the structure of the slow waves became much more simplistic (Lu et al., 2017). A follow-up study using *Suncus murinus* also revealed changes in GMA (without changes in signal structure) during acute and delayed phases and when tissue was taken at 90 min, the propagation of slow waves was affected in a regional-specific manner (e.g., duodenum and ileum versus stomach and colon), cisplatin also had tissue-specific acute (within minutes) effect on gastrointestinal tissue, *in vitro* (Tu et al., 2021).

Clearly, cisplatin can disrupt slow waves during chemotherapy-induced emesis, which is likely to impact contractile activity and the sensation of nausea. Unfortunately, however, the precise relationship between the slow waves, the vagus, and contractile activity of the gastrointestinal tract is not clearly defined during acute and delayed mechanisms of nausea and emesis. Additional studies are required to fully understand mechanisms of dysrhythmia and the contribution to the side effects of chemotherapy and the interplay between the brain and gastrointestinal tract via the vagus in mechanisms of nausea and emesis.

## Conclusion

Chemotherapeutic drugs may cause a variety of adverse effects, including pain, motor impairment, cognitive dysfunctions, altered emotions, diarrhea, constipation, nausea, and vomiting, all of which are associated at least to some extent, with their neurotoxic potential.

**FIGURE 3:**
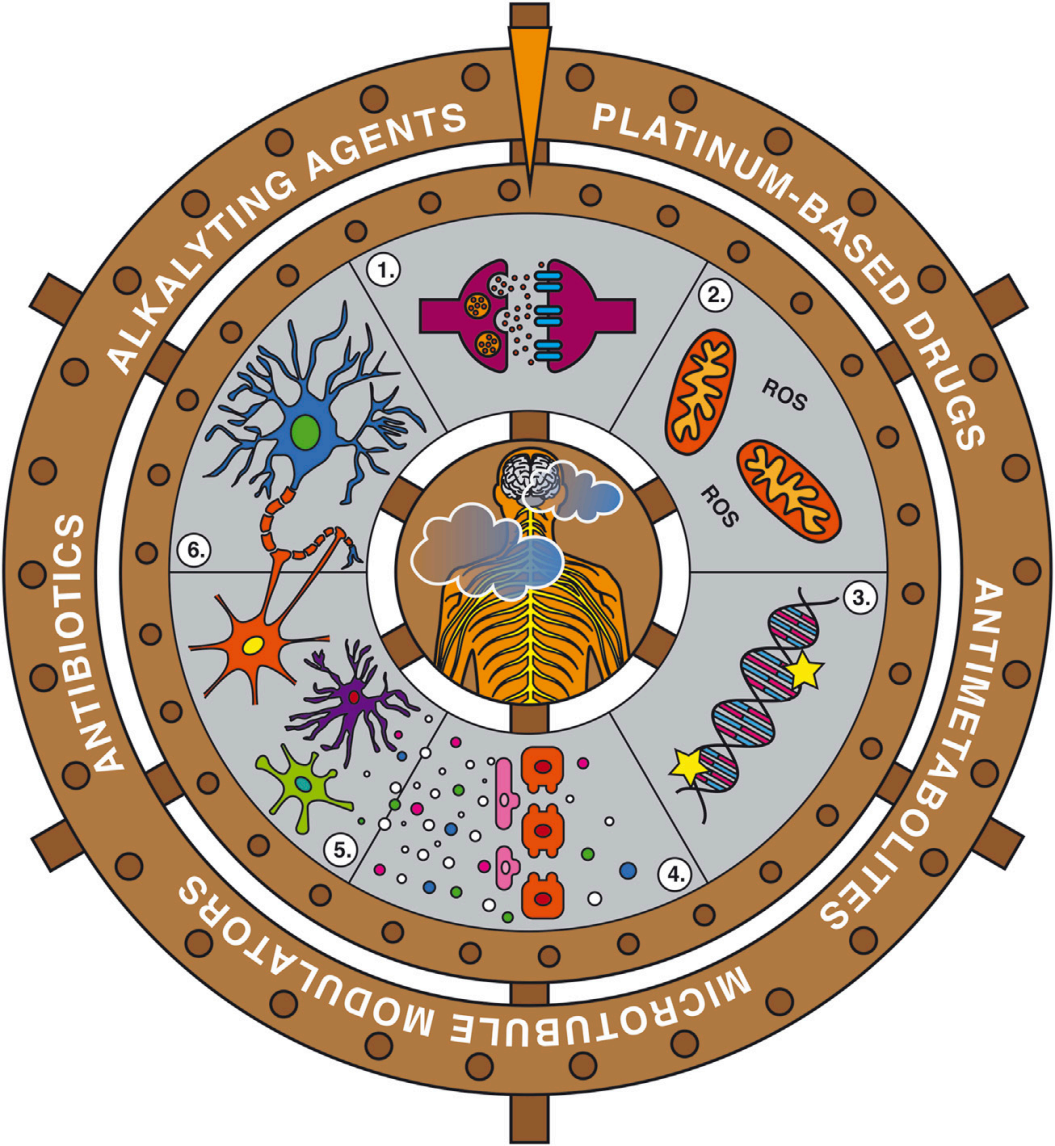
Peripheral and central neurotoxicity - a wheel of bad fortune. In Roman mythology, Fortuna is the goddess, who directs human fate as the deity of both happiness and misfortune. In the art, Fortuna was depicted sitting or standing with a cornucopia and often blindfolded. On the reverse of Roman coins, she is usually shown with a wheel at her side. We propose to consider chemotherapy-induced neurotoxicity (peripheral neuropathy, chemobrain) as a wheel of bad fortune. No matter what chemotherapeutics drugs are used: alkylating agents, platinum-based drugs, antimetabolites, microtubule modulators or antibiotics and what biological mechanisms they trigger: 1. downregulated neurotransmitters’ secretion, 2. overproduction of reactive oxygen species, 3. genetic alterations, 4. neuro-inflammation and breakdown of the blood-brain barrier, 5. reduced gliogenesis and hyperactivation of microglia and astrocytes, 6. impairment in neurogenesis and neural network dynamics, this will lead to the development of nervous dysfunctions (neuropathic pain, cognitive deficits) in the majority of cancer patients.

Neurotoxicity may develop through different modes of action, alone or in combination: direct injury of neurons, impairment of neural network dynamics, reduced neurogenesis and gliogenesis, hyperactivation of supportive glial cells (e.g., astrocytes, microglia, satellite glial cells, enteric glial cells), alterations in the BBB (in chemo-brain), neuro-inflammation and neuroendocrine changes, increased oxidative stress and genetic mutations. Importantly, similar peripheral and central nervous system deficits (that may be long-lasting) are found in many patients, irrespective of the drug(s) used and the mechanisms involved. Therefore, we propose to consider chemotherapy-induced peripheral and central neurotoxicity as a wheel of bad fortune (Figure 3). Importantly, no matter what chemotherapeutic drugs are used and what biological mechanisms they trigger, this might lead to the development of sensory and cognitive deficits in most cancer patients. To turn the wheel of misfortune, we need to recognize the underlying mechanisms of chemotherapy-induced peripheral neuropathy and chemobrain and find ways to overcome them.

Unfortunately, although the typical cancer patient receives a cocktail of drugs during chemotherapy, preclinical findings are of limited translational value because studies aimed at evaluating the neurotoxic effects of combination treatments and those using animal models of various cancers are lacking. Further, most preclinical studies used common laboratory animals incapable of vomiting, and/or short experimentation times, where the more complex situation in humans may not have been truly replicated (Bagues et al., 2022).

In conclusion, there is a need to build upon the vast amount of data in the literature indicating that chemotherapy has caused neurotoxicity and/or caused distress via neuronal mechanisms. Investigating the mechanisms at the molecular level in appropriately designed experiments may provide a way to overcome the effects of neurotoxicity to permit a more aggressive treatment of cancer and improve the quality of life of patients during treatment, and importantly, during recovery from chemotherapy. This will allow us to turn the misfortune wheels associated with peripheral neuropathy, enteric neuropathy, gastric dysrhythmia, and chemobrain, towards a more successful outcome of cancer treatment.

## Glossary

5-FU: 5-Fluorouracil
5-HT: 5-hydroxytryptamine (serotonin)
AC: Anthracyclines and cyclophosphamide
ADORA2A: Adenosine A2A receptor
AMPA-R: α-Amino-3-hydroxy-5-methyl-4-isoxazolepropionic acid receptor
AP: Area postrema
APOE: Apolipoprotein E
ATF3: Activating transcription factor 3
BBB: Blood-Brain Barrier
BER: Berberine
BDNF: Brain-derived neurotrophic factor
CaMKII: Ca2+/calmodulin-dependent protein kinase II
CGRP: calcitonin gene-related peptide
CIPN: Chemotherapy-induced peripheral neuropathy
CNS: Central Nervous System
COMT: Catechol-O-methyltransferase
COX: Cyclooxygenase
CP: Cyclophosphamide
CSF: Cerebrospinal fluid
DA: Dopamine
DNA: Deoxynucleic acid
DCTX: Docetaxel
DOX: Doxorubicin
DRG: Dorsal root ganglia
ENS: Enteric nervous system
ERK2: Extracellular signal–regulated kinase
FOLFOX: 5-Fluorouracil, leukovorin, and oxaliplatin
GABA: γ-Aminobutyric acid
GCEE: γ-Glutamyl cysteine ethyl ester
GMA: Gastric myoelectric activity
GSH: Glutathione
HDAC6: Histone deacetylase 6
HMGB1: High mobility group box-1
ICCs—: Interstitial cells of Cajal
IENF: Intraepidermal nerve fibres
IFN: interferon
IL: Interleukin
iNOS: Inducible nitric oxide synthase
IP: Intraperitoneal injection
IV: Intravenous injections
LPS: Lipopolysaccharide
LTP: Long-term potentiation
MAPK: mitogen-activated protein kinase
MCP-1: Monocyte chemoattractant protein 1
MDR1: Multi-drug resistance -1
MMP: Matrix metallopeptidase
MRP1: Multidrug resistance-associated protein-1
mtDNA: Mitochondrial DNA
MTX: Methotrexate
MWM: Morris water maze
NE: Norepinephrine
NER: Nucleotide excision repair pathway
NF-κB: Nuclear factor κB
NGF: Nerve growth factor
NMDA-R: N-methyl-d-aspartate receptor
nNOS: Neuronal nitric oxide synthase
NOS-: Nitric oxide synthase
NPCs: Neural precursor cells
NSCs: Neuronal stem cells
NTS: Nucleus tractus solitarius
OGG1: 8-Oxoguanine glycosylase
OPCs: Oligodendrocyte precursor cells
PCTX: Paclitaxel
PINK1: PTEN-induced kinase 1
PKA: Protein kinase A
PNS: Peripheral nervous system
PPAR γ: Peroxisome proliferator-activated receptor γ
RAGE: Receptor for advanced glycation end products
RNA: Ribonucleic acid
RNS: Reactive nitrogen species
ROS: Reactive oxygen species
SASP: Senescence-associated secretory phenotype
SGZ: Subgranular zone
SIADH: Syndrome of inappropriate antidiuretic hormone
SIRT1: Sirtuin I
SP: Substance P
SVZ: Subventricular zone
TGF: Transforming growth factor
TLR: Toll-like receptor
TNF-α: Tumor necrosis factor-α
TRP: Transient receptor potential
TS: Thymidylate synthase
VEGF: Vascular endothelial growth factor

## References

[B1] Abalo R. Uranga J. A. Pérez-García I. de Andrés R. Girón R. Vera G. (2017). May Cannabinoids Prevent the Development of Chemotherapy-Induced Diarrhea and Intestinal Mucositis? Experimental Study in the Rat. Neurogastroenterol Motil. 29 (3). 10.1111/nmo.12952 27686064

[B2] Abner C. W. Mckinnon P. J. (2004). The DNA Double-Strand Break Response in the Nervous System. DNA Repair (Amst) 3, 1141–1147. 10.1016/j.dnarep.2004.03.009 15279803

[B3] Abrahamsson H. Thorén P. (1973). Vomiting and Reflex Vagal Relaxation of the Stomach Elicited from Heart Receptors in the Cat. Acta Physiol. Scand. 88, 433–439. 10.1111/j.1748-1716.1973.tb05472.x 4765594

[B4] Acharya M. M. Martirosian V. Chmielewski N. N. Hanna N. Tran K. K. Liao A. C. (2015). Stem Cell Transplantation Reverses Chemotherapy-Induced Cognitive Dysfunction. Cancer Res. 75, 676–686. 10.1158/0008-5472.CAN-14-2237 25687405 PMC4332567

[B5] Adams J. (2002). Development of the Proteasome Inhibitor PS-341. Oncologist 7, 9–16. 10.1634/theoncologist.7-1-9 11854543

[B6] Ahles T. A. Li Y. Mcdonald B. C. Schwartz G. N. Kaufman P. A. Tsongalis G. J. (2014). Longitudinal Assessment of Cognitive Changes Associated with Adjuvant Treatment for Breast Cancer: the Impact of APOE and Smoking. Psychooncology 23, 1382–1390. 10.1002/pon.3545 24789331 PMC4214914

[B7] Ahles T. A. Saykin A. J. (2007). Candidate Mechanisms for Chemotherapy-Induced Cognitive Changes. Nat. Rev. Cancer 7, 192–201. 10.1038/nrc2073 17318212 PMC3329763

[B8] Ahles T. A. Saykin A. J. Noll W. W. Furstenberg C. T. Guerin S. Cole B. (2003). The Relationship of APOE Genotype to Neuropsychological Performance in Long-Term Cancer Survivors Treated with Standard Dose Chemotherapy. Psychooncology 12, 612–619. 10.1002/pon.742 12923801

[B9] Alé A. Bruna J. Morell M. van de Velde H. Monbaliu J. Navarro X. (2014). Treatment with Anti-TNF Alpha Protects against the Neuropathy Induced by the Proteasome Inhibitor Bortezomib in a Mouse Model. Exp. Neurol. 253, 165–173. 10.1016/j.expneurol.2013.12.020 24406455

[B10] Alhowail A. H. Bloemer J. Majrashi M. Pinky P. D. Bhattacharya S. Yongli Z. (2019). Doxorubicin-induced Neurotoxicity Is Associated with Acute Alterations in Synaptic Plasticity, Apoptosis, and Lipid Peroxidation. Toxicol. Mech. Methods 29, 457–466. 10.1080/15376516.2019.1600086 31010378

[B11] Aluise C. D. Miriyala S. Noel T. Sultana R. Jungsuwadee P. Taylor T. J. (2011). 2-Mercaptoethane Sulfonate Prevents Doxorubicin-Induced Plasma Protein Oxidation and TNF-α Release: Implications for the Reactive Oxygen Species-Mediated Mechanisms of Chemobrain. Free Radic. Biol. Med. 50, 1630–1638. 10.1016/j.freeradbiomed.2011.03.009 21421044

[B12] André T. Bensmaine M. A. Louvet C. François E. Lucas V. Desseigne F. (1999). Multicenter Phase II Study of Bimonthly High-Dose Leucovorin, Fluorouracil Infusion, and Oxaliplatin for Metastatic Colorectal Cancer Resistant to the Same Leucovorin and Fluorouracil Regimen. J. Clin. Oncol. 17, 3560–3568. 10550155 10.1200/JCO.1999.17.11.3560

[B13] Andres A. L. Gong X. Di K. Bota D. A. (2014). Low-doses of Cisplatin Injure Hippocampal Synapses: a Mechanism for 'chemo' Brain? Exp. Neurol. 255, 137–144. 10.1016/j.expneurol.2014.02.020 24594220 PMC4059602

[B14] Andrews P. L. Davis C. J. Bingham S. Davidson H. I. Hawthorn J. Maskell L. (1990). The Abdominal Visceral Innervation and the Emetic Reflex: Pathways, Pharmacology, and Plasticity. Can. J. Physiol. Pharmacol. 68, 325–345. 10.1139/y90-047 2178756

[B15] Andrews P. L. Okada F. Woods A. J. Hagiwara H. Kakaimoto S. Toyoda M. (2000). The Emetic and Anti-emetic Effects of the Capsaicin Analogue Resiniferatoxin in Suncus Murinus, the House Musk Shrew. Br. J. Pharmacol. 130, 1247–1254. 10.1038/sj.bjp.0703428 10903962 PMC1572188

[B16] Andrews P. L. Sanger G. J. (2014). Nausea and the Quest for the Perfect Anti-emetic. Eur. J. Pharmacol. 722, 108–121. 10.1016/j.ejphar.2013.09.072 24157981

[B17] Andrews P. L. R. Bhandari P. Davis C. J. (1992). “Pasticity and Modulation of the Emetic Reflex,” in Mechanisms and Control of Emesis. Editors Bianchi A L. Grelot L. Miller A. D. King G. L. (Montrouge, France: John Libby Eurotext Ltd).

[B18] Andrews P. L. R. Rudd J. A. (2004). “Mechanisms of Acute, Delayed and Anticipatory Emesis Induced by Anti-cancer Therapies,” in Management of Nausea and Vomiting in Cancer and Cancer Treatment. Editor Hesketh P. (London, U.K.: Barlett Publisers Inc), 15–66.

[B19] Arbuckle R. B. Huber S. L. Zacker C. (2000). The Consequences of Diarrhea Occurring during Chemotherapy for Colorectal Cancer: a Retrospective Study. Oncologist 5, 250–259. 10.1634/theoncologist.5-3-250 10884503

[B20] Argyriou A. A. Assimakopoulos K. Iconomou G. Giannakopoulou F. Kalofonos H. P. (2011). Either called "chemobrain" or "chemofog," the Long-Term Chemotherapy-Induced Cognitive Decline in Cancer Survivors Is Real. J. Pain Symptom Manage. 41, 126–139. 10.1016/j.jpainsymman.2010.04.021 20832978

[B21] Atarod D. Eskandari-Sedighi G. Pazhoohi F. Karimian S. M. Khajeloo M. Riazi G. H. (2015). Microtubule Dynamicity Is More Important Than Stability in Memory Formation: an *In Vivo* Study. J. Mol. Neurosci. 56, 313–319. 10.1007/s12031-015-0535-4 25740015

[B22] Aviello G. Knaus U. G. (2017). ROS in Gastrointestinal Inflammation: rescue or Sabotage? Br. J. Pharmacol. 174, 1704–1718. 10.1111/bph.13428 26758851 PMC5446568

[B23] Babic T. Browning K. N. (2014). The Role of Vagal Neurocircuits in the Regulation of Nausea and Vomiting. Eur. J. Pharmacol. 722, 38–47. 10.1016/j.ejphar.2013.08.047 24184670 PMC3893663

[B24] Bagues A. López-Tofiño Y. Llorente-Berzal A. Abalo R. (2022). Cannabinoid Drugs against Chemotherapy-Induced Adverse Effects: Focus on Nausea/vomiting, Peripheral Neuropathy and Chemofog in Animal Models. Behav. Pharmacol. 17. Online ahead of print. 10.1097/FBP.0000000000000667 35045012

[B25] Bai L. Mesgarzadeh S. Ramesh K. S. Huey E. L. Liu Y. Gray L. A. (2019). Genetic Identification of Vagal Sensory Neurons that Control Feeding. Cell 179, 1129–e23. 10.1016/j.cell.2019.10.031 31730854 PMC6916730

[B26] Banach M. Juranek J. K. Zygulska A. L. (2017). Chemotherapy-induced Neuropathies-A Growing Problem for Patients and Health Care Providers. Brain Behav. 7 (1), e00558. 10.1002/brb3.558 28127506 PMC5256170

[B27] Banks W. A. Erickson M. A. (2010). The Blood-Brain Barrier and Immune Function and Dysfunction. Neurobiol. Dis. 37, 26–32. 10.1016/j.nbd.2009.07.031 19664708

[B28] Barnes A. P. Polleux F. (2009). Establishment of Axon-Dendrite Polarity in Developing Neurons. Annu. Rev. Neurosci. 32, 347–381. 10.1146/annurev.neuro.31.060407.125536 19400726 PMC3170863

[B29] Bechakra M. Nieuwenhoff M. D. van Rosmalen J. Groeneveld G. J. Scheltens-de Boer M. Sonneveld P. (2018). Clinical, Electrophysiological, and Cutaneous Innervation Changes in Patients with Bortezomib-Induced Peripheral Neuropathy Reveal Insight into Mechanisms of Neuropathic Pain. Mol. Pain 14, 1744806918797042. 10.1177/1744806918797042 30152246 PMC6113731

[B30] Belsky J. A. Wolf K. Setty B. A. (2020). A Case of Resolved Vincristine-Induced Constipation Following Osteopathic Medicine in a Patient with Infantile Fibrosarcoma. J. Am. Osteopath Assoc. 120, 691–695. 10.7556/jaoa.2020.102 32926170

[B31] Bendlin B. B. Fitzgerald M. E. Ries M. L. Xu G. Kastman E. K. Thiel B. W. (2010). White Matter in Aging and Cognition: a Cross-Sectional Study of Microstructure in Adults Aged Eighteen to Eighty-Three. Dev. Neuropsychol. 35, 257–277. 10.1080/87565641003696775 20446132 PMC2895988

[B32] Bhandari P. Andrews P. L. R. (1992). “Resiniferatoxin: a Broad Spectrum Antiemetic in the Ferret,” in Mechanisms and Control of Emesis. Editors Bianchi A. L. Grelot L. Miller A. D. King G. L. (Montrouge, France: John Libby Eurotext Ltd.), 239–240.

[B33] Bobylev I. Joshi A. R. Barham M. Ritter C. Neiss W. F. Höke A. (2015). Paclitaxel Inhibits mRNA Transport in Axons. Neurobiol. Dis. 82, 321–331. 10.1016/j.nbd.2015.07.006 26188177

[B34] Boehmerle W. Huehnchen P. Peruzzaro S. Balkaya M. Endres M. (2014). Electrophysiological, Behavioral and Histological Characterization of Paclitaxel, Cisplatin, Vincristine and Bortezomib-Induced Neuropathy in C57Bl/6 Mice. Sci. Rep. 4, 6370. 10.1038/srep06370 25231679 PMC5377307

[B35] Bompaire F. Durand T. Léger-Hardy I. Psimaras D. Ricard D. (2017). Chemotherapy-related Cognitive Impairment or « chemobrain »: Concept and State of Art. Geriatr. Psychol. Neuropsychiatr. Vieil 15, 89–98. 10.1684/pnv.2017.0659 28266346

[B36] Bon-Frauches A. C. Boesmans W. (2020). The Enteric Nervous System: the Hub in a star Network. Nat. Rev. Gastroenterol. Hepatol. 17, 717–718. 10.1038/s41575-020-00377-2 33087897

[B37] Borison H. L. Wang S. C. (1953). Physiology and Pharmacology of Vomiting. Pharmacol. Rev. 5, 193–230. 13064033

[B38] Bornstein J. Gwynne R. Sjövall H. (2012). “Enteric Neural Regulation of Mucosal Secretion,” in Physiology of the Gastrointestinal Tract. 5th Edition (Amsterdam, Netherlands: Elsevier). 10.1016/b978-0-12-382026-6.00027-0

[B39] Boullon L. Abalo R. Llorente-Berzal Á. (2021). Cannabinoid Drugs-Related Neuroprotection as a Potential Therapeutic Tool against Chemotherapy-Induced Cognitive Impairment. Front. Pharmacol. 12, 734613. 10.3389/fphar.2021.734613 34867342 PMC8632779

[B40] Bower J. E. Ganz P. A. Aziz N. Olmstead R. Irwin M. R. Cole S. W. (2007). Inflammatory Responses to Psychological Stress in Fatigued Breast Cancer Survivors: Relationship to Glucocorticoids. Brain Behav. Immun. 21, 251–258. 10.1016/j.bbi.2006.08.001 17008048

[B41] Branca J. J. V. Maresca M. Morucci G. Becatti M. Paternostro F. Gulisano M. (2018). Oxaliplatin-induced Blood Brain Barrier Loosening: a New point of View on Chemotherapy-Induced Neurotoxicity. Oncotarget 9, 23426–23438. 10.18632/oncotarget.25193 29805744 PMC5955120

[B42] Brandolini L. D'angelo M. Antonosante A. Allegretti M. Cimini A. (2019). Chemokine Signaling in Chemotherapy-Induced Neuropathic Pain. Int. J. Mol. Sci. 20. 10.3390/ijms20122904 PMC6627296 31197114

[B43] Brehmer A. (2021). Classification of Human Enteric Neurons. Histochem. Cell Biol 156, 95–108. 10.1007/s00418-021-02002-y 34170401 PMC8397665

[B44] Breit S. Kupferberg A. Rogler G. Hasler G. (2018). Vagus Nerve as Modulator of the Brain-Gut Axis in Psychiatric and Inflammatory Disorders. Front. Psychiatry 9, 44–15. 10.3389/fpsyt.2018.00044 29593576 PMC5859128

[B45] Briani C. Zara G. Rondinone R. Della Libera S. Ermani M. Ruggero S. (2004). Thalidomide Neurotoxicity: Prospective Study in Patients with Lupus Erythematosus. Neurology 62, 2288–2290. 10.1212/01.wnl.0000130499.91775.2c 15210897

[B46] Briones T. L. Woods J. (2011). Chemotherapy-induced Cognitive Impairment Is Associated with Decreases in Cell Proliferation and Histone Modifications. BMC Neurosci. 12, 124. 10.1186/1471-2202-12-124 22152030 PMC3252305

[B47] Brizzee K. R. (1990). Mechanics of Vomiting: a Minireview. Can. J. Physiol. Pharmacol. 68, 221–229. 10.1139/y90-035 2178746

[B48] Brouwers E. E. Huitema A. D. Beijnen J. H. Schellens J. H. (2008). Long-term Platinum Retention after Treatment with Cisplatin and Oxaliplatin. BMC Clin. Pharmacol. 8, 7. 10.1186/1472-6904-8-7 18796166 PMC2559818

[B49] Browning K. N. (2015). Role of central Vagal 5-HT3 Receptors in Gastrointestinal Physiology and Pathophysiology. Front. Neurosci. 9, 413. 10.3389/fnins.2015.00413 26578870 PMC4625078

[B50] Buccafusca G. Proserpio I. Tralongo A. C. Rametta Giuliano S. Tralongo P. (2019). Early Colorectal Cancer: Diagnosis, Treatment and Survivorship Care. Crit. Rev. Oncol. Hematol. 136, 20–30. 10.1016/j.critrevonc.2019.01.023 30878125

[B51] Cabezos P. A. Vera G. Castillo M. Fernández-Pujol R. Martín M. I. Abalo R. (2008). Radiological Study of Gastrointestinal Motor Activity after Acute Cisplatin in the Rat. Temporal Relationship with pica. Auton. Neurosci. 141 (1-2), 54–65. 10.1016/j.autneu.2008.05.004 18579450

[B52] Cabezos P. A. Vera G. Martín-Fontelles M. I. Fernández-Pujol R. Abalo R. (2010). Cisplatin-induced Gastrointestinal Dysmotility Is Aggravated after Chronic Administration in the Rat. Comparison with pica. Neurogastroenterol Motil. 22 (7), 797–805. 10.1111/j.1365-2982.2010.01483.x 20236245

[B53] Campisi J. D'adda Di Fagagna F. (2007). Cellular Senescence: when Bad Things Happen to Good Cells. Nat. Rev. Mol. Cell Biol 8, 729–740. 10.1038/nrm2233 17667954

[B54] Canta A. Pozzi E. Carozzi V. A. (2015). Mitochondrial Dysfunction in Chemotherapy-Induced Peripheral Neuropathy (CIPN). Toxics 3, 198–223. 10.3390/toxics3020198 29056658 PMC5634687

[B55] Carbone S. E. Jovanovska V. Brookes S. J. Nurgali K. (2016). Electrophysiological and Morphological Changes in Colonic Myenteric Neurons from Chemotherapy-Treated Patients: a Pilot Study. Neurogastroenterol Motil. 28, 975–984. 10.1111/nmo.12795 26909894 PMC5215581

[B56] Carozzi V. A. Canta A. Oggioni N. Sala B. Chiorazzi A. Meregalli C. (2010). Neurophysiological and Neuropathological Characterization of New Murine Models of Chemotherapy-Induced Chronic Peripheral Neuropathies. Exp. Neurol. 226, 301–309. 10.1016/j.expneurol.2010.09.004 20832406

[B57] Carozzi V. A. Renn C. L. Bardini M. Fazio G. Chiorazzi A. Meregalli C. (2013). Bortezomib-Induced Painful Peripheral Neuropathy: An Electrophysiological, Behavioral, Morphological and Mechanistic Study in the Mouse. PLoS One 8 (9), e72995. 10.1371/journal.pone.0072995 24069168 PMC3772181

[B58] Castelli V. Palumbo P. d'Angelo M. Moorthy N. K. Antonosante A. Catanesi M. (2018). Probiotic DSF Counteracts Chemotherapy Induced Neuropathic Pain. Oncotarget 9, 27998–28008. 10.18632/oncotarget.25524 29963257 PMC6021327

[B59] Cavaletti G. Gilardini A. Canta A. Rigamonti L. Rodriguez-Menendez V. Ceresa C. (2007). Bortezomib-induced Peripheral Neurotoxicity: A Neurophysiological and Pathological Study in the Rat. Exp. Neurol. 204, 317–325. 10.1016/j.expneurol.2006.11.010 17214983

[B60] Chen B. T. Ye N. Wong C. W. Patel S. K. Jin T. Sun C. L. (2020). Effects of Chemotherapy on Aging white Matter Microstructure: A Longitudinal Diffusion Tensor Imaging Study. J. Geriatr. Oncol. 11, 290–296. 10.1016/j.jgo.2019.09.016 31685415 PMC7054164

[B61] Chen J. H. Song G. Q. Yin J. Sun Y. Chen J. D. (2011). Gastric Electrical Stimulation Reduces Visceral Sensitivity to Gastric Distention in Healthy Canines. Auton. Neurosci. 160, 16–20. 10.1016/j.autneu.2010.10.009 21126929 PMC3034808

[B62] Chen Y. Jungsuwadee P. Vore M. Butterfield D. A. St Clair D. K. (2007). Collateral Damage in Cancer Chemotherapy: Oxidative Stress in Nontargeted Tissues. Mol. Interv. 7, 147–156. 10.1124/mi.7.3.6 17609521

[B63] Cheng W. Bishop A. E. Spitz L. Polak J. M. (1999). Abnormal Enteric Nerve Morphology in Atretic Esophagus of Fetal Rats with Adriamycin-Induced Esophageal Atresia. Pediatr. Surg. Int. 15, 8–10. 10.1007/s003830050500 9914345

[B64] Cheng W. Bishop A. E. Spitz L. Polak J. M. (1997). Abnormalities of Neuropeptides and Neural Markers in the Esophagus of Fetal Rats with Adriamycin-Induced Esophageal Atresia. J. Pediatr. Surg. 32, 1420–1423. 10.1016/s0022-3468(97)90552-2 9349759

[B65] Chey R. D. Lee K. Y. Asbury R. Chey W. Y. (1988). Effect of Cisplatin on Myoelectric Activity of the Stomach and Small Intestine in Dogs. Dig. Dis. Sci. 33, 338–344. 10.1007/BF01535760 3342726

[B66] Chiorazzi A. Semperboni S. Marmiroli P. (2015). Current View in Platinum Drug Mechanisms of Peripheral Neurotoxicity. Toxics 3, 304–321. 10.3390/toxics3030304 29051466 PMC5606682

[B67] Choi R. Goldstein B. J. (2018). Olfactory Epithelium: Cells, Clinical Disorders, and Insights from an Adult Stem Cell Niche. Laryngoscope Investig. Otolaryngol. 3, 35–42. 10.1002/lio2.135 PMC5824112 29492466

[B68] Christie L. A. Acharya M. M. Parihar V. K. Nguyen A. Martirosian V. Limoli C. L. (2012). Impaired Cognitive Function and Hippocampal Neurogenesis Following Cancer Chemotherapy. Clin. Cancer Res. 18, 1954–1965. 10.1158/1078-0432.CCR-11-2000 22338017

[B69] Chu K. M. Ngan M. P. Wai M. K. Yeung C. K. Andrews P. L. Percie du Sert N. (2010). Olvanil, a Non-pungent Vanilloid Enhances the Gastrointestinal Toxicity of Cisplatin in the Ferret. Toxicol. Lett. 192, 402–407. 10.1016/j.toxlet.2009.11.015 19931602

[B70] Cole P. D. Finkelstein Y. Stevenson K. E. Blonquist T. M. Vijayanathan V. Silverman L. B. (2015). Polymorphisms in Genes Related to Oxidative Stress Are Associated with Inferior Cognitive Function after Therapy for Childhood Acute Lymphoblastic Leukemia. J. Clin. Oncol. 33, 2205–2211. 10.1200/JCO.2014.59.0273 25987702 PMC4477790

[B71] Collado-Hidalgo A. Bower J. E. Ganz P. A. Cole S. W. Irwin M. R. (2006). Inflammatory Biomarkers for Persistent Fatigue in Breast Cancer Survivors. Clin. Cancer Res. 12, 2759–2766. 10.1158/1078-0432.CCR-05-2398 16675568

[B72] Cook B. M. Wozniak K. M. Proctor D. A. Bromberg R. B. Wu Y. Slusher B. S. (2018). Differential Morphological and Biochemical Recovery from Chemotherapy-Induced Peripheral Neuropathy Following Paclitaxel, Ixabepilone, or Eribulin Treatment in Mouse Sciatic Nerves. Neurotox. Res. 34, 677–692. 10.1007/s12640-018-9929-8 30051419

[B73] Cronstein B. N. Naime D. Ostad E. (1993). The Antiinflammatory Mechanism of Methotrexate. Increased Adenosine Release at Inflamed Sites Diminishes Leukocyte Accumulation in an *In Vivo* Model of Inflammation. J. Clin. Invest. 92, 2675–2682. 10.1172/JCI116884 8254024 PMC288465

[B74] Cuozzo M. Castelli V. Avagliano C. Cimini A. d'Angelo M. Cristiano C. (2021). Effects of Chronic Oral Probiotic Treatment in Paclitaxel-Induced Neuropathic Pain. Biomedicines 9 (4), 346. 10.3390/biomedicines9040346 33808052 PMC8066538

[B75] Dahlgren D. Sjöblom M. Hellström P. M. Lennernäs H. (2021). Chemotherapeutics-Induced Intestinal Mucositis: Pathophysiology and Potential Treatment Strategies. Front. Pharmacol. 12, 681417. 10.3389/fphar.2021.681417 34017262 PMC8129190

[B76] De Biasi A. R. Villena-Vargas J. Adusumilli P. S. (2014). Cisplatin-induced Antitumor Immunomodulation: A Review of Preclinical and Clinical Evidence. Clin. Cancer Res. 20, 5384–5391. 10.1158/1078-0432.CCR-14-1298 25204552 PMC4216745

[B77] de Gramont A. Figer A. Seymour M. Homerin M. Hmissi A. Cassidy J. (2000). Leucovorin and Fluorouracil with or without Oxaliplatin as First-Line Treatment in Advanced Colorectal Cancer. J. Clin. Oncol. 18, 2938–2947. 10.1200/JCO.2000.18.16.2938 10944126

[B78] De Iuliis F. Taglieri L. Salerno G. Lanza R. Scarpa S. (2015). Taxane Induced Neuropathy in Patients Affected by Breast Cancer: Literature Review. Crit. Rev. Oncol. Hematol. 96, 34–45. 10.1016/j.critrevonc.2015.04.011 26004917

[B79] Demby T. C. Rodriguez O. Mccarthy C. W. Lee Y. C. Albanese C. Mandelblatt J. (2020). A Mouse Model of Chemotherapy-Related Cognitive Impairments Integrating the Risk Factors of Aging and APOE4 Genotype. Behav. Brain Res. 384, 112534. 10.1016/j.bbr.2020.112534 32027870 PMC7082850

[B80] Denlinger C. S. Barsevick A. M. (2009). The Challenges of Colorectal Cancer Survivorship. J. Natl. Compr. Canc Netw. 7, 883–894. 10.6004/jnccn.2009.0058 19755048 PMC3110673

[B81] Deprez S. Amant F. Smeets A. Peeters R. Leemans A. Van Hecke W. (2012). Longitudinal Assessment of Chemotherapy-Induced Structural Changes in Cerebral white Matter and its Correlation with Impaired Cognitive Functioning. J. Clin. Oncol. 30, 274–281. 10.1200/JCO.2011.36.8571 22184379

[B82] Deprez S. Amant F. Yigit R. Porke K. Verhoeven J. Van Den Stock J. (2011). Chemotherapy-induced Structural Changes in Cerebral white Matter and its Correlation with Impaired Cognitive Functioning in Breast Cancer Patients. Hum. Brain Mapp. 32, 480–493. 10.1002/hbm.21033 20725909 PMC6870393

[B83] Dheen S. T. Kaur C. Ling E. A. (2007). Microglial Activation and its Implications in the Brain Diseases. Curr. Med. Chem. 14, 1189–1197. 10.2174/092986707780597961 17504139

[B84] Dhillon H. M. Tannock I. F. Pond G. R. Renton C. Rourke S. B. Vardy J. L. (2018). Perceived Cognitive Impairment in People with Colorectal Cancer Who Do and Do Not Receive Chemotherapy. J. Cancer Surviv 12, 178–185. 10.1007/s11764-017-0656-6 29080061

[B85] Dietrich J. Han R. Yang Y. Mayer-Pröschel M. Noble M. (2006). CNS Progenitor Cells and Oligodendrocytes Are Targets of Chemotherapeutic Agents *In Vitro* and *In Vivo* . J. Biol. 5, 22. 10.1186/jbiol50 17125495 PMC2000477

[B86] Dietrich J. Prust M. Kaiser J. (2015). Chemotherapy, Cognitive Impairment and Hippocampal Toxicity. Neuroscience 309, 224–232. 10.1016/j.neuroscience.2015.06.016 26086545

[B87] Dilruba S. Kalayda G. V. (2016). Platinum-based Drugs: Past, Present and Future. Cancer Chemother. Pharmacol. 77, 1103–1124. 10.1007/s00280-016-2976-z 26886018

[B88] Donald E. L. Stojanovska L. Apostolopoulos V. Nurgali K. (2017). Resveratrol Alleviates Oxidative Damage in Enteric Neurons and Associated Gastrointestinal Dysfunction Caused by Chemotherapeutic Agent Oxaliplatin. Maturitas 105, 100–106. 10.1016/j.maturitas.2017.05.010 28545905

[B89] Dooley L. N. Ganz P. A. Cole S. W. Crespi C. M. Bower J. E. (2016). Val66Met BDNF Polymorphism as a Vulnerability Factor for Inflammation-Associated Depressive Symptoms in Women with Breast Cancer. J. Affect Disord. 197, 43–50. 10.1016/j.jad.2016.02.059 26967918 PMC4836957

[B90] Duan Z. Su Z. Wang H. Pang X. (2018). Involvement of Pro-inflammation Signal Pathway in Inhibitory Effects of Rapamycin on Oxaliplatin-Induced Neuropathic Pain. Mol. Pain 14, 1744806918769426. 10.1177/1744806918769426 29587559 PMC5898663

[B91] Dubey J. Ratnakaran N. Koushika S. P. (2015). Neurodegeneration and Microtubule Dynamics: Death by a Thousand Cuts. Front Cell Neurosci 9, 343. 10.3389/fncel.2015.00343 26441521 PMC4563776

[B92] Duggett N. A. Flatters S. J. L. (2017). Characterization of a Rat Model of Bortezomib-Induced Painful Neuropathy. Br. J. Pharmacol. 174, 4812–4825. 10.1111/bph.14063 28972650 PMC5727311

[B93] Dzagnidze A. Katsarava Z. Makhalova J. Liedert B. Yoon M. S. Kaube H. (2007). Repair Capacity for Platinum-DNA Adducts Determines the Severity of Cisplatin-Induced Peripheral Neuropathy. J. Neurosci. 27, 9451–9457. 10.1523/JNEUROSCI.0523-07.2007 17728458 PMC6673116

[B94] El-Agamy S. E. Abdel-Aziz A. K. Wahdan S. Esmat A. Azab S. S. (2018). Astaxanthin Ameliorates Doxorubicin-Induced Cognitive Impairment (Chemobrain) in Experimental Rat Model: Impact on Oxidative, Inflammatory, and Apoptotic Machineries. Mol. Neurobiol. 55, 5727–5740. 10.1007/s12035-017-0797-7 29039023

[B95] Elbeltagy M. Mustafa S. Umka J. Lyons L. Salman A. Chur-Yoe G. T. (2010). Fluoxetine Improves the Memory Deficits Caused by the Chemotherapy Agent 5-fluorouracil. Behav. Brain Res. 208, 112–117. 10.1016/j.bbr.2009.11.017 19914299

[B96] Erenpreisa J. Salmina K. Huna A. Jackson T. R. Vazquez-Martin A. Cragg M. S. (2015). The "virgin Birth", Polyploidy, and the Origin of Cancer. Oncoscience 2, 3–14. 10.18632/oncoscience.108 25821840 PMC4341460

[B97] Ernst A. Alkass K. Bernard S. Salehpour M. Perl S. Tisdale J. (2014). Neurogenesis in the Striatum of the Adult Human Brain. Cell 156, 1072–1083. 10.1016/j.cell.2014.01.044 24561062

[B98] Escalante J. McQuade R. M. Stojanovska V. Nurgali K. (2017). Impact of Chemotherapy on Gastrointestinal Functions and the Enteric Nervous System. Maturitas 105, 23–29. 10.1016/j.maturitas.2017.04.021 28545907

[B99] Ewert de Oliveira B. Junqueira Amorim O. H. Lima L. L. Rezende R. A. Mestnik N. C. Bagatin E. (2021). 5-Fluorouracil, Innovative Drug Delivery Systems to Enhance Bioavailability for Topical Use. J. Drug Deliv. Sci. Technology 61, 102155. 10.1016/j.jddst.2020.102155

[B100] Fahim A. Rehman Z. Bhatti M. F. Virk N. Ali A. Rashid A. (2019). The Route to 'Chemobrain' - Computational Probing of Neuronal LTP Pathway. Sci. Rep. 9, 9630. 10.1038/s41598-019-45883-9 31270411 PMC6610097

[B101] Falzone L. Salomone S. Libra M. (2018). Evolution of Cancer Pharmacological Treatments at the Turn of the Third Millennium. Front. Pharmacol. 9, 1300. 10.3389/fphar.2018.01300 30483135 PMC6243123

[B102] Fardell J. E. Vardy J. Shah J. D. Johnston I. N. (2012). Cognitive Impairments Caused by Oxaliplatin and 5-fluorouracil Chemotherapy Are Ameliorated by Physical Activity. Psychopharmacology (Berl) 220, 183–193. 10.1007/s00213-011-2466-2 21894483

[B103] Fardell J. E. Zhang J. De Souza R. Vardy J. Johnston I. Allen C. (2014). The Impact of Sustained and Intermittent Docetaxel Chemotherapy Regimens on Cognition and Neural Morphology in Healthy Mice. Psychopharmacology (Berl) 231, 841–852. 10.1007/s00213-013-3301-8 24101158

[B104] Fernandez H. R. Varma A. Flowers S. A. Rebeck G. W. (2020). Cancer Chemotherapy Related Cognitive Impairment and the Impact of the Alzheimer's Disease Risk Factor APOE. Cancers (Basel) 12, 3842. 10.3390/cancers12123842 PMC7766535 33352780

[B105] Fernyhough P. Smith D. R. Schapansky J. Van Der Ploeg R. Gardiner N. J. Tweed C. W. (2005). Activation of Nuclear Factor-kappaB via Endogenous Tumor Necrosis Factor Alpha Regulates Survival of Axotomized Adult Sensory Neurons. J. Neurosci. 25, 1682–1690. 10.1523/JNEUROSCI.3127-04.2005 15716404 PMC6725919

[B106] Field T. M. Shin M. Stucky C. S. Loomis J. Johnson M. A. (2018). Electrochemical Measurement of Dopamine Release and Uptake in Zebrafish Following Treatment with Carboplatin. Chemphyschem 19, 1192–1196. 10.1002/cphc.201701357 29573086 PMC6013284

[B107] Fields R. D. Araque A. Johansen-Berg H. Lim S. S. Lynch G. Nave K. A. (2014). Glial Biology in Learning and Cognition. Neuroscientist 20, 426–431. 10.1177/1073858413504465 24122821 PMC4161624

[B108] Fischer J. Robin Ganellin C. (2006). Analogue-based Drug Discovery. Hoboken, NJ: John Wiley & Sons, 1–575.

[B109] Fischer M. Schmutzhard E. (2017). Posterior Reversible Encephalopathy Syndrome. J. Neurol. 264, 1608–1616. 10.1007/s00415-016-8377-8 28054130 PMC5533845

[B110] Flatters S. J. Bennett G. J. (2006). Studies of Peripheral Sensory Nerves in Paclitaxel-Induced Painful Peripheral Neuropathy: Evidence for Mitochondrial Dysfunction. Pain 122, 245–257. 10.1016/j.pain.2006.01.037 16530964 PMC1805481

[B111] Fleming M. A. Ehsan L. Moore S. R. Levin D. E. (2020). The Enteric Nervous System and its Emerging Role as a Therapeutic Target. Gastroenterol. Res. Pract. 2020, 8024171. 10.1155/2020/8024171 32963521 PMC7495222

[B112] Fliessbach K. Helmstaedter C. Urbach H. Althaus A. Pels H. Linnebank M. (2005). Neuropsychological Outcome after Chemotherapy for Primary CNS Lymphoma: a Prospective Study. Neurology 64, 1184–1188. 10.1212/01.WNL.0000156350.49336.E2 15824344

[B113] Forrest M. P. Parnell E. Penzes P. (2018). Dendritic Structural Plasticity and Neuropsychiatric Disease. Nat. Rev. Neurosci. 19, 215–234. 10.1038/nrn.2018.16 29545546 PMC6442683

[B114] Fournier E. Passirani C. Montero-Menei C. Colin N. Breton P. Sagodira S. (2003). Therapeutic Effectiveness of Novel 5-Fluorouracil-Loaded Poly(methylidene Malonate 2.1.2)-based Microspheres on F98 Glioma-Bearing Rats. Cancer 97, 2822–2829. 10.1002/cncr.11388 12767096

[B115] Fukui H. Yamamoto M. Ando T. Sasaki S. Sato S. (1993). Increase in Serotonin Levels in the Dog Ileum and Blood by Cisplatin as Measured by Microdialysis. Neuropharmacology 32, 959–968. 10.1016/0028-3908(93)90060-g 8295718

[B116] Fumagalli G. Monza L. Cavaletti G. Rigolio R. Meregalli C. (2020). Neuroinflammatory Process Involved in Different Preclinical Models of Chemotherapy-Induced Peripheral Neuropathy. Front. Immunol. 11, 626687. 10.3389/fimmu.2020.626687 33613570 PMC7890072

[B117] Furness J. B. (2012). The Enteric Nervous System and Neurogastroenterology. Nat. Rev. Gastroenterol. Hepatol. 9, 286–294. 10.1038/nrgastro.2012.32 22392290

[B118] Gaman A. M. Uzoni A. Popa-Wagner A. Andrei A. Petcu E. B. (2016). The Role of Oxidative Stress in Etiopathogenesis of Chemotherapy Induced Cognitive Impairment (CICI)-"Chemobrain". Aging Dis. 7, 307–317. 10.14336/AD.2015.1022 27330845 PMC4898927

[B119] Gao Y. Tang Y. Zhang H. Chu X. Yan B. Li J. (2021). Vincristine Leads to Colonic Myenteric Neurons Injury via Pro-inflammatory Macrophages Activation. Biochem. Pharmacol. 186, 114479. 10.1016/j.bcp.2021.114479 33617842

[B120] Geisler S. Doan R. A. Cheng G. C. Cetinkaya-Fisgin A. Huang S. X. Höke A. (2019). Vincristine and Bortezomib Use Distinct Upstream Mechanisms to Activate a Common SARM1-dependent Axon Degeneration Program. JCI Insight 4 (5), E129920. 10.1172/jci.insight.129920 PMC6777905 31484833

[B121] Geisler S. (2021). Vincristine- and Bortezomib-Induced Neuropathies - from Bedside to Bench and Back. Exp. Neurol. 336, 113519. 10.1016/j.expneurol.2020.113519 33129841 PMC11160556

[B122] Geraghty A. C. Gibson E. M. Ghanem R. A. Greene J. J. Ocampo A. Goldstein A. K. (2019). Loss of Adaptive Myelination Contributes to Methotrexate Chemotherapy-Related Cognitive Impairment. Neuron 103, 250–e8. 10.1016/j.neuron.2019.04.032 31122677 PMC6697075

[B123] Gewirtz D. A. (2014). Autophagy and Senescence in Cancer Therapy. J. Cell Physiol 229, 6–9. 10.1002/jcp.24420 23794221

[B124] Gibson E. M. Nagaraja S. Ocampo A. Tam L. T. Wood L. S. Pallegar P. N. (2019). Methotrexate Chemotherapy Induces Persistent Tri-glial Dysregulation that Underlies Chemotherapy-Related Cognitive Impairment. Cell 176, 43–e13. 10.1016/j.cell.2018.10.049 30528430 PMC6329664

[B125] Glasmacher A. Hahn C. Hoffmann F. Naumann R. Goldschmidt H. Von Lilienfeld-Toal M. (2006). A Systematic Review of Phase-II Trials of Thalidomide Monotherapy in Patients with Relapsed or Refractory Multiple Myeloma. Br. J. Haematol. 132, 584–593. 10.1111/j.1365-2141.2005.05914.x 16445831

[B126] Glass C. K. Saijo K. Winner B. Marchetto M. C. Gage F. H. (2010). Mechanisms Underlying Inflammation in Neurodegeneration. Cell 140, 918–934. 10.1016/j.cell.2010.02.016 20303880 PMC2873093

[B127] Goldman S. A. Chen Z. (2011). Perivascular Instruction of Cell Genesis and Fate in the Adult Brain. Nat. Neurosci. 14, 1382–1389. 10.1038/nn.2963 22030549 PMC3655803

[B128] Goode E. L. Ulrich C. M. Potter J. D. (2002). Polymorphisms in DNA Repair Genes and Associations with Cancer Risk. Cancer Epidemiol. Biomarkers Prev. 11, 1513–1530. 12496039

[B129] Gornstein E. Schwarz T. L. (2014). The Paradox of Paclitaxel Neurotoxicity: Mechanisms and Unanswered Questions. Neuropharmacology 76 Pt A, 175–183. 10.1016/j.neuropharm.2013.08.016 23978385

[B130] Gornstein E. L. Schwarz T. L. (2017). Neurotoxic Mechanisms of Paclitaxel Are Local to the Distal Axon and Independent of Transport Defects. Exp. Neurol. 288, 153–166. 10.1016/j.expneurol.2016.11.015 27894788 PMC5568627

[B131] Groves T. R. Farris R. Anderson J. E. Alexander T. C. Kiffer F. Carter G. (2017). 5-Fluorouracil Chemotherapy Upregulates Cytokines and Alters Hippocampal Dendritic Complexity in Aged Mice. Behav. Brain Res. 316, 215–224. 10.1016/j.bbr.2016.08.039 27599618 PMC5824640

[B132] Gu H. Wang C. Li J. Yang Y. Sun W. Jiang C. (2020). High Mobility Group Box-1-toll-like Receptor 4-phosphatidylinositol 3-kinase/protein Kinase B-Mediated Generation of Matrix Metalloproteinase-9 in the Dorsal Root Ganglion Promotes Chemotherapy-Induced Peripheral Neuropathy. Int. J. Cancer 146, 2810–2821. 10.1002/ijc.32652 31465111

[B133] Gutmann D. H. (2019). Clearing the Fog Surrounding Chemobrain. Cell 176, 2–4. 10.1016/j.cell.2018.12.027 30633904

[B134] Hagiwara H. Sunada Y. (2004). Mechanism of Taxane Neurotoxicity. Breast Cancer 11, 82–85. 10.1007/BF02968008 14718798

[B135] Han R. Yang Y. M. Dietrich J. Luebke A. Mayer-Pröschel M. Noble M. (2008). Systemic 5-fluorouracil Treatment Causes a Syndrome of Delayed Myelin Destruction in the central Nervous System. J. Biol. 7, 12. 10.1186/jbiol69 18430259 PMC2397490

[B136] Hawthorn J. Ostler K. J. Andrews P. L. (1988). The Role of the Abdominal Visceral Innervation and 5-hydroxytryptamine M-Receptors in Vomiting Induced by the Cytotoxic Drugs Cyclophosphamide and Cis-Platin in the Ferret. Q. J. Exp. Physiol. 73, 7–21. 10.1113/expphysiol.1988.sp003124 3347698

[B137] Hermelink K. Küchenhoff H. Untch M. Bauerfeind I. Lux M. P. Bühner M. (2010). Two Different Sides of 'chemobrain': Determinants and Nondeterminants of Self-Perceived Cognitive Dysfunction in a Prospective, Randomized, Multicenter Study. Psychooncology 19, 1321–1328. 10.1002/pon.1695 20127909

[B138] Hesketh P. J. Kris M. G. Basch E. Bohlke K. Barbour S. Y. Clark-Snow R. A. (2020). Antiemetics: ASCO Guideline Update. J. Clin. Oncol. 38, 2782–2797. 10.1200/JCO.20.01296 32658626

[B139] Hobson R. W. Jervis H. R. Kingry R. L. Wallace J. R. (1974). Small Bowel Changes Associated with Vincristine Sulfate Treatment: an Experimental Study in the guinea Pig. Cancer 34, 1888–1896. 10.1002/1097-0142(197412)34:6<1888::aid-cncr2820340606>3.0.co;2-5 4434323

[B140] Hoeffner E. G. (2016). Central Nervous System Complications of Oncologic Therapy. Hematol. Oncol. Clin. North. Am. 30, 899–920. 10.1016/j.hoc.2016.03.010 27444003

[B141] Hoeijmakers J. H. (2009). DNA Damage, Aging, and Cancer. N. Engl. J. Med. 361, 1475–1485. 10.1056/NEJMra0804615 19812404

[B142] Horn C. C. Kimball B. A. Wang H. Kaus J. Dienel S. Nagy A. (2013). Why Can't Rodents Vomit? A Comparative Behavioral, Anatomical, and Physiological Study. PLoS One 8, e60537. 10.1371/journal.pone.0060537 23593236 PMC3622671

[B143] Horowitz T. S. Suls J. Treviño M. (2018). A Call for a Neuroscience Approach to Cancer-Related Cognitive Impairment. Trends Neurosci. 41, 493–496. 10.1016/j.tins.2018.05.001 29907436

[B144] Huang Z. Z. Li D. Liu C. C. Cui Y. Zhu H. Q. Zhang W. W. (2014). CX3CL1-mediated Macrophage Activation Contributed to Paclitaxel-Induced DRG Neuronal Apoptosis and Painful Peripheral Neuropathy. Brain Behav. Immun. 40, 155–165. 10.1016/j.bbi.2014.03.014 24681252

[B145] Hyrien O. Dietrich J. Noble M. (2010). Mathematical and Experimental Approaches to Identify and Predict the Effects of Chemotherapy on Neuroglial Precursors. Cancer Res. 70, 10051–10059. 10.1158/0008-5472.CAN-10-1400 21056994 PMC3035395

[B146] Illias A. M. Gist A. C. Zhang H. Kosturakis A. K. Dougherty P. M. (2018). Chemokine CCL2 and its Receptor CCR2 in the Dorsal Root Ganglion Contribute to Oxaliplatin-Induced Mechanical Hypersensitivity. Pain 159, 1308–1316. 10.1097/j.pain.0000000000001212 29554018 PMC6008166

[B147] Jarmolowicz D. P. Gehringer R. Lemley S. M. Sofis M. J. Kaplan S. Johnson M. A. (2019). 5-Fluorouracil Impairs Attention and Dopamine Release in Rats. Behav. Brain Res. 362, 319–322. 10.1016/j.bbr.2019.01.007 30630020 PMC6830570

[B148] Jessen K. R. (2004). Glial Cells. Int. J. Biochem. Cell Biol 36, 1861–1867. 10.1016/j.biocel.2004.02.023 15203098

[B149] Johnston K. D. Lu Z. Rudd J. A. (2014). Looking beyond 5-HT(3) Receptors: a Review of the Wider Role of Serotonin in the Pharmacology of Nausea and Vomiting. Eur. J. Pharmacol. 722, 13–25. 10.1016/j.ejphar.2013.10.014 24189639

[B150] Johnstone T. C. Suntharalingam K. Lippard S. J. (2016). The Next Generation of Platinum Drugs: Targeted Pt(II) Agents, Nanoparticle Delivery, and Pt(IV) Prodrugs. Chem. Rev. 116, 3436–3486. 10.1021/acs.chemrev.5b00597 26865551 PMC4792284

[B151] Joshi G. Aluise C. D. Cole M. P. Sultana R. Pierce W. M. Vore M. (2010). Alterations in Brain Antioxidant Enzymes and Redox Proteomic Identification of Oxidized Brain Proteins Induced by the Anti-cancer Drug Adriamycin: Implications for Oxidative Stress-Mediated Chemobrain. Neuroscience 166, 796–807. 10.1016/j.neuroscience.2010.01.021 20096337 PMC2852883

[B152] Joshi G. Hardas S. Sultana R. St Clair D. K. Vore M. Butterfield D. A. (2007). Glutathione Elevation by Gamma-Glutamyl Cysteine Ethyl Ester as a Potential Therapeutic Strategy for Preventing Oxidative Stress in Brain Mediated by *In Vivo* Administration of Adriamycin: Implication for Chemobrain. J. Neurosci. Res. 85, 497–503. 10.1002/jnr.21158 17171703

[B153] Kang S. Lee S. Kim J. Kim J. C. Kim S. H. Son Y. (2018). Chronic Treatment with Combined Chemotherapeutic Agents Affects Hippocampal Micromorphometry and Function in Mice, Independently of Neuroinflammation. Exp. Neurobiol. 27, 419–436. 10.5607/en.2018.27.5.419 30429651 PMC6221841

[B154] Kaplan S. V. Limbocker R. A. Gehringer R. C. Divis J. L. Osterhaus G. L. Newby M. D. (2016). Impaired Brain Dopamine and Serotonin Release and Uptake in Wistar Rats Following Treatment with Carboplatin. ACS Chem. Neurosci. 7, 689–699. 10.1021/acschemneuro.5b00029 27145395 PMC4911621

[B155] Kashyap P. Farrugia G. (2011). Oxidative Stress: Key Player in Gastrointestinal Complications of Diabetes. Neurogastroenterol Motil. 23 (2), 111–114. 10.1111/j.1365-2982.2010.01659.x 21226884 PMC4441525

[B156] Keefe D. M. Elting L. S. Nguyen H. T. Grunberg S. M. Aprile G. Bonaventura A. (2014). Risk and Outcomes of Chemotherapy-Induced Diarrhea (CID) Among Patients with Colorectal Cancer Receiving Multi-Cycle Chemotherapy. Cancer Chemother. Pharmacol. 74, 675–680. 10.1007/s00280-014-2526-5 25055935

[B157] Keefe D. M. Gibson R. J. Hauer-Jensen M. (2004). Gastrointestinal Mucositis. Semin. Oncol. Nurs. 20, 38–47. 10.1053/j.soncn.2003.10.007 15038516

[B158] Keeney J. T. R. Ren X. Warrier G. Noel T. Powell D. K. Brelsfoard J. M. (2018). Doxorubicin-induced Elevated Oxidative Stress and Neurochemical Alterations in Brain and Cognitive Decline: protection by MESNA and Insights into Mechanisms of Chemotherapy-Induced Cognitive Impairment ("chemobrain"). Oncotarget 9, 30324–30339. 10.18632/oncotarget.25718 30100992 PMC6084398

[B159] Keifer J. A. Guttridge D. C. Ashburner B. P. Baldwin A. S. (2001). Inhibition of NF-Kappa B Activity by Thalidomide through Suppression of IkappaB Kinase Activity. J. Biol. Chem. 276, 22382–22387. 10.1074/jbc.M100938200 11297551

[B160] Kerb R. (2006). Implications of Genetic Polymorphisms in Drug Transporters for Pharmacotherapy. Cancer Lett. 234, 4–33. 10.1016/j.canlet.2005.06.051 16504381

[B161] Klein S. Seidler B. Kettenberger A. Sibaev A. Rohn M. Feil R. (2013). Interstitial Cells of Cajal Integrate Excitatory and Inhibitory Neurotransmission with Intestinal Slow-Wave Activity. Nat. Commun. 4, 1630. 10.1038/ncomms2626 23535651

[B162] Koth S. M. Kolesar J. (2017). New Options and Controversies in the Management of Chemotherapy-Induced Nausea and Vomiting. Am. J. Health Syst. Pharm. 74, 812–819. 10.2146/ajhp160227 28396308

[B163] Koźmiński P. Halik P. K. Chesori R. Gniazdowska E. (2020). Overview of Dual-Acting Drug Methotrexate in Different Neurological Diseases, Autoimmune Pathologies and Cancers. Int. J. Mol. Sci. 21, 3483. 10.3390/ijms21103483 PMC7279024 32423175

[B164] Kronfol Z. Remick D. G. (2000). Cytokines and the Brain: Implications for Clinical Psychiatry. Am. J. Psychiatry 157, 683–694. 10.1176/appi.ajp.157.5.683 10784457

[B165] Landowski T. H. Megli C. J. Nullmeyer K. D. Lynch R. M. Dorr R. T. (2005). Mitochondrial-mediated Disregulation of Ca2+ Is a Critical Determinant of Velcade (PS-341/Bortezomib) Cytotoxicity in Myeloma Cell Lines. Cancer Res. 65, 3828–3836. 10.1158/0008-5472.CAN-04-3684 15867381

[B166] Lang I. M. (1990). Digestive Tract Motor Correlates of Vomiting and Nausea. Can. J. Physiol. Pharmacol. 68, 242–253. 10.1139/y90-038 2178749

[B167] Lange M. Joly F. Vardy J. Ahles T. Dubois M. Tron L. (2019). Cancer-related Cognitive Impairment: an Update on State of the Art, Detection, and Management Strategies in Cancer Survivors. Ann. Oncol. 30, 1925–1940. 10.1093/annonc/mdz410 31617564 PMC8109411

[B168] Lee M. Y. Ha S. E. Park C. Park P. J. Fuchs R. Wei L. (2017). Transcriptome of Interstitial Cells of Cajal Reveals Unique and Selective Gene Signatures. PLoS One 12, e0176031. 10.1371/journal.pone.0176031 28426719 PMC5398589

[B169] Lehky T. J. Leonard G. D. Wilson R. H. Grem J. L. Floeter M. K. (2004). Oxaliplatin-induced Neurotoxicity: Acute Hyperexcitability and Chronic Neuropathy. Muscle Nerve 29, 387–392. 10.1002/mus.10559 14981738

[B170] Li D. Huang Z. Z. Ling Y. Z. Wei J. Y. Cui Y. Zhang X. Z. (2015). Up-regulation of CX3CL1 via Nuclear Factor-κb-dependent Histone Acetylation Is Involved in Paclitaxel-Induced Peripheral Neuropathy. Anesthesiology 122, 1142–1151. 10.1097/ALN.0000000000000560 25494456

[B171] Lin Y. T. Seo J. Gao F. Feldman H. M. Wen H. L. Penney J. (2018). APOE4 Causes Widespread Molecular and Cellular Alterations Associated with Alzheimer's Disease Phenotypes in Human iPSC-Derived Brain Cell Types. Neuron 98, 1141–e7. 10.1016/j.neuron.2018.05.008 29861287 PMC6023751

[B172] Liu C. Luan S. OuYang H. Huang Z. Wu S. Ma C. (2016). Upregulation of CCL2 via ATF3/c-Jun Interaction Mediated the Bortezomib-Induced Peripheral Neuropathy. Brain Behav. Immun. 53, 96–104. 10.1016/j.bbi.2015.11.004 26554515

[B173] Liu D. Sun M. Xu D. Ma X. Gao D. Yu H. (2019). Inhibition of TRPA1 and IL-6 Signal Alleviates Neuropathic Pain Following Chemotherapeutic Bortezomib. Physiol. Res. 68, 845–855. 10.33549/physiolres.934015 31424261

[B174] Lobert S. Vulevic B. Correia J. J. (1996). Interaction of vinca Alkaloids with Tubulin: A Comparison of Vinblastine, Vincristine, and Vinorelbine. Biochemistry 35, 6806–6814. 10.1021/bi953037i 8639632

[B175] Lomeli N. Di K. Czerniawski J. Guzowski J. F. Bota D. A. (2017). Cisplatin-induced Mitochondrial Dysfunction Is Associated with Impaired Cognitive Function in Rats. Free Radic. Biol. Med. 102, 274–286. 10.1016/j.freeradbiomed.2016.11.046 27908784 PMC5308450

[B176] López-Gómez L. Díaz-Ruano S. Girón R. López-Pérez A. E. Vera G. Herradón Pliego E. (2018). Preclinical Evaluation of the Effects on the Gastrointestinal Tract of the Antineoplastic Drug Vincristine Repeatedly Administered to Rats. Neurogastroenterol Motil. 30, e13399. 29971865 10.1111/nmo.13399

[B177] Lu P. H. Lee G. J. Raven E. P. Tingus K. Khoo T. Thompson P. M. (2011). Age-related Slowing in Cognitive Processing Speed Is Associated with Myelin Integrity in a Very Healthy Elderly Sample. J. Clin. Exp. Neuropsychol. 33, 1059–1068. 10.1080/13803395.2011.595397 22133139 PMC3269444

[B178] Lu Z. Ngan M. P. Lin G. Yew D. T. W. Fan X. Andrews P. L. R. (2017). Gastric Myoelectric Activity during Cisplatin-Induced Acute and Delayed Emesis Reveals a Temporal Impairment of Slow Waves in Ferrets: Effects Not Reversed by the GLP-1 Receptor Antagonist, Exendin (9-39). Oncotarget 8, 98691–98707. 10.18632/oncotarget.21859 29228720 PMC5716760

[B179] Lucchetta M. Lonardi S. Bergamo F. Alberti P. Velasco R. Argyriou A. A. (2012). Incidence of Atypical Acute Nerve Hyperexcitability Symptoms in Oxaliplatin-Treated Patients with Colorectal Cancer. Cancer Chemother. Pharmacol. 70, 899–902. 10.1007/s00280-012-2006-8 23108696

[B180] Luo X. Huh Y. Bang S. He Q. Zhang L. Matsuda M. (2019). Macrophage Toll-like Receptor 9 Contributes to Chemotherapy-Induced Neuropathic Pain in Male Mice. J. Neurosci. 39, 6848–6864. 10.1523/JNEUROSCI.3257-18.2019 31270160 PMC6733562

[B181] Ma J. Huo X. Jarpe M. B. Kavelaars A. Heijnen C. J. (2018). Pharmacological Inhibition of HDAC6 Reverses Cognitive Impairment and Tau Pathology as a Result of Cisplatin Treatment. Acta Neuropathol. Commun. 6, 103. 10.1186/s40478-018-0604-3 30270813 PMC6166273

[B182] MacDougall M. R. Sharma S. (2021). “Physiology, Chemoreceptor Trigger Zone,” in StatPearls [Internet] (Treasure Island (FL): StatPearls Publishing). 30725818

[B183] Magge R. S. Deangelis L. M. (2015). The Double-Edged Sword: Neurotoxicity of Chemotherapy. Blood Rev. 29, 93–100. 10.1016/j.blre.2014.09.012 25445718 PMC5944623

[B184] Makale M. T. King G. L. (1992). Surgical and Pharmacological Dissociation of Cardiovascular and Emetic Responses to Intragastric CuSO4. Am. J. Physiol. 263, R284–R291. 10.1152/ajpregu.1992.263.2.R284 1354943

[B185] Mandelblatt J. S. Small B. J. Luta G. Hurria A. Jim H. Mcdonald B. C. (2018). Cancer-Related Cognitive Outcomes Among Older Breast Cancer Survivors in the Thinking and Living with Cancer Study. J. Clin. Oncol. 3 (36), JCO1800140. 10.1200/JCO.18.00140 PMC7237199 30281396

[B186] Manjavachi M. N. Passos G. F. Trevisan G. Araújo S. B. Pontes J. P. Fernandes E. S. (2019). Spinal Blockage of CXCL1 and its Receptor CXCR2 Inhibits Paclitaxel-Induced Peripheral Neuropathy in Mice. Neuropharmacology 151, 136–143. 10.1016/j.neuropharm.2019.04.014 30991054

[B187] Matsos A. Johnston I. N. (2019). Chemotherapy-induced Cognitive Impairments: A Systematic Review of the Animal Literature. Neurosci. Biobehav Rev. 102, 382–399. 10.1016/j.neubiorev.2019.05.001 31063740

[B188] Maynard S. Fang E. F. Scheibye-Knudsen M. Croteau D. L. Bohr V. A. (2015). DNA Damage, DNA Repair, Aging, and Neurodegeneration. Cold Spring Harb Perspect. Med. 5, a025130. 10.1101/cshperspect.a025130 26385091 PMC4588127

[B189] McKenzie I. A. Ohayon D. Li H. De Faria J. P. Emery B. Tohyama K. (2014). Motor Skill Learning Requires Active central Myelination. Science 346, 318–322. 10.1126/science.1254960 25324381 PMC6324726

[B190] McQuade R. M. Al Thaalibi M. Nurgali K. (2020). Impact of Chemotherapy-Induced Enteric Nervous System Toxicity on Gastrointestinal Mucositis. Curr. Opin. Support. Palliat. Care 14, 293–300. 10.1097/SPC.0000000000000515 32769620

[B191] McQuade R. M. Carbone S. E. Stojanovska V. Rahman A. Gwynne R. M. Robinson A. M. (2016c). Role of Oxidative Stress in Oxaliplatin-Induced Enteric Neuropathy and Colonic Dysmotility in Mice. Br. J. Pharmacol. 173, 3502–3521. 10.1111/bph.13646 27714760 PMC5120153

[B192] McQuade R. M. Stojanovska V. Abalo R. Bornstein J. C. Nurgali K. (2016a). Chemotherapy-induced Constipation and Diarrhea: Pathophysiology, Current and Emerging Treatments. Front. Pharmacol. 7, 414. 10.3389/fphar.2016.00414 27857691 PMC5093116

[B193] McQuade R. M. Stojanovska V. Bornstein J. C. Nurgali K. (2017a). Colorectal Cancer Chemotherapy: the Evolution of Treatment and New Approaches. Curr. Med. Chem. 24, 1537–1557. 10.2174/0929867324666170111152436 28079003

[B194] McQuade R. M. Stojanovska V. Donald E. Abalo R. Bornstein J. C. Nurgali K. (2016b). Gastrointestinal Dysfunction and Enteric Neurotoxicity Following Treatment with Anticancer Chemotherapeutic Agent 5-fluorouracil. Neurogastroenterol Motil. 28, 1861–1875. 10.1111/nmo.12890 27353132

[B195] McQuade R. M. Stojanovska V. Donald E. L. Rahman A. A. Campelj D. G. Abalo R. (2017b). Irinotecan-induced Gastrointestinal Dysfunction Is Associated with Enteric Neuropathy, but Increased Numbers of Cholinergic Myenteric Neurons. Front. Physiol. 8, 391. 10.3389/fphys.2017.00391 28642718 PMC5462962

[B196] McQuade R. M. Stojanovska V. Stavely R. Timpani C. Petersen A. C. Abalo R. (2018). Oxaliplatin-induced Enteric Neuronal Loss and Intestinal Dysfunction Is Prevented by Co-treatment with BGP-15. Br. J. Pharmacol. 175, 656–677. 10.1111/bph.14114 29194564 PMC5786462

[B197] McQuade R. M. Bornstein J. C. Nurgali K. (2014). Anti-colorectal Cancer Chemotherapy-Induced Diarrhoea: Current Treatments and Side-Effects. Ijcm 05, 393–406. 10.4236/ijcm.2014.57054

[B198] Meethal S. V. Hogan K. J. Mayanil C. S. Iskandar B. J. (2013). Folate and Epigenetic Mechanisms in Neural Tube Development and Defects. Childs Nerv Syst. 29, 1427–1433. 10.1007/s00381-013-2162-0 24013316

[B199] Melchior M. Juif P. E. Gazzo G. Petit-Demoulière N. Chavant V. Lacaud A. (2018). Pharmacological rescue of Nociceptive Hypersensitivity and Oxytocin Analgesia Impairment in a Rat Model of Neonatal Maternal Separation. Pain 159, 2630–2640. 10.1097/j.pain.0000000000001375 30169420

[B200] Menzin A. W. King S. A. Aikins J. K. Mikuta J. J. Rubin S. C. (1994). Taxol (Paclitaxel) Was Approved by FDA for the Treatment of Patients with Recurrent Ovarian Cancer. Gynecol. Oncol. 54, 103. 7912683

[B201] Meregalli C. Canta A. Carozzi V. A. Chiorazzi A. Oggioni N. Gilardini A. (2010). Bortezomib-induced Painful Neuropathy in Rats: A Behavioral, Neurophysiological and Pathological Study in Rats. Eur. J. Pain 14, 343–350. 10.1016/j.ejpain.2009.07.001 19695912

[B202] Meregalli C. Marjanovic I. Scali C. Monza L. Spinoni N. Galliani C. (2018). High-dose Intravenous Immunoglobulins Reduce Nerve Macrophage Infiltration and the Severity of Bortezomib-Induced Peripheral Neurotoxicity in Rats. J. Neuroinflammation 15, 232. 10.1186/s12974-018-1270-x 30131066 PMC6103882

[B203] Michán S. Li Y. Chou M. M. Parrella E. Ge H. Long J. M. (2010). SIRT1 Is Essential for normal Cognitive Function and Synaptic Plasticity. J. Neurosci. 30, 9695–9707. 10.1523/JNEUROSCI.0027-10.2010 20660252 PMC2921958

[B204] Michaud M. Balardy L. Moulis G. Gaudin C. Peyrot C. Vellas B. (2013). Proinflammatory Cytokines, Aging, and Age-Related Diseases. J. Am. Med. Dir. Assoc. 14, 877–882. 10.1016/j.jamda.2013.05.009 23792036

[B205] Mihlon F. Ray C. E. Jr. Messersmith W. (2010). Chemotherapy Agents: a Primer for the Interventional Radiologist. Semin. Intervent Radiol. 27, 384–390. 10.1055/s-0030-1267852 22550380 PMC3324210

[B206] Miller K. D. Nogueira L. Mariotto A. B. Rowland J. H. Yabroff K. R. Alfano C. M. (2019). Cancer Treatment and Survivorship Statistics, 2019. CA Cancer J. Clin. 69, 363–385. 10.3322/caac.21565 31184787

[B207] Minami M. Ogawa T. Endo T. Hamaue N. Hirafuji M. Yoshioka M. (1997). Cyclophosphamide Increases 5-hydroxytryptamine Release from the Isolated Ileum of the Rat. Res. Commun. Mol. Pathol. Pharmacol. 97, 13–24. 9507564

[B208] Ming G. L. Song H. (2011). Adult Neurogenesis in the Mammalian Brain: Significant Answers and Significant Questions. Neuron 70, 687–702. 10.1016/j.neuron.2011.05.001 21609825 PMC3106107

[B209] Minton O. Stone P. C. (2012). A Comparison of Cognitive Function, Sleep and Activity Levels in Disease-free Breast Cancer Patients with or without Cancer-Related Fatigue Syndrome. BMJ Support. Palliat. Care 2, 231–238. 10.1136/bmjspcare-2011-000172 PMC3621525 23585925

[B210] Mirzayans R. Andrais B. Murray D. (2018). Roles of Polyploid/Multinucleated Giant Cancer Cells in Metastasis and Disease Relapse Following Anticancer Treatment. Cancers (Basel) 10, 118. 10.3390/cancers10040118 PMC5923373 29662021

[B211] Miskowiak K. W. Kjaerstad H. L. Støttrup M. M. Svendsen A. M. Demant K. M. Hoeffding L. K. (2017). The Catechol-O-Methyltransferase (COMT) Val158Met Genotype Modulates Working Memory-Related Dorsolateral Prefrontal Response and Performance in Bipolar Disorder. Bipolar Disord. 19, 214–224. 10.1111/bdi.12497 28544426

[B212] Mitchell E. P. (2006). Gastrointestinal Toxicity of Chemotherapeutic Agents. Semin. Oncol. 33, 106–120. 10.1053/j.seminoncol.2005.12.001 16473649

[B213] Mohammadi A. S. Li X. Ewing A. G. (2018). Mass Spectrometry Imaging Suggests that Cisplatin Affects Exocytotic Release by Alteration of Cell Membrane Lipids. Anal. Chem. 90, 8509–8516. 10.1021/acs.analchem.8b01395 29912552

[B214] Monje M. Dietrich J. (2012). Cognitive Side Effects of Cancer Therapy Demonstrate a Functional Role for Adult Neurogenesis. Behav. Brain Res. 227, 376–379. 10.1016/j.bbr.2011.05.012 21621557 PMC3221863

[B215] Montague K. Malcangio M. (2017). The Therapeutic Potential of Monocyte/macrophage Manipulation in the Treatment of Chemotherapy-Induced Painful Neuropathy. Front. Mol. Neurosci. 10, 397. 10.3389/fnmol.2017.00397 29230166 PMC5711788

[B216] Montague K. Simeoli R. Valente J. Malcangio M. (2018). A Novel Interaction between CX3CR1 and CCR2 Signalling in Monocytes Constitutes an Underlying Mechanism for Persistent Vincristine-Induced Pain. J. Neuroinflammation 15, 101. 10.1186/s12974-018-1116-6 29625610 PMC5889528

[B217] Moruno-Manchon J. F. Uzor N. E. Kesler S. R. Wefel J. S. Townley D. M. Nagaraja A. S. (2016). TFEB Ameliorates the Impairment of the Autophagy-Lysosome Pathway in Neurons Induced by Doxorubicin. Aging (Albany NY) 8, 3507–3519. 10.18632/aging.101144 27992857 PMC5270683

[B218] Mu L. Wang J. Cao B. Jelfs B. Chan R. H. Xu X. (2015). Impairment of Cognitive Function by Chemotherapy: Association with the Disruption of Phase-Locking and Synchronization in Anterior Cingulate Cortex. Mol. Brain 8, 32. 10.1186/s13041-015-0125-y 26001812 PMC4490721

[B219] Mustafa S. Walker A. Bennett G. Wigmore P. M. (2008). 5-Fluorouracil Chemotherapy Affects Spatial Working Memory and Newborn Neurons in the Adult Rat hippocampus. Eur. J. Neurosci. 28, 323–330. 10.1111/j.1460-9568.2008.06325.x 18702703

[B220] Myers J. S. Pierce J. Pazdernik T. (2008). Neurotoxicology of Chemotherapy in Relation to Cytokine Release, the Blood-Brain Barrier, and Cognitive Impairment. Oncol. Nurs. Forum 35, 916–920. 10.1188/08.ONF.916-920 18980922

[B221] Nadin S. B. Vargas-Roig L. M. Drago G. Ibarra J. Ciocca D. R. (2006). DNA Damage and Repair in Peripheral Blood Lymphocytes from Healthy Individuals and Cancer Patients: a Pilot Study on the Implications in the Clinical Response to Chemotherapy. Cancer Lett. 239, 84–97. 10.1016/j.canlet.2005.07.025 16143448

[B222] Nardini P. Pini A. Bessard A. Duchalais E. Niccolai E. Neunlist M. (2020). GLP-2 Prevents Neuronal and Glial Changes in the Distal colon of Mice Chronically Treated with Cisplatin. Int. J. Mol. Sci. 21, 8875. 10.3390/ijms21228875 PMC7700273 33238628

[B223] Nascimento F. P. Macedo-Júnior S. J. Borges F. R. Cremonese R. P. da Silva M. D. Luiz-Cerutti M. (2015). Thalidomide Reduces Mechanical Hyperalgesia and Depressive-like Behavior Induced by Peripheral Nerve Crush in Mice. Neuroscience 303, 51–58. 10.1016/j.neuroscience.2015.06.044 26126925

[B224] Nasu S. Misawa S. Nakaseko C. Shibuya K. Isose S. Sekiguchi Y. (2014). Bortezomib-induced Neuropathy: Axonal Membrane Depolarization Precedes Development of Neuropathy. Clin. Neurophysiol. 125, 381–387. 10.1016/j.clinph.2013.07.014 23973385

[B225] Newton H. B. (2012). Neurological Complications of Chemotherapy to the central Nervous System. Handb Clin. Neurol. 105, 903–916. 10.1016/B978-0-444-53502-3.00031-8 22230541

[B226] Ng T. Lee Y. Y. Chae J. W. Yeo A. H. L. Shwe M. Gan Y. X. (2017). Evaluation of Plasma Brain-Derived Neurotrophic Factor Levels and Self-Perceived Cognitive Impairment post-chemotherapy: a Longitudinal Study. BMC Cancer 17, 867. 10.1186/s12885-017-3861-9 29258453 PMC5735945

[B227] Ng T. Teo S. M. Yeo H. L. Shwe M. Gan Y. X. Cheung Y. T. (2016). Brain-derived Neurotrophic Factor Genetic Polymorphism (Rs6265) Is Protective against Chemotherapy-Associated Cognitive Impairment in Patients with Early-Stage Breast Cancer. Neuro Oncol. 18, 244–251. 10.1093/neuonc/nov162 26289590 PMC4724179

[B228] Nguyen L. D. Ehrlich B. E. (2020). Cellular Mechanisms and Treatments for Chemobrain: Insight from Aging and Neurodegenerative Diseases. EMBO Mol. Med. 12, e12075. 10.15252/emmm.202012075 32346964 PMC7278555

[B229] Nirschl J. J. Ghiretti A. E. Holzbaur E. L. F. (2017). The Impact of Cytoskeletal Organization on the Local Regulation of Neuronal Transport. Nat. Rev. Neurosci. 18, 585–597. 10.1038/nrn.2017.100 28855741 PMC6400490

[B230] Nishitsuji K. Hosono T. Nakamura T. Bu G. Michikawa M. (2011). Apolipoprotein E Regulates the Integrity of Tight Junctions in an Isoform-dependent Manner in an *In Vitro* Blood-Brain Barrier Model. J. Biol. Chem. 286, 17536–17542. 10.1074/jbc.M111.225532 21471207 PMC3093828

[B231] Niu N. Mercado-Uribe I. Liu J. (2017). Dedifferentiation into Blastomere-like Cancer Stem Cells via Formation of Polyploid Giant Cancer Cells. Oncogene 36, 4887–4900. 10.1038/onc.2017.72 28436947 PMC5582213

[B232] Nokia M. S. Anderson M. L. Shors T. J. (2012). Chemotherapy Disrupts Learning, Neurogenesis and Theta Activity in the Adult Brain. Eur. J. Neurosci. 36, 3521–3530. 10.1111/ejn.12007 23039863 PMC3523213

[B233] Numico G. Longo V. Courthod G. Silvestris N. (2015). Cancer Survivorship: Long-Term Side-Effects of Anticancer Treatments of Gastrointestinal Cancer. Curr. Opin. Oncol. 27, 351–357. 10.1097/CCO.0000000000000203 26049277

[B234] Oberste M. Schaffrath N. Schmidt K. Bloch W. Jäger E. Steindorf K. (2018). Protocol for the "Chemobrain in Motion - Study" (CIM - Study): a Randomized Placebo-Controlled Trial of the Impact of a High-Intensity Interval Endurance Training on Cancer Related Cognitive Impairments in Women with Breast Cancer Receiving First-Line Chemotherapy. BMC Cancer 18, 1071. 10.1186/s12885-018-4992-3 30400840 PMC6220507

[B235] Ogino M. H. Tadi P. (2021). Cyclophosphamide - *StatPearls* - NCBI Bookshelf. Available at: https://www.ncbi.nlm.nih.gov/books/NBK553087/ (Accessed July 20, 2021).

[B236] Old E. A. Nadkarni S. Grist J. Gentry C. Bevan S. Kim K. W. (2014). Monocytes Expressing CX3CR1 Orchestrate the Development of Vincristine-Induced Pain. J. Clin. Invest. 124, 2023–2036. 10.1172/JCI71389 24743146 PMC4001538

[B237] Olmos Y. Sánchez-Gómez F. J. Wild B. García-Quintans N. Cabezudo S. Lamas S. (2013). SirT1 Regulation of Antioxidant Genes Is Dependent on the Formation of a FoxO3a/PGC-1α Complex. Antioxid. Redox Signal. 19, 1507–1521. 10.1089/ars.2012.4713 23461683 PMC3797451

[B238] Omran M. Belcher E. K. Mohile N. A. Kesler S. R. Janelsins M. C. Hohmann A. G. (2021). Review of the Role of the Brain in Chemotherapy-Induced Peripheral Neuropathy. Front. Mol. Biosci. 8, 693133. 10.3389/fmolb.2021.693133 34179101 PMC8226121

[B239] Paintal A. S. (1973). Vagal Sensory Receptors and Their Reflex Effects. Physiol. Rev. 53, 159–227. 10.1152/physrev.1973.53.1.159 4568412

[B240] Paniagua N. Sánchez-Robles E. M. Bagues A. Martín-Fontelles M. I. Goicoechea C. Girón R. (2021). Behavior and Electrophysiology Studies of the Peripheral Neuropathy Induced by Individual and Co-administration of Paclitaxel and Oxaliplatin in Rat. Life Sci. 277, 119397. 10.1016/j.lfs.2021.119397 33794249

[B241] Park H. S. Kim C. J. Kwak H. B. No M. H. Heo J. W. Kim T. W. (2018). Physical Exercise Prevents Cognitive Impairment by Enhancing Hippocampal Neuroplasticity and Mitochondrial Function in Doxorubicin-Induced Chemobrain. Neuropharmacology 133, 451–461. 10.1016/j.neuropharm.2018.02.013 29477301

[B242] Pascual D. Goicoechea C. Suardíaz M. Martín M. I. (2005). A Cannabinoid Agonist, WIN 55,212-2, Reduces Neuropathic Nociception Induced by Paclitaxel in Rats. Pain 118, 23–34. 10.1016/j.pain.2005.07.008 16213089

[B243] Patel M. (2016). Targeting Oxidative Stress in Central Nervous System Disorders. Trends Pharmacol. Sci. 37, 768–778. 10.1016/j.tips.2016.06.007 27491897 PMC5333771

[B244] Peddi P. F. Peddi S. Santos E. S. Morgensztern D. (2014). Central Nervous System Toxicities of Chemotherapeutic Agents. Expert Rev. Anticancer Ther. 14, 857–863. 10.1586/14737140.2014.911089 24745349

[B245] Pellacani C. Eleftheriou G. (2020). Neurotoxicity of Antineoplastic Drugs: Mechanisms, Susceptibility, and Neuroprotective Strategies. Adv. Med. Sci. 65, 265–285. 10.1016/j.advms.2020.04.001 32361484

[B246] Percie du Sert N. Chu K. M. Wai M. K. Rudd J. A. Andrews P. L. (2009a). Reduced Normogastric Electrical Activity Associated with Emesis: a Telemetric Study in Ferrets. World J. Gastroenterol. 15, 6034–6043. 10.3748/wjg.15.6034 20027675 PMC2797659

[B247] Percie du Sert N. Chu K. M. Wai M. K. Rudd J. A. Andrews P. L. (2010). Telemetry in a Motion-Sickness Model Implicates the Abdominal Vagus in Motion-Induced Gastric Dysrhythmia. Exp. Physiol. 95, 768–773. 10.1113/expphysiol.2009.052001 20360423

[B248] Percie du Sert N. Rudd J. A. Moss R. Andrews P. L. (2009b). The Delayed Phase of Cisplatin-Induced Emesis Is Mediated by the Area Postrema and Not the Abdominal Visceral Innervation in the Ferret. Neurosci. Lett. 465, 16–20. 10.1016/j.neulet.2009.08.075 19733218

[B249] Peters C. M. Jimenez-Andrade J. M. Jonas B. M. Sevcik M. A. Koewler N. J. Ghilardi J. R. (2007). Intravenous Paclitaxel Administration in the Rat Induces a Peripheral Sensory Neuropathy Characterized by Macrophage Infiltration and Injury to Sensory Neurons and Their Supporting Cells. Exp. Neurol. 203, 42–54. 10.1016/j.expneurol.2006.07.022 17005179

[B250] Pini A. Garella R. Idrizaj E. Calosi L. Baccari M. C. Vannucchi M. G. (2016). Glucagon-like Peptide 2 Counteracts the Mucosal Damage and the Neuropathy Induced by Chronic Treatment with Cisplatin in the Mouse Gastric Fundus. Neurogastroenterol Motil. 28, 206–216. 10.1111/nmo.12712 26547262

[B251] Pitarokoili K. Yoon M. S. Kröger I. Reinacher-Schick A. Gold R. Schneider-Gold C. (2017). Severe Refractory CIDP: a Case Series of 10 Patients Treated with Bortezomib. J. Neurol. 264, 2010–2020. 10.1007/s00415-017-8599-4 28836002

[B252] Podratz J. L. Staff N. P. Froemel D. Wallner A. Wabnig F. Bieber A. J. (2011). *Drosophila melanogaster*: A New Model to Study Cisplatin-Induced Neurotoxicity. Neurobiol. Dis. 43, 330–337. 10.1016/j.nbd.2011.03.022 21514385 PMC3131093

[B253] Poruchynsky M. S. Sackett D. L. Robey R. W. Ward Y. Annunziata C. Fojo T. (2008). Proteasome Inhibitors Increase Tubulin Polymerization and Stabilization in Tissue Culture Cells: a Possible Mechanism Contributing to Peripheral Neuropathy and Cellular Toxicity Following Proteasome Inhibition. Cell Cycle 7, 940–949. 10.4161/cc.7.7.5625 18414063 PMC9416319

[B254] Pulito C. Cristaudo A. Porta C. Zapperi S. Blandino G. Morrone A. (2020). Oral Mucositis: the Hidden Side of Cancer Therapy. J. Exp. Clin. Cancer Res. 39, 210. 10.1186/s13046-020-01715-7 33028357 PMC7542970

[B255] Pullens M. J. De Vries J. Van Warmerdam L. J. Van De Wal M. A. Roukema J. A. (2013). Chemotherapy and Cognitive Complaints in Women with Breast Cancer. Psychooncology 22, 1783–1789. 10.1002/pon.3214 23109296

[B256] Pun P. B. Lu J. Moochhala S. (2009). Involvement of ROS in BBB Dysfunction. Free Radic. Res. 43, 348–364. 10.1080/10715760902751902 19241241

[B257] Quartu M. Carozzi V. A. Dorsey S. G. Serra M. P. Poddighe L. Picci C. (2014). Bortezomib Treatment Produces Nocifensive Behavior and Changes in the Expression of TRPV1, CGRP, and Substance P in the Rat DRG, Spinal Cord, and Sciatic Nerve. Biomed. Res. Int. 2014, 180428. 10.1155/2014/180428 24877063 PMC4022313

[B258] Rabik C. A. Dolan M. E. (2007). Molecular Mechanisms of Resistance and Toxicity Associated with Platinating Agents. Cancer Treat. Rev. 33, 9–23. 10.1016/j.ctrv.2006.09.006 17084534 PMC1855222

[B259] Rasmussen K. Benvenga M. J. Bymaster F. P. Calligaro D. O. Cohen I. R. Falcone J. F. (2005). Preclinical Pharmacology of FMPD [6-Fluoro-10-[3-(2-Methoxyethyl)-4-Methyl-Piperazin-1-Yl]-2-Methyl-4h-3-Thia-4,9-Diaza-Benzo[f]azulene]: a Potential Novel Antipsychotic with Lower Histamine H1 Receptor Affinity Than Olanzapine. J. Pharmacol. Exp. Ther. 315, 1265–1277. 10.1124/jpet.105.089326 16141369

[B260] Ravelli R. B. Gigant B. Curmi P. A. Jourdain I. Lachkar S. Sobel A. (2004). Insight into Tubulin Regulation from a Complex with Colchicine and a Stathmin-like Domain. Nature 428, 198–202. 10.1038/nature02393 15014504

[B261] Ren X. Boriero D. Chaiswing L. Bondada S. St Clair D. K. Butterfield D. A. (2019). Plausible Biochemical Mechanisms of Chemotherapy-Induced Cognitive Impairment ("chemobrain"), a Condition that Significantly Impairs the Quality of Life of many Cancer Survivors. Biochim. Biophys. Acta Mol. Basis Dis. 1865, 1088–1097. 10.1016/j.bbadis.2019.02.007 30759363 PMC6502692

[B262] Ren X. St Clair D. K. Butterfield D. A. (2017). Dysregulation of Cytokine Mediated Chemotherapy Induced Cognitive Impairment. Pharmacol. Res. 117, 267–273. 10.1016/j.phrs.2017.01.001 28063894

[B263] Ren Z. He H. Zuo Z. Xu Z. Wei Z. Deng J. (2019). The Role of Different SIRT1-Mediated Signaling Pathways in Toxic Injury. Cell Mol Biol Lett 24, 36. 10.1186/s11658-019-0158-9 31164908 PMC6543624

[B264] Riva B. Dionisi M. Potenzieri A. Chiorazzi A. Cordero-Sanchez C. Rigolio R. (2018). Oxaliplatin Induces pH Acidification in Dorsal Root Ganglia Neurons. Sci. Rep. 8, 15084. 10.1038/s41598-018-33508-6 30305703 PMC6180129

[B265] Robinson A. M. Stojanovska V. Rahman A. A. McQuade R. M. Senior P. V. Nurgali K. (2016). Effects of Oxaliplatin Treatment on the Enteric Glial Cells and Neurons in the Mouse Ileum. J. Histochem. Cytochem. 64, 530–545. 10.1369/0022155416656842 27389702 PMC5006136

[B266] Roila F. Molassiotis A. Herrstedt J. Aapro M. Gralla R. J. Bruera E. (2016). 2016 MASCC and ESMO Guideline Update for the Prevention of Chemotherapy- and Radiotherapy-Induced Nausea and Vomiting and of Nausea and Vomiting in Advanced Cancer Patients. Ann. Oncol. 27, v119–v133. 10.1093/annonc/mdw270 27664248

[B267] Rothenberg M. L. Meropol N. J. Poplin E. A. Van Cutsem E. Wadler S. (2001). Mortality Associated with Irinotecan Plus Bolus Fluorouracil/leucovorin: Summary Findings of an Independent Panel. J. Clin. Oncol. 19, 3801–3807. 10.1200/JCO.2001.19.18.3801 11559717

[B268] Rudd J. A. Jordan C. C. Naylor R. J. (1994). Profiles of Emetic Action of Cisplatin in the Ferret: a Potential Model of Acute and Delayed Emesis. Eur. J. Pharmacol. 262, R1–R2. 10.1016/0014-2999(94)90048-5 7813558

[B269] Rudd J. A. Nalivaiko E. Matsuki N. Wan C. Andrews P. L. (2015). The Involvement of TRPV1 in Emesis and Anti-emesis. Temperature (Austin) 2, 258–276. 10.1080/23328940.2015.1043042 27227028 PMC4843889

[B270] Rzeski W. Pruskil S. Macke A. Felderhoff-Mueser U. Reiher A. K. Hoerster F. (2004). Anticancer Agents Are Potent Neurotoxins *In Vitro* and *In Vivo* . Ann. Neurol. 56, 351–360. 10.1002/ana.20185 15349862

[B271] Saifee T. A. Elliott K. J. Rabin N. Yong K. L. D'Sa S. Brandner S. (2010). Bortezomib-induced Inflammatory Neuropathy. J. Peripher. Nerv Syst. 15, 366–368. 10.1111/j.1529-8027.2010.00287.x 21199108

[B272] Sam T. S. Cheng J. T. Johnston K. D. Kan K. K. Ngan M. P. Rudd J. A. (2003). Action of 5-HT3 Receptor Antagonists and Dexamethasone to Modify Cisplatin-Induced Emesis in Suncus Murinus (House Musk Shrew). Eur. J. Pharmacol. 472, 135–145. 10.1016/s0014-2999(03)01863-6 12860482

[B273] Schagen S. B. Boogerd W. Muller M. J. Huinink W. T. Moonen L. Meinhardt W. (2008). Cognitive Complaints and Cognitive Impairment Following BEP Chemotherapy in Patients with Testicular Cancer. Acta Oncol. 47, 63–70. 10.1080/02841860701518058 17934892

[B274] Schagen S. B. Klein M. Reijneveld J. C. Brain E. Deprez S. Joly F. (2014). Monitoring and Optimising Cognitive Function in Cancer Patients: Present Knowledge and Future Directions. EJC Suppl. 12, 29–40. 10.1016/j.ejcsup.2014.03.003 26217164 PMC4250534

[B275] Schänzer A. Wachs F. P. Wilhelm D. Acker T. Cooper-Kuhn C. Beck H. (2004). Direct Stimulation of Adult Neural Stem Cells *In Vitro* and Neurogenesis *In Vivo* by Vascular Endothelial Growth Factor. Brain Pathol. 14, 237–248. 10.1111/j.1750-3639.2004.tb00060.x 15446578 PMC8096047

[B276] Scherwath A. Schirmer L. Kruse M. Ernst G. Eder M. Dinkel A. (2013). Cognitive Functioning in Allogeneic Hematopoietic Stem Cell Transplantation Recipients and its Medical Correlates: a Prospective Multicenter Study. Psychooncology 22, 1509–1516. 10.1002/pon.3159 22945857

[B277] Schoch S. Gajewski S. Rothfuß J. Hartwig A. Köberle B. (2020). Comparative Study of the Mode of Action of Clinically Approved Platinum-Based Chemotherapeutics. Int. J. Mol. Sci. 21, 1–20. 10.3390/ijms21186928 PMC7555145 32967255

[B278] Schwörer H. Racké K. Kilbinger H. (1991). Cisplatin Increases the Release of 5-hydroxytryptamine (5-HT) from the Isolated Vascularly Perfused Small Intestine of the guinea-pig: Involvement of 5-HT3 Receptors. Naunyn Schmiedebergs Arch. Pharmacol. 344, 143–149. 10.1007/BF00167211 1719432

[B279] Seigers R. Schagen S. B. Beerling W. Boogerd W. Van Tellingen O. Van Dam F. S. (2008). Long-lasting Suppression of Hippocampal Cell Proliferation and Impaired Cognitive Performance by Methotrexate in the Rat. Behav. Brain Res. 186, 168–175. 10.1016/j.bbr.2007.08.004 17854921

[B280] Seigers R. Timmermans J. Van Der Horn H. J. De Vries E. F. Dierckx R. A. Visser L. (2010). Methotrexate Reduces Hippocampal Blood Vessel Density and Activates Microglia in Rats but Does Not Elevate central Cytokine Release. Behav. Brain Res. 207, 265–272. 10.1016/j.bbr.2009.10.009 19840821

[B281] Shaker F. H. El-Derany M. O. Wahdan S. A. El-Demerdash E. El-Mesallamy H. O. (2021). Berberine Ameliorates Doxorubicin-Induced Cognitive Impairment (Chemobrain) in Rats. Life Sci. 269, 119078. 10.1016/j.lfs.2021.119078 33460662

[B282] Shao Y. Tan B. Shi J. Zhou Q. (2019). Methotrexate Induces Astrocyte Apoptosis by Disrupting Folate Metabolism in the Mouse Juvenile central Nervous System. Toxicol. Lett. 301, 146–156. 10.1016/j.toxlet.2018.11.016 30502384

[B283] Sharma R. Tobin P. Clarke S. J. (2005). Management of Chemotherapy-Induced Nausea, Vomiting, Oral Mucositis, and Diarrhoea. Lancet Oncol. 6, 93–102. 10.1016/S1470-2045(05)01735-3 15683818

[B284] Sheldrick A. J. Krug A. Markov V. Leube D. Michel T. M. Zerres K. (2008). Effect of COMT Val158met Genotype on Cognition and Personality. Eur. Psychiatry 23, 385–389. 10.1016/j.eurpsy.2008.05.002 18755576

[B285] Shen S. Lim G. You Z. DIng W. Huang P. Ran C. (2017). Gut Microbiota Is Critical for the Induction of Chemotherapy-Induced Pain. Nat. Neurosci. 20, 1213–1216. 10.1038/nn.4606 28714953 PMC5575957

[B286] Shi D. D. Dong C. M. Ho L. C. Lam C. T. W. Zhou X. D. Wu E. X. (2018). Resveratrol, a Natural Polyphenol, Prevents Chemotherapy-Induced Cognitive Impairment: Involvement of Cytokine Modulation and Neuroprotection. Neurobiol. Dis. 114, 164–173. 10.1016/j.nbd.2018.03.006 29534932

[B287] Shi D. D. Huang Y. H. Lai C. S. W. Dong C. M. Ho L. C. Li X. Y. (2019a). Ginsenoside Rg1 Prevents Chemotherapy-Induced Cognitive Impairment: Associations with Microglia-Mediated Cytokines, Neuroinflammation, and Neuroplasticity. Mol. Neurobiol. 56, 5626–5642. 10.1007/s12035-019-1474-9 30659419

[B288] Shi D. D. Huang Y. H. Lai C. S. W. Dong C. M. Ho L. C. Wu E. X. (2019b). Chemotherapy-Induced Cognitive Impairment Is Associated with Cytokine Dysregulation and Disruptions in Neuroplasticity. Mol. Neurobiol. 56, 2234–2243. 10.1007/s12035-018-1224-4 30008071

[B289] Siau C. Xiao W. Bennett G. J. (2006). Paclitaxel- and Vincristine-Evoked Painful Peripheral Neuropathies: Loss of Epidermal Innervation and Activation of Langerhans Cells. Exp. Neurol. 201, 507–514. 10.1016/j.expneurol.2006.05.007 16797537 PMC1805691

[B290] Sikora E. Bielak-Zmijewska A. Dudkowska M. Krzystyniak A. Mosieniak G. Wesierska M. (2021). Cellular Senescence in Brain Aging. Front. Aging Neurosci. 13, 646924. 10.3389/fnagi.2021.646924 33732142 PMC7959760

[B291] Silva A. Wang Q. Wang M. Ravula S. K. Glass J. D. (2006). Evidence for Direct Axonal Toxicity in Vincristine Neuropathy. J. Peripher. Nerv Syst. 11, 211–216. 10.1111/j.1529-8027.2006.0090.x 16930282

[B292] Simó M. Gurtubay-Antolin A. Vaquero L. Bruna J. Rodríguez-Fornells A. (2018). Performance Monitoring in Lung Cancer Patients Pre- and post-chemotherapy Using fine-grained Electrophysiological Measures. Neuroimage Clin. 18, 86–96. 10.1016/j.nicl.2017.12.032 29387526 PMC5789765

[B293] Singh R. K. Kumar S. Prasad D. N. Bhardwaj T. R. (2018). Therapeutic Journery of Nitrogen Mustard as Alkylating Anticancer Agents: Historic to Future Perspectives. Eur. J. Med. Chem. 151, 401–433. 10.1016/j.ejmech.2018.04.001 29649739

[B294] Sliwinska M. A. Mosieniak G. Wolanin K. Babik A. Piwocka K. Magalska A. (2009). Induction of Senescence with Doxorubicin Leads to Increased Genomic Instability of HCT116 Cells. Mech. Ageing Dev. 130, 24–32. 10.1016/j.mad.2008.04.011 18538372

[B295] Small B. J. Rawson K. S. Walsh E. Jim H. S. Hughes T. F. Iser L. (2011). Catechol-O-methyltransferase Genotype Modulates Cancer Treatment-Related Cognitive Deficits in Breast Cancer Survivors. Cancer 117, 1369–1376. 10.1002/cncr.25685 21425136

[B296] Smith J. A. Das A. Ray S. K. Banik N. L. (2012). Role of Pro-inflammatory Cytokines Released from Microglia in Neurodegenerative Diseases. Brain Res. Bull. 87, 10–20. 10.1016/j.brainresbull.2011.10.004 22024597 PMC9827422

[B297] Sommariva S. Pongiglione B. Tarricone R. (2016). Impact of Chemotherapy-Induced Nausea and Vomiting on Health-Related Quality of Life and Resource Utilization: a Systematic Review. Crit. Rev. Oncol. Hematol. 99, 13–36. 10.1016/j.critrevonc.2015.12.001 26697988

[B298] Song S. J. Min J. Suh S. Y. Jung S. H. Hahn H. J. Im S. A. (2017). Incidence of Taxane-Induced Peripheral Neuropathy Receiving Treatment and Prescription Patterns in Patients with Breast Cancer. Support Care Cancer 25, 2241–2248. 10.1007/s00520-017-3631-x 28204996

[B299] Sordillo P. P. Sordillo L. A. (2020). The Mystery of Chemotherapy Brain: Kynurenines, Tubulin and Biophoton Release. Anticancer Res. 40, 1189–1200. 10.21873/anticanres.14061 32132016

[B300] Sougiannis A. T. VanderVeen B. N. Davis J. M. Fan D. Murphy E. A. (2021). Understanding Chemotherapy-Induced Intestinal Mucositis and Strategies to Improve Gut Resilience. Am. J. Physiol. Gastrointest. Liver Physiol. 320, G712–G719. 10.1152/ajpgi.00380.2020 33471628 PMC8202195

[B301] Speidell A. P. Demby T. Lee Y. Rodriguez O. Albanese C. Mandelblatt J. (2019). Development of a Human APOE Knock-In Mouse Model for Study of Cognitive Function after Cancer Chemotherapy. Neurotox Res. 35, 291–303. 10.1007/s12640-018-9954-7 30284204 PMC6333492

[B302] Spencer N. J. (2016). Motility Patterns in Mouse colon: Gastrointestinal Dysfunction Induced by Anticancer Chemotherapy. Neurogastroenterol Motil. 28, 1759–1764. 10.1111/nmo.12990 27891756

[B303] Staff N. P. Grisold A. Grisold W. Windebank A. J. (2017). Chemotherapy-induced Peripheral Neuropathy: A Current Review. Ann. Neurol. 81, 772–781. 10.1002/ana.24951 28486769 PMC5656281

[B304] Staurengo-Ferrari L. Green P. G. Araldi D. Ferrari L. F. Miaskowski C. Levine J. D. (2021). Sexual Dimorphism in the Contribution of Neuroendocrine Stress Axes to Oxaliplatin-Induced Painful Peripheral Neuropathy. Pain 162, 907–918. 10.1097/j.pain.0000000000002073 32947545 PMC7886966

[B305] Steadman P. E. Xia F. Ahmed M. Mocle A. J. Penning A. R. A. Geraghty A. C. (2020). Disruption of Oligodendrogenesis Impairs Memory Consolidation in Adult Mice. Neuron 105, 150–e6. 10.1016/j.neuron.2019.10.013 31753579 PMC7579726

[B306] Stern R. M. Koch K. L. Andrews P. L. R. (2011). Nausea: Mechanisms and Management. New York, U.S.A: Oxford University Press.

[B307] Stojanovska V. McQuade R. Rybalka E. Nurgali K. (2017). Neurotoxicity Associated with Platinum-Based Anti-cancer Agents: what Are the Implications of Copper Transporters? Curr. Med. Chem. 24, 1520–1536. 10.2174/0929867324666170112095428 28079002

[B308] Stojanovska V. McQuade R. M. Fraser S. Prakash M. Gondalia S. Stavely R. (2018a). Oxaliplatin-induced Changes in Microbiota, TLR4+ Cells and Enhanced HMGB1 Expression in the Murine colon. PLoS One 13, e0198359. 10.1371/journal.pone.0198359 29894476 PMC5997344

[B309] Stojanovska V. McQuade R. M. Miller S. Nurgali K. (2018b). Effects of Oxaliplatin Treatment on the Myenteric Plexus Innervation and Glia in the Murine Distal colon. J. Histochem. Cytochem. 66, 723–736. 10.1369/0022155418774755 29741434 PMC6158630

[B310] Stojanovska V. Sakkal S. Nurgali K. (2015). Platinum-based Chemotherapy: Gastrointestinal Immunomodulation and Enteric Nervous System Toxicity. Am. J. Physiol. Gastrointest. Liver Physiol. 308, G223–G232. 10.1152/ajpgi.00212.2014 25501548

[B311] Stone J. B. DeAngelis L. M. (2016). Cancer-treatment-induced Neurotoxicity-Ffocus on Newer Treatments. Nat. Rev. Clin. Oncol. 13, 92–105. 10.1038/nrclinonc.2015.152 26391778 PMC4979320

[B312] Stubblefield M. D. Burstein H. J. Burton A. W. Custodio C. M. Deng G. E. Ho M. (2009). NCCN Task Force Report: Management of Neuropathy in Cancer. J. Natl. Compr. Canc Netw. 7 (Suppl. 5), S1–S8. 10.6004/jnccn.2009.0078 19755042

[B313] Sundaram M. Guernsey D. L. Rajaraman M. M. Rajaraman R. (2004). Neosis: a Novel Type of Cell Division in Cancer. Cancer Biol. Ther. 3, 207–218. 10.4161/cbt.3.2.663 14726689

[B314] Sutherland A. Naessens K. Plugge E. Ware L. Head K. Burton M. J. (2018). Olanzapine for the Prevention and Treatment of Cancer-Related Nausea and Vomiting in Adults. Cochrane Database Syst. Rev. 9, CD012555. 10.1002/14651858.CD012555.pub2 30246876 PMC6513437

[B315] Ta L. E. Espeset L. Podratz J. Windebank A. J. (2006). Neurotoxicity of Oxaliplatin and Cisplatin for Dorsal Root Ganglion Neurons Correlates with Platinum-DNA Binding. Neurotoxicology 27, 992–1002. 10.1016/j.neuro.2006.04.010 16797073

[B316] Tacar O. Sriamornsak P. Dass C. R. (2013). Doxorubicin: an Update on Anticancer Molecular Action, Toxicity and Novel Drug Delivery Systems. J. Pharm. Pharmacol. 65, 157–170. 10.1111/j.2042-7158.2012.01567.x 23278683

[B317] Taillibert S. Le Rhun E. Chamberlain M. C. (2016). Chemotherapy-Related Neurotoxicity. Curr. Neurol. Neurosci. Rep. 16, 81. 10.1007/s11910-016-0686-x 27443648

[B318] Tamburin S. Park S. B. Alberti P. Demichelis C. Schenone A. Argyriou A. A. (2019). Taxane and Epothilone-Induced Peripheral Neurotoxicity: From Pathogenesis to Treatment. J. Peripher. Nerv Syst. 24, S40–S51. 10.1111/jns.12336 31647157

[B319] Tanaka F. Fukuse T. Wada H. Fukushima M. (2005). The History, Mechanism and Clinical Use of Oral 5-Fluorouracil Derivative Chemotherapeutic Agents. Curr. Pharm. Biotechnol. 1, 137–164. 10.2174/1389201003378979 11467334

[B320] Tangpong J. Cole M. P. Sultana R. Estus S. Vore M. St Clair W. (2007). Adriamycin-mediated Nitration of Manganese Superoxide Dismutase in the central Nervous System: Insight into the Mechanism of Chemobrain. J. Neurochem. 100, 191–201. 10.1111/j.1471-4159.2006.04179.x 17227439

[B321] Tangpong J. Cole M. P. Sultana R. Joshi G. Estus S. Vore M. (2006). Adriamycin-induced, TNF-Alpha-Mediated central Nervous System Toxicity. Neurobiol. Dis. 23, 127–139. 10.1016/j.nbd.2006.02.013 16697651

[B322] Tanihata S. Igarashi H. Suzuki M. Uchiyama T. (2000). Cisplatin-induced Early and Delayed Emesis in the pigeon. Br. J. Pharmacol. 130, 132–138. 10.1038/sj.bjp.0703283 10781008 PMC1572039

[B323] Thomas T. C. Beitchman J. A. Pomerleau F. Noel T. Jungsuwadee P. Butterfield D. A. (2017). Acute Treatment with Doxorubicin Affects Glutamate Neurotransmission in the Mouse Frontal Cortex and hippocampus. Brain Res. 1672, 10–17. 10.1016/j.brainres.2017.07.003 28705715 PMC5576558

[B324] Thorpe D. Butler R. Sultani M. Vanhoecke B. Stringer A. (2020). Irinotecan-induced Mucositis Is Associated with Goblet Cell Dysregulation and Neural Cell Damage in a Tumour Bearing DA Rat Model. Pathol. Oncol. Res. 26, 955–965. 10.1007/s12253-019-00644-x 30919275

[B325] Thorpe D. Stringer A. Butler R. (2013). Chemotherapy-induced Mucositis: the Role of Mucin Secretion and Regulation, and the Enteric Nervous System. Neurotoxicology 38, 101–105. 10.1016/j.neuro.2013.06.007 23827812

[B326] Toma W. Kyte S. L. Bagdas D. Alkhlaif Y. Alsharari S. D. Lichtman A. H. (2017). Effects of Paclitaxel on the Development of Neuropathy and Affective Behaviors in the Mouse. Neuropharmacology 117, 305–315. 10.1016/j.neuropharm.2017.02.020 28237807 PMC5489229

[B327] Triarico S. Romano A. Attinà G. Capozza M. A. Maurizi P. Mastrangelo S. (2021). Vincristine-Induced Peripheral Neuropathy (VIPN) in Pediatric Tumors: Mechanisms, Risk Factors, Strategies of Prevention and Treatment. Int. J. Mol. Sci. 22. 10.3390/ijms22084112 PMC8073828 33923421

[B328] Tsubota M. Fukuda R. Hayashi Y. Miyazaki T. Ueda S. Yamashita R. (2019). Role of Non-macrophage Cell-Derived HMGB1 in Oxaliplatin-Induced Peripheral Neuropathy and its Prevention by the Thrombin/thrombomodulin System in Rodents: Negative Impact of Anticoagulants. J. Neuroinflammation 16, 199. 10.1186/s12974-019-1581-6 31666085 PMC6822350

[B329] Tsujimoto S. Yanagimachi M. Tanoshima R. Urayama K. Y. Tanaka F. Aida N. (2016). Influence of ADORA2A Gene Polymorphism on Leukoencephalopathy Risk in MTX-Treated Pediatric Patients Affected by Hematological Malignancies. Pediatr. Blood Cancer 63, 1983–1989. 10.1002/pbc.26090 27399166

[B330] Tu L. Liu J. Y. H. Lu Z. Cui D. Ngan M. P. Du P. (2021). Insights into Acute and Delayed Cisplatin-Induced Emesis from a Microelectrode Array, Radiotelemetry and Whole-Body Plethysmography Study of *Suncus Murinus* (House Musk Shrew). Front. Pharmacol. 12, 746053. 10.3389/fphar.2021.746053 34925008 PMC8678571

[B331] Uranga J. A. García-Martínez J. M. García-Jiménez C. Vera G. Martín-Fontelles M. I. Abalo R. (2017). Alterations in the Small Intestinal wall and Motor Function after Repeated Cisplatin in Rat. Neurogastroenterol Motil. 29 (7). 10.1111/nmo.13047 28261911

[B332] Van Sebille Y. Z. Stansborough R. Wardill H. R. Bateman E. Gibson R. J. Keefe D. M. (2015). Management of Mucositis during Chemotherapy: from Pathophysiology to Pragmatic Therapeutics. Curr. Oncol. Rep. 17, 50. 10.1007/s11912-015-0474-9 26384312

[B333] Van Vuuren R. J. Visagie M. H. Theron A. E. Joubert A. M. (2015). Antimitotic Drugs in the Treatment of Cancer. Cancer Chemother. Pharmacol. 76, 1101–1112. 10.1007/s00280-015-2903-8 26563258 PMC4648954

[B334] Velasco R. Bruna J. (2015). Taxane-induced Peripheral Neurotoxicity. Toxics 3, 152–169. 10.3390/toxics3020152 29056655 PMC5634686

[B335] Vera G. Castillo M. Cabezos P. A. Chiarlone A. Martín M. I. Gori A. (2011). Enteric Neuropathy Evoked by Repeated Cisplatin in the Rat. Neurogastroenterol Motil. 23 (370-8), 370–373. 10.1111/j.1365-2982.2011.01674.x 21299719

[B336] Vera G. Chiarlone A. Cabezos P. A. Pascual D. Martín M. I. Abalo R. (2007). WIN 55,212-2 Prevents Mechanical Allodynia but Not Alterations in Feeding Behaviour Induced by Chronic Cisplatin in the Rat. Life Sci. 81, 468–479. 10.1016/j.lfs.2007.06.012 17673260

[B337] Vera G. Chiarlone A. Martín M. I. Abalo R. (2006). Altered Feeding Behaviour Induced by Long-Term Cisplatin in Rats. Auton. Neurosci. 126-127, 81–92. 10.1016/j.autneu.2006.02.011 16567130

[B338] Vera G. López-Pérez A. E. Uranga J. A. Girón R. Martín-Fontelles M. I. Abalo R. (2017). Involvement of Cannabinoid Signaling in Vincristine-Induced Gastrointestinal Dysmotility in the Rat. Front. Pharmacol. 8, 37. 10.3389/fphar.2017.00037 28220074 PMC5292571

[B339] Vermeer C. J. C. Hiensch A. E. Cleenewerk L. May A. M. Eijkelkamp N. (2021). Neuro-immune Interactions in Paclitaxel-Induced Peripheral Neuropathy. Acta Oncol. 60, 1369–1382. 10.1080/0284186X.2021.1954241 34313190

[B340] Verweij J. Clavel M. Chevalier B. (1994). Paclitaxel (Taxol) and Docetaxel (Taxotere): Not Simply Two of a Kind. Ann. Oncol. 5, 495–505. 10.1093/oxfordjournals.annonc.a058903 7918121

[B341] Wafai L. Taher M. Jovanovska V. Bornstein J. C. Dass C. R. Nurgali K. (2013). Effects of Oxaliplatin on Mouse Myenteric Neurons and Colonic Motility. Front. Neurosci. 7, 30. 10.3389/fnins.2013.00030 23486839 PMC3594784

[B342] Wahdan S. A. El-Derany M. O. Abdel-Maged A. E. Azab S. S. (2020). Abrogating Doxorubicin-Induced Chemobrain by Immunomodulators IFN-Beta 1a or Infliximab: Insights to Neuroimmune Mechanistic Hallmarks. Neurochem. Int. 138, 104777. 10.1016/j.neuint.2020.104777 32479984

[B343] Walczak P. Janowski M. (2019). Chemobrain as a Product of Growing Success in Chemotherapy - Focus on Glia as Both a Victim and a Cure. Neuropsychiatry (London) 9, 2207–2216. 10.4172/Neuropsychiatry.1000565 31316584 PMC6635604

[B344] Wang J. Zhang X. S. Tao R. Zhang J. Liu L. Jiang Y. H. (2017). Upregulation of CX3CL1 Mediated by NF-κB Activation in Dorsal Root Ganglion Contributes to Peripheral Sensitization and Chronic Pain Induced by Oxaliplatin Administration. Mol. Pain 13, 1744806917726256. 10.1177/1744806917726256 28849713 PMC5580849

[B345] Wang J. T. Medress Z. A. Barres B. A. (2012). Axon Degeneration: Molecular Mechanisms of a Self-Destruction Pathway. J. Cell Biol. 196, 7–18. 10.1083/jcb.201108111 22232700 PMC3255986

[B346] Wang X. M. Walitt B. Saligan L. Tiwari A. F. Cheung C. W. Zhang Z. J. (2015). Chemobrain: a Critical Review and Causal Hypothesis of Link between Cytokines and Epigenetic Reprogramming Associated with Chemotherapy. Cytokine 72, 86–96. 10.1016/j.cyto.2014.12.006 25573802 PMC4750385

[B347] Wang Y. B. de Lartigue G. Page A. J. (2020). Dissecting the Role of Subtypes of Gastrointestinal Vagal Afferents. Front. Physiol. 11, 643. 10.3389/fphys.2020.00643 32595525 PMC7300233

[B348] Wardill H. R. Gibson R. J. Van Sebille Y. Z. Secombe K. R. Coller J. K. White I. A. (2016a). Irinotecan-Induced Gastrointestinal Dysfunction and Pain Are Mediated by Common TLR4-dependent Mechanisms. Mol. Cancer Ther. 15, 1376–1386. 10.1158/1535-7163.MCT-15-0990 27197307

[B349] Wardill H. R. Mander K. A. Van Sebille Y. Z. Gibson R. J. Logan R. M. Bowen J. M. (2016b). Cytokine-mediated Blood Brain Barrier Disruption as a Conduit for Cancer/chemotherapy-Associated Neurotoxicity and Cognitive Dysfunction. Int. J. Cancer 139, 2635–2645. 10.1002/ijc.30252 27367824

[B350] Was H. Barszcz K. Czarnecka J. Kowalczyk A. Bernas T. Uzarowska E. (2017). Bafilomycin A1 Triggers Proliferative Potential of Senescent Cancer Cells *In Vitro* and in NOD/SCID Mice. Oncotarget 8, 9303–9322. 10.18632/oncotarget.14066 28030837 PMC5354733

[B351] Was H. Czarnecka J. Kominek A. Barszcz K. Bernas T. Piwocka K. (2018). Some Chemotherapeutics-Treated colon Cancer Cells Display a Specific Phenotype Being a Combination of Stem-like and Senescent Cell Features. Cancer Biol. Ther. 19, 63–75. 10.1080/15384047.2017.1385675 29053388 PMC5790359

[B352] Wefel J. S. Lenzi R. Theriault R. Buzdar A. U. Cruickshank S. Meyers C. A. (2004). 'Chemobrain' in Breast Carcinoma?: a Prologue. Cancer 101, 466–475. 10.1002/cncr.20393 15274059

[B353] Wefel J. S. Schagen S. B. (2012). Chemotherapy-related Cognitive Dysfunction. Curr. Neurol. Neurosci. Rep. 12, 267–275. 10.1007/s11910-012-0264-9 22453825

[B354] Wefel J. S. Vardy J. Ahles T. Schagen S. B. (2011). International Cognition and Cancer Task Force Recommendations to Harmonise Studies of Cognitive Function in Patients with Cancer. Lancet Oncol. 12, 703–708. 10.1016/S1470-2045(10)70294-1 21354373

[B355] Wefel J. S. Vidrine D. J. Marani S. K. Swartz R. J. Veramonti T. L. Meyers C. A. (2014). A Prospective Study of Cognitive Function in Men with Non-seminomatous Germ Cell Tumors. Psychooncology 23, 626–633. 10.1002/pon.3453 24339329 PMC4066616

[B356] Wigmore P. (2013). The Effect of Systemic Chemotherapy on Neurogenesis, Plasticity and Memory. Curr. Top. Behav. Neurosci. 15, 211–240. 10.1007/7854_2012_235 23239468

[B357] Wolf S. Barton D. Kottschade L. Grothey A. Loprinzi C. (2008). Chemotherapy-induced Peripheral Neuropathy: Prevention and Treatment Strategies. Eur. J. Cancer 44, 1507–1515. 10.1016/j.ejca.2008.04.018 18571399

[B358] Wozniak K. M. Vornov J. J. Wu Y. Liu Y. Carozzi V. A. Rodriguez-Menendez V. (2018). Peripheral Neuropathy Induced by Microtubule-Targeted Chemotherapies: Insights into Acute Injury and Long-Term Recovery. Cancer Res. 78, 817–829. 10.1158/0008-5472.CAN-17-1467 29191802 PMC5811354

[B359] Xiao W. H. Zheng H. Bennett G. J. (2012). Characterization of Oxaliplatin-Induced Chronic Painful Peripheral Neuropathy in the Rat and Comparison with the Neuropathy Induced by Paclitaxel. Neuroscience 203, 194–206. 10.1016/j.neuroscience.2011.12.023 22200546 PMC3273641

[B360] Xu Y. L. Zhao W. H. Tang Z. Y. Li Z. Q. Long Y. Cheng P. (2019). Guillain-Barré Syndrome in a Patient with Multiple Myeloma after Bortezomib Therapy: A Case Report. World J. Clin. Cases 7, 2905–2909. 10.12998/wjcc.v7.i18.2905 31616710 PMC6789385

[B361] Yamakuni H. Sawai-Nakayama H. Imazumi K. Maeda Y. Matsuo M. Manda T. (2002). Resiniferatoxin Antagonizes Cisplatin-Induced Emesis in Dogs and Ferrets. Eur. J. Pharmacol. 442, 273–278. 10.1016/s0014-2999(02)01541-8 12065081

[B362] Yamamoto K. Nakai M. Nohara K. Yamatodani A. (2007). The Anti-cancer Drug-Induced pica in Rats Is Related to Their Clinical Emetogenic Potential. Eur. J. Pharmacol. 554, 34–39. 10.1016/j.ejphar.2006.09.058 17109847

[B363] Yamamoto S. Egashira N. (2021). Pathological Mechanisms of Bortezomib-Induced Peripheral Neuropathy. Int. J. Mol. Sci. 22, 1–14. 10.1016/j.cotox.2021.08.007 PMC7830235 33477371

[B364] Yang M. Kim J. S. Kim J. Jang S. Kim S. H. Kim J. C. (2012). Acute Treatment with Methotrexate Induces Hippocampal Dysfunction in a Mouse Model of Breast Cancer. Brain Res. Bull. 89, 50–56. 10.1016/j.brainresbull.2012.07.003 22796103

[B365] Yin Y. Qi X. Qiao Y. Liu H. Yan Z. Li H. (2018). The Association of Neuronal Stress with Activating Transcription Factor 3 in Dorsal Root Ganglion of *In Vivo* and *In Vitro* Models of Bortezomib- Induced Neuropathy. Curr. Cancer Drug Targets 19, 50–64. 10.2174/1568009618666181003170027 30289077

[B366] You Z. Zhang S. Shen S. Yang J. Ding W. Yang L. (2018). Cognitive Impairment in a Rat Model of Neuropathic Pain: Role of Hippocampal Microtubule Stability. Pain 159, 1518–1528. 10.1097/j.pain.0000000000001233 29613911 PMC6053326

[B367] Yu X. Yang J. Hou X. Zhang K. Qian W. Chen J. D. (2009). Cisplatin-induced Gastric Dysrhythmia and Emesis in Dogs and Possible Role of Gastric Electrical Stimulation. Dig. Dis. Sci. 54, 922–927. 10.1007/s10620-008-0470-0 18754094

[B368] Zabara J. (1992). Neuroinhibition in the Regulation of Emesis. Pharmacol. Toxicol. 63 (2), 70–74. 10.1111/j.1600-0773.1988.tb00913.x 2903494

[B369] Zajączkowska R. Kocot-Kępska M. Leppert W. Wrzosek A. Mika J. Wordliczek J. (2019). Mechanisms of Chemotherapy-Induced Peripheral Neuropathy. Int. J. Mol. Sci. 20, 1451. 10.3390/ijms20061451 PMC6471666 30909387

[B370] Zhang H. Li Y. De Carvalho-Barbosa M. Kavelaars A. Heijnen C. J. Albrecht P. J. (2016). Dorsal Root Ganglion Infiltration by Macrophages Contributes to Paclitaxel Chemotherapy-Induced Peripheral Neuropathy. J. Pain 17, 775–786. 10.1016/j.jpain.2016.02.011 26979998 PMC4939513

[B371] Zheng H. Xiao W. H. Bennett G. J. (2012). Mitotoxicity and Bortezomib-Induced Chronic Painful Peripheral Neuropathy. Exp. Neurol. 238, 225–234. 10.1016/j.expneurol.2012.08.023 22947198

